# The Internal Otic Region of Oromerycids (Artiodactyla, Oromerycidae), Early Camelids (Artiodactyla, Camelidae), and the Vicuña (Artiodactyla, Camelidae), Including Notes on Intraspecific and Subadult Ontogenetic Variation

**DOI:** 10.1093/iob/obaf043

**Published:** 2025-11-17

**Authors:** S V Robson, A Prokop, C N Baird, J A Ludtke, S T Tucker, J M Theodor

**Affiliations:** Leibniz-Institut zur Analyse des Biodiversitätswandels, Standort Hamburg, 20146 Hamburg, Germany; Department of Biological Sciences, University of Calgary, Calgary, AB T2N 1N4, Canada; Department of Biological Sciences, University of Calgary, Calgary, AB T2N 1N4, Canada; Richard Gilder Graduate School, American Museum of Natural History, 200 Central Park West, NY, NY 10024, USA; Lamont-Doherty Earth Observatory, Columbia University, 61 Rte. 9W, Palisades, NY 10964, USA; San Diego Miramar College, 10440 Black Mountain Road, San Diego, CA 92126, USA; Highway Paleontology Program, University of Nebraska State Museum, 645 North 14^th^ Street, Lincoln, NE 68588, USA; Department of Biological Sciences, University of Calgary, Calgary, AB T2N 1N4, Canada

## Abstract

The taxonomic composition of the suborder Tylopoda is an ongoing debate. Recently, the internal otic region (petrosal and bony labyrinth) has been intensively studied as a source of additional morphological data, but the morphology of this region in extinct tylopods is not well documented. To remedy this, we used µCT scanning to image and describe the petrosal and bony labyrinth of two oromerycids (*Protylopus, Eotylopus*), four early camelids (*Poebrotherium wilsoni, Poebrotherium eximium, Paratylopus primaevus, Stevenscamelus franki*), and the living vicuña (*Vicugna vicugna*). Several early camelid specimens also preserved the ear ossicles (malleus, incus, stapes), enabling us to describe their morphology for the first time. Our sample allows us to not only compare among taxa, but to also examine variation within taxa and during ontogeny. We found that the morphology of the petrosal is far more variable than that of the bony labyrinth, both within and across taxa. There is no notable ontogenetic variation between the juveniles and adults in our sample. *Protylopus* has an unusual petrosal morphology, and its bony labyrinth is somewhat reminiscent of early dichobunoid artiodactyls. Conversely, *Eotylopus* has a transitional morphology that seemingly links it to camelids. *Poebrotherium wilsoni* and *Po. eximium* do not noticeably differ in their morphology, but there are identifiable differences in *Pa. primaevus* and *S. franki*, suggesting that the petrosal of camelids is diagnostic at a genus level. The early camelids were distinct from the vicuña in petrosal, bony labyrinth, and ossicular chain morphology, highlighting the importance of examining basally branching members to resolve evolutionary relationships.

## French

La composition taxonomique du sous-ordre des Tylopodes fait l’objet de débats persistants. Récemment, la région otique interne (noyau pétreux et labyrinthe osseux) a été étudiée en profondeur comme source de données morphologiques complémentaires, mais la morphologie de cette région chez les tylopodes éteints est encore mal documentée. Afin de pallier ce manque, nous avons utilisé la microtomographie à rayons X (µCT) pour imager et décrire le noyau pétreux et le labyrinthe osseux de deux oroméricidés (*Protylopus, Eotylopus*), de quatre camélidés primitifs *(Poebrotherium wilsoni, Poebrotherium eximium, Paratylopus primaevus, Stevenscamelus franki*) et de la vigogne (*Vicugna vicugna*). Plusieurs spécimens de camélidés primitifs ont également conservé les osselets de l’oreille moyenne (marteau, enclume, étrier), ce qui nous a permis de décrire leur morphologie pour la première fois. Notre échantillon nous permet non seulement de comparer les taxons entre eux, mais aussi d’examiner la variation au sein d’un même taxon et au cours de l’ontogenèse. Nous avons constaté que la morphologie du pétreux est beaucoup plus variable que celle du labyrinthe osseux, tant au sein d’un même taxon qu’entre différents taxons. Aucune variation ontogénétique notable n’a été observée entre les juvéniles et les adultes de notre échantillon. *Protylopus* présente une morphologie pétreuse inhabituelle, et son labyrinthe osseux rappelle quelque peu celui des premiers artiodactyles dichobunoïdes. À l’inverse, *Eotylopus* possède une morphologie transitionnelle qui semble le rattacher aux camélidés. *Poebrotherium wilsoni* et *Po. eximium* ne présentent pas de différences morphologiques notables, mais des différences identifiables existent chez *Pa. primaevus* et *S. franki*, suggérant que le pétreux des camélidés est un critère diagnostique au niveau du genre. Les premiers camélidés se distinguaient des vigognes par la morphologie de la région pétreuse, du labyrinthe osseux et de la chaîne ossiculaire, soulignant l’importance d’examiner les membres à ramification basale pour résoudre les relations évolutives.

## German

Die taxonomische Zusammensetzung der Unterordnung Tylopoda ist Gegenstand einer fortdauernden Debatte. In jüngerer Zeit wurde die innere otische Region (Petrosum und knöchernes Labyrinth) intensiv als Quelle zusätzlicher morphologischer Daten untersucht, aber die Morphologie dieses Bereichs ist bei ausgestorbenen Tylopoden nur unzureichend dokumentiert. Um dies zu beheben, haben wir mittels µCT-Scans das Petrosum und das knöcherne Labyrinth von zwei Oromeryciden (*Protylopus, Eotylopus*), vier frühen Cameliden (*Poebrotherium wilsoni, Poebrotherium eximium, Paratylopus primaevus, Stevenscamelus franki*) sowie vom lebenden Vikunja (*Vicugna vicugna*) erfasst und beschrieben. In mehreren frühen Cameliden-Exemplaren sind zudem die Gehörknöchelchen (Malleus, Incus, Stapes) gut erhalten, was es uns ermöglichte, deren Morphologie erstmals zu beschreiben. Unser Material erlaubt uns nicht nur Vergleiche zwischen Taxa durchzuführen, sondern auch die Untersuchung der Variation innerhalb von Taxa und im Verlauf der Ontogenese. Wir fanden, dass die Morphologie des Petrosums sowohl innerhalb als auch zwischen Taxa deutlich variabler ist als die des knöchernen Labyrinths. Zwischen den juvenilen und adulten Individuen unseres Materials besteht keine nennenswerte ontogenetische Variation. *Protylopus* weist eine ungewöhnliche Petrosum-Morphologie auf, und sein knöchernes Labyrinth erinnert an das früher dichobunoider Paarhufer*. Eotylopus* zeigt eine Übergangsmorphologie, die scheinbar eine Verbindung zu den Cameliden herstellt. *Poebrotherium wilsoni* und *Po. eximium* unterscheiden sich in ihrer Morphologie nicht auffällig, wohingegen in *Pa. primaevus* und *S. franki* erkennbare Unterschiede bestehen. Dies deutet darauf hin, dass das Petrosum der Cameliden auf Gattungsebene diagnostisch ist. Die frühen Cameliden unterscheiden sich vom Vikunja in der Morphologie von Petrosum, knöchernem Labyrinth und Ossikelkette, was die Bedeutung der Untersuchung basal abzweigender Vertreter für die Auflösung evolutiver Beziehungen unterstreicht.

## Introduction

**Fig. 1:**
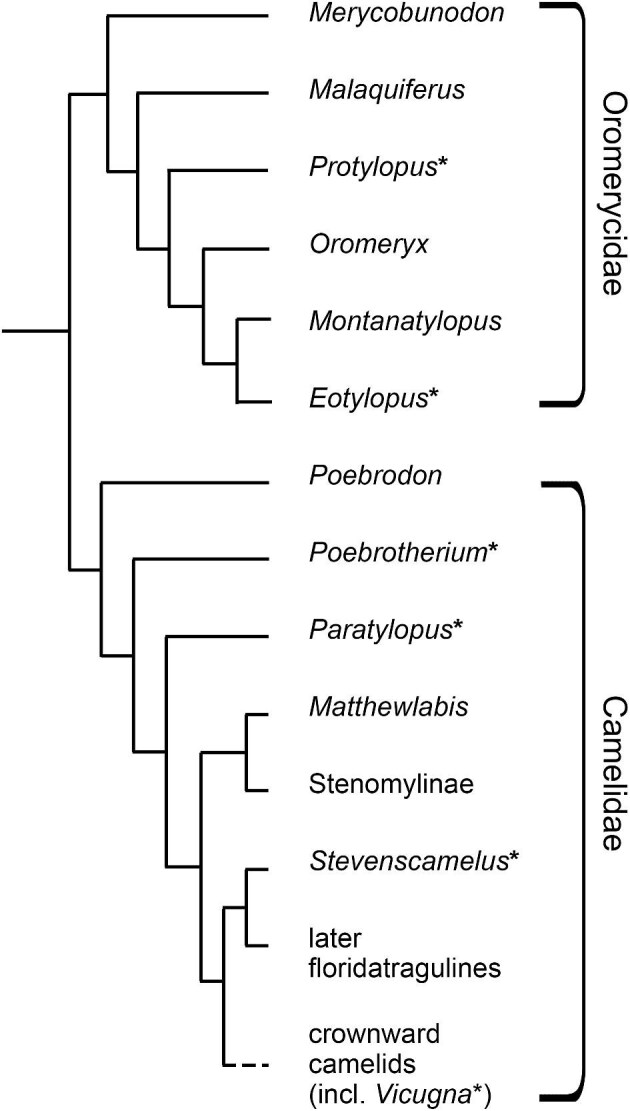
Hypothesized evolutionary relationships of oromerycids and early camelids, based on cladograms proposed by Prothero (1998a) and Honey et al. (1998). * Indicates genera included in this study.

The taxonomic composition of the artiodactyl suborder Tylopoda is a long-standing problem in the literature. Camelids are the only definitive tylopods, but several extinct North American and European families have been referred to the clade, often based on limited evidence, which has resulted in an ongoing debate (e.g., Gentry and Hooker 1988; Joeckel and Stavas 1996; Janis et al. 1998; Stucky 1998; Prothero 1998a, 1998b; Norris 1999, 2000; Ludtke 2007; Prothero and Ludtke 2007). So far, phylogenetic analyses have been unable to satisfactorily resolve the composition of the suborder (e.g., Gentry and Hooker 1988; Gatesy and O’Leary 2001; Geisler 2001; Theodor and Foss 2005; Thewissen et al. 2007; O’Leary and Gatesy 2008; Spaulding et al. 2009 and references therein), suggesting that additional lines of evidence are needed. The internal otic region is becoming increasingly important for understanding terrestrial artiodactyl evolutionary relationships, in part because of its morphological complexity, developmental constraints, and association with hearing and balance (e.g., Joeckel and Stavas 1996; Norris 1999, 2000; O’Leary 2010; Orliac 2013; Orliac and O’Leary 2014; Mennecart et al. 2016, 2017; Mennecart and Costeur 2016a; Orliac et al. 2017, 2023; Robson et al. 2021, 2022; Robson and Theodor 2025). It may serve to help resolve relationships within Tylopoda, but our knowledge of the tylopod otic region is sorely lacking. This is particularly striking when considering Oromerycidae, likely close relatives to Camelidae known from the Eocene of western North America (Wilson 1974; Gentry and Hooker 1988; Prothero 1996, 1998a). To our knowledge, there are no published descriptions of the internal otic region of oromerycids, and the internal otic region morphology of only one early camelid (*Poebrotherium*) is known. This lack of data poses a problem given that oromerycids and early diverging camelids likely represent an important morphological transition between early tylopods and living camelids. Furthermore, changes in otic region morphology have occurred within other tylopod families, affecting phylogenetic inferences and highlighting the importance of sampling both early- and late-branching taxa (Whitmore 1953; Joeckel and Stavas 1996; Norris 2000; O’Leary 2010; Robson et al. 2021, 2022). In this paper, based on data produced via micro-computed tomography (µCT) scanning, we describe the internal otic region of two oromerycid species (one early-branching and one late-branching) and four early camelid species, compare these taxa to the extant camelid *Vicugna vicugna*, and test whether there is morphological variation among individuals and between late-stage juveniles and adults (Fig. 1).

Oromerycids are a taxonomically small family with only six described genera (Prothero 1998a). The two oromerycids in our study are *Protylopus* cf. *Pr. stocki* (known from the middle Eocene) and *Eotylopus* cf. *E. reedi* (known from the middle and late Eocene). *Protylopus* holds a relatively basal position in the oromerycid phylogeny while *Eotylopus* is one of the latest-branching genera (Janis et al. 1998; Prothero 1998a) (Fig. 1). The inclusion of both *Protylopus* and *Eotylopus* allows for a broader examination of morphology within the family and provides multiple points of comparison to camelids. If these two families are closely related, we would expect *Protylopus* and *Eotylopus* to share some derived aspects of otic region morphology with camelids such as a ventromedial flange and a rostral tympanic process on the petrosal.

The four early camelids we describe are *Poebrotherium wilsoni, Poebrotherium eximium* (along with *Poebrotherium* sp.), *Paratylopus primaevus*, and *Stevenscamelus franki*. Sampling multiple taxa is necessary to establish which features of the otic region are shared among early-diverging camelids (Fig. 1). A cranium of *Po. wilsoni* was serially sectioned and described by Whitmore (1953), and a petrosal of *Poebrotherium* sp. was physically dissected from the skull and described by O’Leary (2010), so the overall petrosal morphology of *Poebrotherium* is known, but its bony labyrinth morphology has not been documented and comparisons among *Poebrotherium* species have not been made. Additionally, there has not been any study of closely related taxa. *Paratylopus primaevus* is an early camelid that co-occurs with species of *Poebrotherium* in the Eocene and Oligocene of North America (Fig. 1) (Honey et al. 1998). *Paratylopus* and *Poebrotherium* have a similar external morphology, primarily being distinguished by minor differences in skull and dental proportions (Prothero 1996). *Stevenscamelus franki* was, until recently, referred to *Poebrotherium* (Wilson 1974; Prothero 1996; Honey et al. 1998). It is also Eocene in age and is thought to be the earliest floridatraguline (Fig. 1), a camelid subfamily otherwise only represented by Miocene genera (Prothero et al. 2023). Together, these descriptions represent the first survey of camelid internal otic region morphology across closely related taxa. Given that the external otic region of early camelids does not greatly vary among taxa, it is likely that the same is true of the petrosal, bony labyrinth, and ear ossicles, but small differences may be present. If there are more pronounced differences, they will most probably be found in *Stevenscamelus franki*, the early floridatraguline.

As far as we know, our description of *Vicugna vicugna* is the first published report detailing the internal otic region of a laminin: although the petrosal of *Camelus dromedarius* has been described in detail (O’Leary 2010; Allouch et al. 2023), there has only been limited description of the otic region of South American camelids to date (Whitmore 1953; Joeckel and Stavas 1996; Norris 1999; Concha-Albornoz et al. 2012).

Descriptions of the petrosal of extinct taxa are often based on single specimens (O’Leary 2010; Theodor 2010; Orliac 2013; Orliac et al. 2013; Orliac and O’Leary 2014; Mennecart et al. 2016; Mennecart and Costeur 2016a; Robson et al. 2021, 2022), which poses a potential problem when these descriptions are treated as representative of a species or genus. Given that the petrosal forms in close association with several soft tissues (Macintyre 1972; Novacek 1977; MacPhee 1981), many of which are likely to exhibit variation, it should be expected that there will be morphological variation in the petrosal. Indeed, when multiple specimens are available for comparison, differences in the petrosal have been noted, but these differences are not consistent across clades (Costeur 2014; Orliac et al. 2017). The exceptional fossil record of *Poebrotherium* and the availability of *V. vicugna* specimens allows us to compare members of the same genus (*Poebrotherium* spp., N = 6) and species (*V. vicugna*, N = 5). Documenting intraspecific and intrageneric variation aids in determining which morphological features are most likely to be phylogenetically informative, including features that have been used in phylogenetic analyses such as the shape of the internal acoustic meatus and the position of the basicapsular groove (Spaulding et al. 2009; O’Leary 2010; Mourlam 2019; Robson et al. 2023).

Juveniles are commonly represented in the fossil record, and the specimen of the oromerycid *Eotylopus* cf. *E. reedi* used in this study is one such example—dP3/3 and dP4/4 are retained, and M3/3 are erupting. Juveniles can greatly increase the sample size of a study and, sometimes, a juvenile may be the first (or best) representative of a taxon, as is the case for the holotype of *Po. wilsoni* (Leidy 1847; Prothero 1996). Still, extra care must be taken when basing inferences on juvenile morphology: although the otic region is mostly ossified before birth and changes to the bony labyrinth are likely to be minimal (Mennecart and Costeur 2016b; Costeur et al. 2017), the morphology of the petrosal may change from bone accretion (Ekdale 2010; Costeur et al. 2017). To determine whether this is the case in tylopods, we compared the petrosal morphology of juvenile camelids to those of adults. We included an ontogenetic sequence of both an early-diverging camelid (*Poebrotherium* spp.) and an extant camelid (*V. vicugna*) for this purpose. A lack of variation in the petrosal and bony labyrinth between juveniles and adults, which is what we would expect based on ruminants, would suggest that the *Eotylopus* specimen can reasonably be compared to adult individuals, as can other juvenile tylopods of a similar growth stage.

With our data from oromerycids and camelids, we can more thoroughly examine changes in the petrosal, bony labyrinth, and ear ossicles of tylopods and clarify a few aspects of terminology. These data have implications for phylogenetic hypotheses, including which extinct taxa are most closely related to camelids. By including multiple juvenile and adult individuals we have been able to study ontogenetic, intraspecific, and intrageneric variation. Our data support the inclusion of older juveniles in comparative studies and reveal intraspecific differences that have hitherto gone undocumented.

## Materials and methods

### Abbreviations

**Institutional abbreviations: AMNH FM**, American Museum of Natural History, Frick Mammal Collection, New York City, NY, USA; **FMNH**, Field Museum of Natural History, Chicago, IL, USA; **MoJo**, Centre for Mobility and Joint Health, McCaig Institute for Bone and Joint Health, Cumming School of Medicine, University of Calgary, Calgary, AB, Canada; **ROM VPR**, Royal Ontario Museum Vertebrate Palaeontology, Toronto, ON, Canada; **SDSNH**, San Diego Natural History Museum, San Diego, CA, USA; **TMM VP**, Texas Vertebrate Paleontology Collection, Jackson Museum of Earth History, University of Texas Austin, TX, USA; **UCMZ (M)**, University of Calgary Museum of Comparative Zoology, Mammals Collection, Calgary, AB, Canada; **UNSM ZM**, University of Nebraska State Museum, Division of Zoology, Lincoln, NE, USA; **USNM**, United States National Museum, Division of Mammals, Washington D.C., USA; **UTCT**, University of Texas High-Resolution X-ray CT Facility, University of Texas Austin, TX, USA; **YPM PU**, Yale Peabody Museum, Princeton University Collection, Yale University, New Haven, CT, USA.

### Specimens and data collection

All specimens were imaged using µCT scanning, but scanners and parameters differ. The scan parameters for individual specimens can be found in Supplementary Material 1, and the relevant scanner makes and models are stated below with the specimens. The µCT data were segmented using either Thermo Scientific Amira Software (Amira 5.3 2010; Amira 6.0.1 2015) or the SlicerMorph module (developed by Rolfe, S. and Maga, M; Rolfe et al. 2021) in 3D Slicer (Kikinis et al. 2014; 3D Slicer 5.6.1 2023; 3D Slicer 5.6.2 2024; https://www.slicer.org/). The relevant program and version is also indicated with each specimen.

Scans were loaded into Amira at full resolution, but 3D Slicer struggled to handle the full datasets. Therefore, the data that were processed in 3D Slicer were loaded at half resolution and scans were trimmed to the region of interest using the Crop Volume module. To produce smoother surfaces, these trimmed datasets were resampled (using the same module) to a spacing scale of 0.5x with interpolated cropping and isotropic spacing. This resulted in a resampled voxel size identical to the native voxel size (see Supplementary Material 1 for native voxel sizes).

All segmentation was done manually with a digitizing tablet. In both Amira and 3D Slicer, a masking threshold was applied based on the relative grey-values of the pixels. Each scan had a different density distribution, so the thresholds were set on a case-by-case basis. All fossil specimens in Amira were segmented with the Brush tool. The recent *Vicugna* material was segmented using a combination of the Brush Tool and the Magic Wand Tool. Specimens in 3D Slicer were segmented with the Paint Tool. Digital models were produced in Amira with unconstrained smoothing and in 3D Slicer with a smoothing factor of 0.5. The digital models and underlying µCT data are reposited in MorphoSource (https://www.morphosource.org/) (see Data availability statement).

***Eotylopus* cf. *E. reedi*, AMNH FM 47394**—cranium, mandible, and associated postcrania, from the Chadron Formation of Bates Hole, Natrona County, Wyoming, Chadronian North American Land Mammal Age (NALMA) (late Eocene). Cranium includes roots of P1 and the teeth dP2-4, M1-2 with M3 erupting. Mandible has broken right i3 (left incisors missing), and on both sides canine and p1-2 erupting, dp3-4, m1-2, and m3 erupting. The specimen is an oromerycid based on its distally bifurcate protocone on M1-3 and the deep lingual notch between the m3 entoconid and hypoconulid (Wilson 1974; Prothero 1986, 1998a). The specimen is referable to *Eotylopus* based on its relatively large size, comparatively high-crowned molars, and its reduced enamel crenulation (Prothero 1998a). The specimen is smaller than *Montanatylopus* and has lower crowned molars (Prothero 1986)*. Eotylopus reedi* is the only named species of *Eotylopus* (Matthew 1910) and AMNH FM 47394 is similar in size to the holotype (Scott 1940). However, because we cannot adequately compare the specimens to make a confident species-level identification, we refer AMNH FM 47394 to *Eotylopus* sp. cf. *E. reedi* until more data are available. The specimen was scanned by S. V. Robson at the University of Calgary using a SkyScan 1173 shared between J. Anderson and J. M. Theodor. A ring artifact correction of 16 was applied post-scanning using the NRecon software by Bruker (NRecon v.1.6.9 2022). Data were processed in Amira 5.3 (2010).

***Paratylopus primaevus*, AMNH FM 9806**—see Matthew (1904, 1909) for referral and description, from the upper Brule Formation (Poleslide Member, formerly Upper Oreodon Beds), White River Group, South Dakota, Orellan to Whitneyan NALMA (Oligocene) (Matthew 1904; Bump 1956). AMNH FM 9806 was scanned by C. N. Baird at the American Museum of Natural History using a GE phoenix v|tome|x s240. The data were processed in Amira 5.3 (2010), except for the ear ossicles, which were processed in Amira 6.0.1 (2015).

***Poebrotherium eximium*, AMNH FM 42298**—cranium with the rostrum missing anterior to dP2. Right and left partial dP2, dP3-4 and M1 preserved. M2 erupting. Locality unknown. The specimen was referred to *Po. eximium* by Prothero (1996). This specimen was scanned by S. V. Robson at the University of Calgary using a SkyScan 1173 shared between J. Anderson and J. M. Theodor. A ring artifact correction (16) and a beam hardening correction (20%) were applied during reconstruction using NRecon software (NRecon v.1.6.9 2022). The specimen was processed in Amira 6.0.1 (2015).

***Poebrotherium eximium*, AMNH FM 47077**—complete adult cranium, mandible, and associated postcrania from Spring Draw, Southeast Seaman Hills, Niobrara County, Wyoming, Chadronian NALMA (late Eocene). The specimen was referred to *P. eximium* by Prothero (1996). AMNH FM 47077 was scanned by S. V. Robson at the University of Calgary using a SkyScan 1173 shared between J. Anderson and J. M. Theodor. A ring artifact correction of 10 was applied during reconstruction with NRecon software v.1.6.9 (2022). Petrosals and bony labyrinths were processed in Amira 5.3 (2010). Ear ossicles were processed in Amira 6.0.1 (2015).

***Poebrotherium* sp., AMNH FM 147015**—cranium with the rostrum missing anterior to dP3, partial dP3s preserved, dP4-M2 present with M3 erupting. The specimen is from Herman Wolff Ranch, White River Group, Converse County, Wyoming, Chadronian to Whitneyan NALMA (late Eocene to early Oligocene). The specimen is referable to *Poebrotherium* based on the characteristic inflated auditory bullae of camelids and its small size and lower crowned teeth compared to other early camelids such a *Matthewlabis* (Honey et al.1998; Prothero 1996). S. V. Robson scanned this specimen at the University of Calgary using a SkyScan 1173 shared between J. Anderson and J. M. Theodor. NRecon software was used to apply a ring artifact correction (16) and beam hardening correction (50%) post-scanning (NRecon v.1.6.9 2022). The petrosal data were processed in 3D Slicer 5.6.1 (2023) and 5.6.2 (2024). The bony labyrinths were processed in Amira 5.3 (2010) and the ear ossicles were processed in Amira 6.0.1 (2015).

***Poebrotherium* sp., FMNH PM 14560**—adult cranium and lower jaws, intact except for the upper rostrum anterior to I3 and right condyle and coronoid process of the mandible. The specimen is from the Orella Member of the Brule Formation, White River Group, Sioux County, Nebraska, Orellan NALMA (early Oligocene). Based on the characteristic inflated auditory bullae, low-crowned teeth, and overall size, the specimen is referable to either *Poebrotherium* or *Paratylopus*, both early camelids with a similar dental morphology. The genera are primarily distinguished based on postcranial morphology (Prothero 1996), and no postcrania are associated with FMNH PM 14560. Prothero (1996) suggested that the genera can also be differentiated based on the length of the upper molar row and the amount of premolar reduction. However, a larger sample of specimens referred to *Poebrotherium* and *Paratylopus* reveals that there is considerable overlap in both size and amount of premolar reduction, and FMNH PM 14560 falls within the ranges of both genera (Supplementary Material 2). We refer the specimen to *Poebrotherium* rather than *Paratylopus* because the skull is relatively gracile and the buccal wall of M3 is lingually inflected, a morphology we have observed in several specimens of *Poebrotherium* but not in *Paratylopus*.

We refrain from assigning FMNH PM 14560 to a species. The specimen cannot be referred to *Poebrotherium chadronense* because of its larger size and lack of a posterolingual cusp on P3 (Prothero 1996), but it shows an unusual suite of dental features that are reminiscent of both *Po. wilsoni* and *Po. eximium*. The I3 is enlarged and there is a long diastema between the upper canine and P1, both of which are diagnostic of *Poebrotherium wilsoni*, but the diastema between P1 and P2 is shorter than what is typical for *Po. wilsoni*, and some specimens of *Po. eximium* have a similarly short diastema (Supplementary Material 3, Fig. S1A) (Prothero 1996). The lower i1-2 are arranged in a closed “fan” at the apex of the symphysis, which is common in *Po. wilsoni*, and the canine is incisiform but slightly larger than the incisors (Supplementary Material 3, Fig. S1B-C) (Prothero 1996). However, both *Po. wilsoni* and *Po. eximium* have a leaf-like (or spatulate) lower i3 that is continuous with the toothrow. Based on photographs, FMNH PM 14560 has an incisiform i3 that is not leaf-like or spatulate, and it is clearly separated from both i2 and the canine despite being in the same orientation as i1-2 (Supplementary Material 3, Fig. S1B-C): to our knowledge, this is not a morphology previously found in early camelids. There is a short diastema between the canine and first premolar, and a long diastema between p1 and p2. The m3 hypoconulid is narrow, particularly rostrally, and it resembles that of worn *Po. eximium* specimens with a crested hypoconulid (Supplementary Material 3, Fig. S1B), but this morphology can also be present in *Po. wilsoni* (Prothero 1996). Taken together, FMNH PM 14560 has morphological elements of both *Po. wilsoni* and *Po. eximium*. Based on age alone, the specimen would be referred to *Po. wilsoni* because specimens of *Po. eximium* are not known from the Orellan (Prothero and Emry 2004), but given the uncommon morphology of the lower incisor row, we do not consider FMNH PM 14560 to be referable to a species based on the available information.

FMNH PM 14560 was scanned by A. Neander at the University of Chicago PaleoCT Scanner Facility (RRID:SCR_0247) using a GE Phoenix v|tome|x s240 with a dual 180 tube. Datos software was used to apply a beam hardening correction of level 7 during reconstruction (Phoenix Datos|x v. 2.3.3 2021). The petrosals and bony labyrinths were processed in Amira 5.3 (2010), and the ear ossicles were processed in Amira 6.0.1 (2015).

***Poebrotherium wilsoni*, FMNH UM 465**—nearly complete cranium and lower jaws with adult dentition from the Orella Member of the Brule Formation, White River Group, Sioux County, Nebraska, Orellan NALMA (early Oligocene). The specimen is referred to *Po. wilsoni* for the same reasons as FMNH UM 493 (see Robson and Theodor 2025). This specimen was scanned by A. Neander at the University of Chicago PaleoCT Scanner Facility (RRID:SCR_0247) using a GE Phoenix v|tome|x s240 with a dual 180 tube. During reconstruction, Datos software was used to apply a beam hardening correction of level 6 (Phoenix Datos|x v. 2.3.3 2021). Data processing was conducted in Amira 5.3 (2010), aside from the ear ossicles, which were processed in Amira 6.0.1 (2015).

***Poebrotherium wilsoni*, FMNH UM 493**—juvenile cranium. See Robson and Theodor (2025) for complete description and scanning details. A level 7 beam hardening correction was used by Datos during reconstruction (Phoenix Datos|x v. 2.3.3 2021). The petrosals and bony labyrinths were processed in Amira 5.3 (2010), and the ear ossicles were processed in Amira 6.0.1 (2015).

***Protylopus* sp., SDSNH 40812**—cranium and associated postcrania, from member C of the Santiago Formation, California, late Uintan NALMA (middle Eocene). Identified by T. A. Deméré, the specimen is referable to *Protylopus* based on the presence of rectangular molars that do not narrow posteriorly, and the presence of a distally bifurcate protocone on the upper molars (Prothero 1998a). SDSNH 40812 was scanned by J. A. Ludtke at the University of Calgary using a SkyScan 1173 shared between J. Anderson and J. M. Theodor. Both ring artifact (16) and beam hardening (20%) corrections were applied with Bruker’s NRecon software (NRecon v. 1.6.6 2012). The data were processed in Amira 5.3 (2010).

***Protylopus* cf. *Pr. stocki*, SDSNH 60369**—partial cranium with left and right P3-M3, from the Mission Valley Formation, California, late Uintan NALMA (middle Eocene). Identified by S. L. Walsh, the specimen is referable to *Protylopus* based on the presence of rectangular molars that do not narrow posteriorly, and the presence of a distally bifurcate protocone on P4 and M1-3 (Prothero 1998a). The specimen is referred to *Protylopus* cf. *Pr. stocki* because of the relatively small lingual cingulum on P3-M3 compared to other species of *Protylopus* (Golz 1976). The specimen was scanned by J. A. Ludtke at the University of Calgary using a SkyScan 1173 shared between J. Anderson and J. M. Theodor. A ring artifact correction of 8 and a beam hardening correction of 40% were applied by NRecon software during reconstruction (NRecon v. 1.6.6 2012). Data were processed in Amira 5.3 (2010).

***Stevenscamelus franki*, TMM VP 40504–149**—see Wilson (1974) and Prothero et al. (2023) for referral and description. TMM VP 40504–149 was scanned by M. Colbert at the University of Texas High-Resolution X-Ray CT Facility (UTCT) using an NSI scanning system. A beam hardening correction of 0.1 was applied with efX-ct software by North Star Imaging, Inc. (EfX-CT 2023). The petrosals and bony labyrinths were processed in Amira 5.3 (2010), and the ear ossicles were processed in Amira 6.0.1 (2015).

***Vicugna vicugna*, UCMZ (M) 1986.307**—recent adult cranium and lower jaws. Female. The specimen was scanned by A. Cooke at the University of Calgary MoJo facility using a Scanco Medical AG XtremeCT (I and II). Data were processed in Amira 5.3 (2010)

***Vicugna vicugna*, UCMZ (M) 1986.308**—recent juvenile cranium and lower jaws with dP3-4 retained and M3 erupting. Sex unknown. UCMZ (M) 1987.308 was scanned by A. Cooke at the University of Calgary MoJo facility using a Scanco Medical AG XtremeCT (I and II). The petrosals and bony labyrinths were processed in Amira 5.3 (2010). The ear ossicles were processed in Amira 6.0.1 (2015).

***Vicugna vicugna*, UCMZ (M) 1986.309**—recent adult cranium and lower jaws. Male. A. Cooke scanned the specimen at the University of Calgary MoJo facility using a Scanco Medical AG XtremeCT (I and II). The petrosals and bony labyrinths were processed in Amira 5.3 (2010). The ear ossicles were processed in Amira 6.0.1 (2015).

***Vicugna vicugna*, UCMZ (M) 1986.310**—recent juvenile cranium and lower jaws with dP3-4 retained and M3 erupting. Sex unknown. This specimen was scanned by A. Cooke at the University of Calgary MoJo facility using a Scanco Medical AG XtremeCT (I and II). The data were processed in Amira 5.3 (2010).

***Vicugna vicugna*, UNSM ZM-16921**—recent adult (2–2.5 years old) cranium, braincase removed, auditory region intact. Male. Potentially hybridized with *Lama guanicoe* based on collections records. This potential hybridization is addressed in our description and discussion. UNSM ZM-16921 was scanned by M. McConnell and S. T. Tucker at the University of Nebraska-Lincoln NanoEngineering Research Core Facility using a Nikon Metrology XT H 225 ST. The petrosals and bony labyrinths were processed in Amira 5.3 (2010), and the ear ossicles were processed in Amira 6.0.1 (2015).

### Ontogenetic comparisons

Juveniles referred to *Poebrotherium* include one specimen of *Po. eximium* (AMNH FM 42298), one specimen of *Po. wilsoni* (FMNH UM 493), and one specimen referable to *Poebrotherium* sp. (AMNH FM 147015). These were compared with adults of *Po. wilsoni* (FMNH UM 465), *Po. eximium* (AMNH FM 47077), and a specimen referred to *Poebrotherium* sp. (FMNH PM 14560). Similarly, the two juvenile representatives of *V. vicugna* (UCMZ (M) 1986.308; UCMZ (M) 1986.310) were compared to two adult *V. vicugna* specimens (UCMZ (M) 1986.307; UCMZ (M) 1996.309) and, cautiously, one potentially hybridized *V. vicugna* x *Lama guanicoe* specimen (UNSM ZM-16921). Differences between UNSM ZM-16921 and the juvenile vicuñas were considered in light of the potential hybridization. All juveniles are at an earlier or equivalent growth-stage, based on dental eruption patterns, to the juvenile specimen of *Eotylopus* cf. *E. reedi* (Table 1). The latter retains some highly worn deciduous premolars with M3/3 erupting, which is equivalent to the eruption stage of the juvenile *Poebrotherium* sp., *Po. wilsoni*, and *Vicugna* specimens. The juvenile *Po. eximium* specimen is at a slightly earlier growth stage wherein M2 is erupting.

**Table 1:** Ontogenetic stages of the specimens in this study based on dental eruption sequence. From youngest to oldest, the stages are Juvenile 1, Juvenile 2, Adult

**Taxon**	**Specimen**	**Dentition**	**Stage**
*E.* cf. *E. reedi*	AMNH FM 47394	dP2-4, M1-2, M3 erupting; p1-2 erupting, dp3-4, m1-2, m3 erupting	Juvenile 2
*Pa. primaevus*	AMNH FM 9806	Adult dentition	Adult
*Po. eximium*	AMNH FM 42298	dP2-4, M1, M2 erupting	Juvenile 1
*Po. eximium*	AMNH FM 47077	Adult dentition	Adult
*Poebrotherium* sp.	AMNH FM 147015	dP3-dP4, M1-M2, M3 erupting	Juvenile 2
*Poebrotherium* sp.	FMNH PM 14560	Adult dentition	Adult
*Po. wilsoni*	FMNH UC 456	Adult dentition	Adult
*Po. wilsoni*	FMNH UC 493	dP2-4, M1-2, M3 erupting	Juvenile 2
*Protylopus* sp.	SDSNH 40812	Adult dentition	Adult
*Pro.* cf. *P. stocki*	SDSNH 60369	Adult dentition	Adult
*S. franki*	TMM VP 40504–149	Adult dentition	Adult
*V. vicugna*	UCMZ (M) 1986.307	Adult dentition	Adult
*V. vicugna*	UCMZ (M) 1986.308	dP3-dP4, M1-M2, M3 erupting	Juvenile 2
*V. vicugna*	UCMZ (M) 1986.309	Adult dentition	Adult
*V. vicugna*	UCMZ (M) 1986.3010	dP3-dP4, M1-M2, M3 erupting	Juvenile 2
*V. vicugna*	UNSM ZM-16921	Adult dentition	Adult

### Terminology

The petrosal terminology used here primarily comes from MacPhee (1981), O’Leary (2010), Orliac and O’Leary (2014), and the sources cited in those papers, with the exception of the “medial process of the epitympanic wing” and the “ventromedial flange,” which are discussed in Robson and Theodor (2025). We also distinguish between the fenestra vestibuli—the membrane-covered oval window—and the vestibular fossula, a depression surrounding the fenestra vestibuli that holds the annular ligament in life (MacPhee 1981). Terminology for the bony labyrinth is derived from Ekdale (2013). Terminology for the ear ossicles comes from Doran (1878), Wible and Spaulding (2012), Assemat et al. (2020), and the sources cited therein. We refer to the area measured for the fenestra vestibuli of the bony labyrinth as the “fenestra vestibuli area” rather than the “stapedial footplate area” because Orliac and Billet (2016) have demonstrated that the stapedial footplate is often much smaller than the fenestra vestibuli. Our stapedial footplate area is the area measured from the stapedial footplate of the stapes. When another terminology source is used, it is cited in the text. Revisions to terminology are presented in the Results and departures from prior usage are flagged.

### Measurements and calculations

Linear measurements were taken with the built-in 3D Slicer Create Line tool. These include the length of the cochlear aqueduct, the height and width of the semicircular canals, the length the common crus, the width of the crista interfenestralis, and the length (anteroposterior) and width (mediolateral) of the fenestra vestibuli and the vestibular fossula. The length of the cochlear aqueduct and the height and widths of the semicircular canals were measured from the bony labyrinth. The other measurements were taken from the petrosal. When the fenestra vestibuli and vestibular fossula were indistinguishable, the vestibular fossula measurements were taken from the external border of the aperture and the fenestra vestibuli measurements were taken from the internal border. These instances are recorded in Supplementary Material 3 and flagged in Supplementary Material 4. The crista interfenestralis width was measured from the border of the vestibular fossula, not the fenestra vestibuli. The arc radii of curvature of the semicircular canals were calculated based on the method in Spoor and Zonneveld (1995) adapted to 3D digital models. The height and width of each canal (measured from the center of the lumen) were used in the equation: radius = (height + width)/4.

The length of the cochlea was taken with the 3D Slicer Create Curve tool by measuring along the centerline of the coil from the beginning of the secondary bony lamina to the apex. When the true apical end of the canal could not be identified, the measurement was terminated at the most prominent part of the apex and the cochlear length was reported as an approximation or a minimum value, depending on the level of uncertainty (see Supplementary Material 3).

The fenestra vestibuli area was measured with the 3D Slicer Close Curve tool by circumscribing the innermost border of the fenestra on the petrosal. The stapedial footplate area was measured with the same tool by circumscribing the border of the stapedial footplate of the stapes.

Angles between semicircular canals were measured by first fitting a plane to each canal using the 3D Slicer Create Plane tool (plane type: three points), taking a screenshot of the intersection between the planes, and then measuring the angle between the planes using the Angle tool in Fiji (Schindelin et al. 2012). A similar method was used to measure the angle between the lateral semicircular canal and the basal turn of the cochlea. The plane through the midline of the basal cochlear turn was fit using the point on the basal turn adjacent to the fenestra cochleae, the point on the basal turn farthest from the vestibule, and the point on the basal turn opposite the fenestra cochleae. Additional details about this type of measurement can be found in Robson and Theodor (2025).

The number of cochlear turns was determined by using the Line tool in Fiji (Schindelin et al. 2012) and the methods of Ekdale and Rowe (2011) (and papers cited therein). This involves drawing a line from the start of the secondary bony lamina (corresponding to the laminar gap) through the cochlear axis of rotation, counting the number of times the cochlear canal crosses that line, multiplying the number by 180°, and then adding the angle between the line and the most apical point of the cochlear canal. This value can then be divided by 360° to produce the total number of turns. When the apex of the cochlea was indistinct, the precise number of turns could not be counted, and a minimum value was determined based on the number of times the cochlea definitively crossed the drawn line. If the more apical cochlear coils were entirely indistinct or missing, only this number was reported. If the apex was partially preserved but the end of the cochlear canal could not be confidently identified, a maximum number of turns was calculated based on the last definitive time the cochlea crossed the drawn line plus either 180° or 360°, depending on the level of uncertainty.

The cochlear aspect ratio (height/width) was also calculated with measurements made by the Line tool in Fiji (Schindelin et al. 2012), following Ekdale and Rowe (2011) and papers cited therein. The width of the cochlea was measured from the vestibular edge of the fenestra cochleae to the opposite wall of the basal turn. The height of the cochlea was measured from the tympanal edge of the fenestra cochleae to the apical turn of the cochlea, perpendicular to the plane of the basal turn.

Whenever possible, we measured both the right and left element and calculated an average value. To ensure accuracy, we repeated each measurement three times. Most specimens were exclusively measured by SVR with (based on AMNH FM 42298) a maximum standard deviation of 0.08 mm. Some measurements of the UCMZ *Vicugna* material were taken by both CNB and SVR. Measurements did not greatly differ between the two observers, and both were used to compute the average values. When an element was clearly deformed, we did not measure it. Retrodeformation was not applied to our specimens. Notes about measurements of individual specimens can be found in Supplementary Material 3, and measurements with uncertainty are flagged in Supplementary Material 4.

## Results

### Terminology clarification

#### Lateral process of the epitympanic wing

**Fig. 2:**
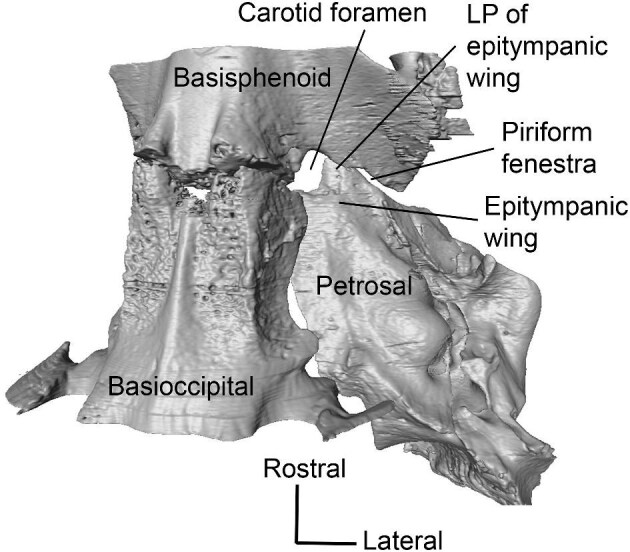
Digital reconstruction of the basicranium of *Poebrotherium wilsoni* (FMNH UM 493) in ventral view with the auditory bulla removed, exposing the carotid foramen and piriform fenestra. **Abbreviations: LP**, lateral process. Image not to scale.

The lateral process of the epitympanic wing of the petrosal is a bony projection that contributes to the border of a basicranial foramen (Orliac and O’Leary 2014). Early artiodactyls have a single foramen anterior to their promontorium that is the confluence between the carotid foramen and piriform fenestra. Orliac and O’Leary (2014) cautiously identified this foramen as the piriform fenestra, leading them to define the lateral process of the epitympanic wing as a process that caudally and lateral borders the piriform fenestra. In later branching artiodactyls, the carotid foramen and piriform fenestra are separate (O’Leary 2016). Based on figures in O’Leary (2016) and our observations here, when there is a separation, the lateral process (LP) of the epitympanic wing forms the caudolateral border of the carotid foramen, not the piriform fenestra (Fig. 2). As such, we propose that the lateral process of the epitympanic wing is a process extending from the main epitympanic wing that caudolaterally borders the carotid foramen, recognizing that some taxa have a singular foramen that can be reasonably identified as either the carotid foramen or the piriform fenestra. In other words, if the carotid foramen and piriform fenestra are separate, the lateral process of the epitympanic wing caudolaterally borders the carotid foramen (Fig. 2). If there is a single confluent opening that transmits the internal carotid artery, the lateral process of the epitympanic wing caudolaterally borders this confluent opening. In instances where the basioccipital, basisphenoid, or alisphenoid form a large portion of the carotid foramen, the lateral process of the epitympanic wing may not contribute to both the caudal and lateral edges of the foramen, but the process can still be identified as a triangular projection that articulates with the anterolateral margin of the carotid foramen and lies lateral to the medial process of the epitympanic wing when the latter is present (Fig. 2). We use this revised definition herein and consider camelids to have a lateral process of the epitympanic wing.

#### Endocranial ridge

**Fig. 3:**
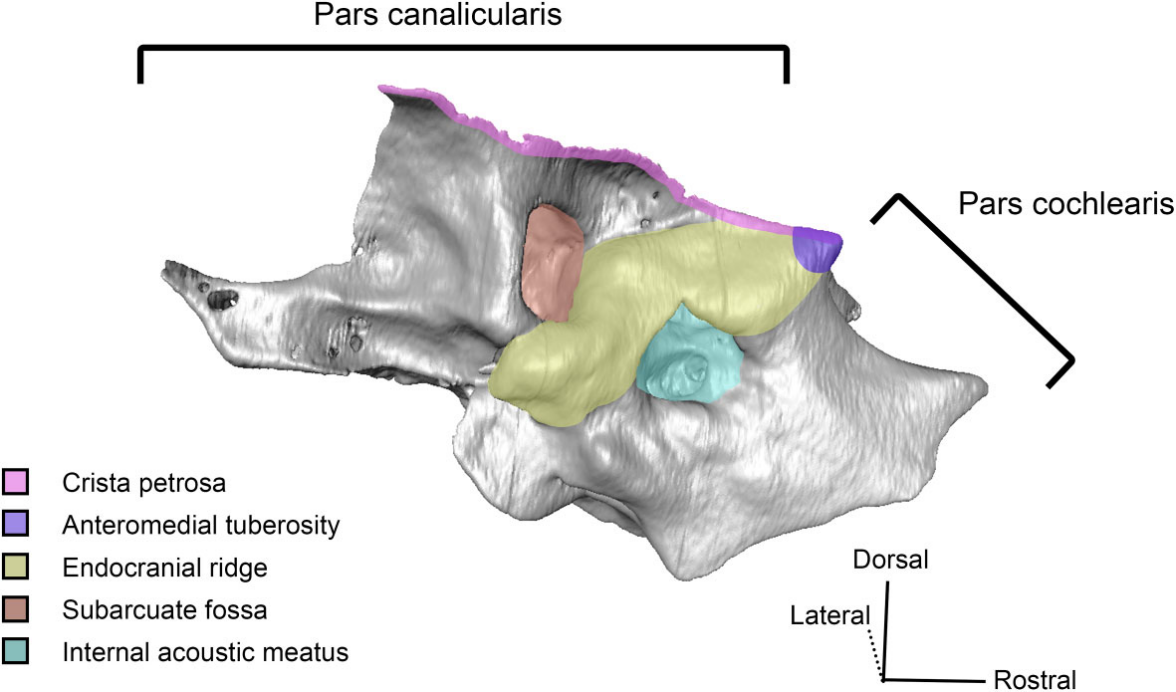
Digital reconstruction of the petrosal of *Vicugna vicugna* (UCMZ (M) 1986.309) in endocranial view showing the relationship of the endocranial ridge to adjacent structures. Note that the crista petrosa forms the dorsal border of the anteromedial tuberosity. Image not to scale.

The endocranial ridge is a bar of bone on the endocranial surface of the petrosal that delimits the border between the pars cochlearis (the part of the petrosal associated with the organ of hearing) and the pars canalicularis (the part of the petrosal enclosing the vestibular system), separating the internal acoustic meatus from the subarcuate fossa (Robson and Theodor 2025). This is not to be confused with the crista petrosa, which forms the dorsolateral border of the subarcuate fossa (see Fig. 3 for both structures). Unfortunately, when first introduced, this term was unintentionally used to synonymize two structures. It was based on Joeckel and Stavas (1996), who showed that the protoceratid *Syndyoceras* has a sharp change in orientation between the endocranial and dorsolateral (i.e., cerebellar and cerebral) faces of the petrosal, and Norris’s (2000) seeming misinterpretation of this morphology as a crest that traverses the endocranial face of the petrosal. Indeed, Norris (2000: Fig. 3B) depicted the endocranial ridge as dividing the subarcuate fossa from the internal acoustic meatus but wrote that it separates the cerebral and cerebellar faces. This is in error because both the internal acoustic meatus and the subarcuate fossa are associated with the cerebellum (Smuts and Bezuidenhout 1987; Gannon et al. 1988). Robson et al. (2021) followed Joeckel and Stavas (1996) when first defining the endocranial ridge, but Robson et al. (2022) and Robson and Theodor (2025) followed Norris’s (2000) diagram when identifying it on petrosals. This led to the term being used for both the bar of bone separating the endocranial pars cochlearis and pars canalicularis, and the crest separating the endocranial and dorsolateral faces of the petrosal. Robson et al. (2021: Fig. 6C) indicated that the camelid *Camelus dromedarius* lacks an endocranial ridge while Robson et al. (2022) stated that one is present in the early camelid *Poebrotherium*, causing the identity of the endocranial ridge and its presence in camelids to remain ambiguous. Here, we formally define the endocranial ridge as a bar of bone that divides the endocranial pars cochlearis from the endocranial pars canalicularis, and we confirm that all camelids we have examined possess such a structure (Fig. 3). The crest first reported by Joeckel and Stavas (1996) and depicted in Robson et al. (2021: Fig. 6A, B), which is absent in camelids, is not an endocranial ridge but rather the medial border of what appears to be a tegmen tympani fossa. A different term should be used for this structure.

#### Cochlear Fossula and Fenestra Cochleae

**Fig. 4:**
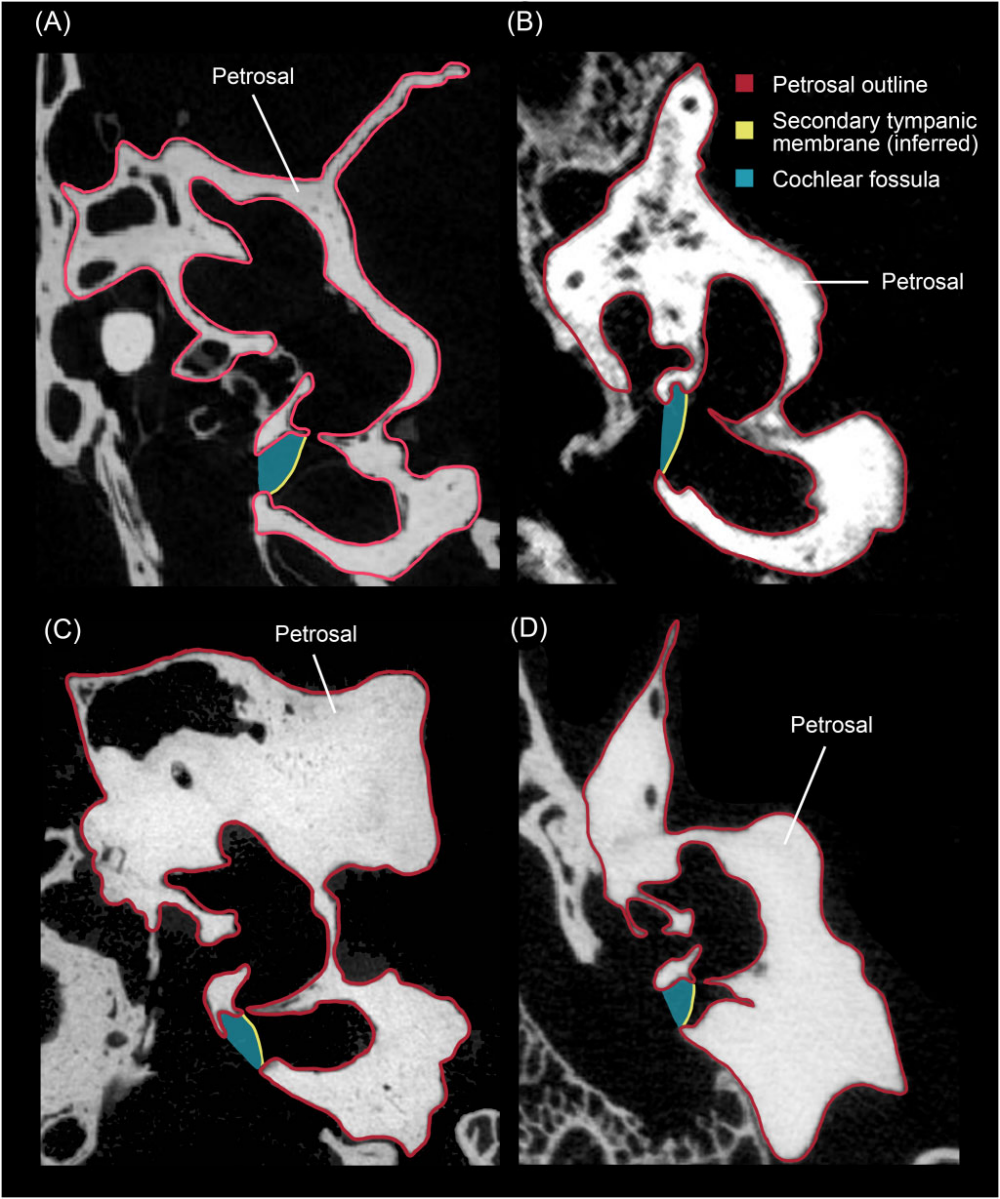
Transverse µCT slices showing the petrosal (outlined in red) of **A**, the scandentian *Tupaia glis* (USNM 487939, slice 1586); **B**, the suiform *Sus scrofa domestica* (Ashbaugh, unpublished data, slice 332); **C**, the ruminant *Antilocapra americana* (ROM VPR3969, slice 620); and **D**, the tylopod *Vicugna vicugna* (UCMZ (M) 1986.307, slice 587). The yellow line denotes the inferred location of the secondary tympanic membrane based on MacPhee (1981: fig. 38). The cochlear fossula is shown in blue. The µCT scan of USNM 487939 was downloaded from MorphoSource (doi:10.17602/M2/M15509). Images not to scale.

**Fig. 5:**
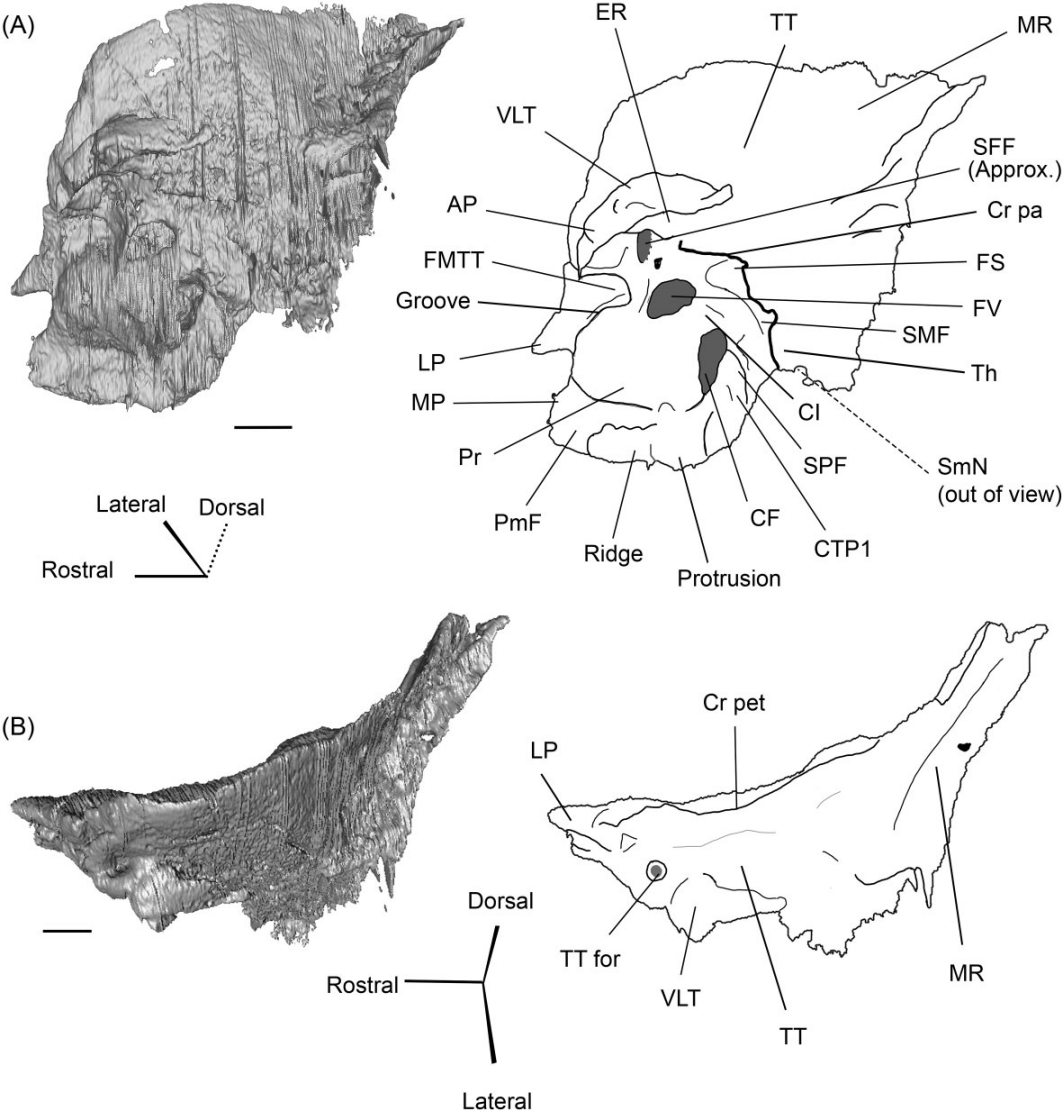
The right (mirrored) petrosal of the oromerycid *Protylopus* cf. *Pr. stocki* (SDSNH 60369) in tympanic **(A)** and dorsal **(B)** view. **Abbreviations: AP**, anterior process of the tegmen tympani; **Approx.**, approximate; **CF**, cochlear fossula; **CI**, crista interfenestralis; **CTP1**, medial caudal tympanic process; **Cr pa**, crista parotica; **Cr pet**, crista petrosa; **ER**, epitympanic recess; **FMTT**, fossa for the muscularis tensor tympani; **FS**, facial sulcus; **LP**, lateral process of the epitympanic wing; **MP**, medial process of the epitympanic wing; **MR**, mastoid region; **PmF**, posteromedial flange; **Pr**, promontorium; **SFF**, secondary facial foramen; **SMF**, stapedial muscle fossa; **SmN**, stylomastoid notch; **SPF**, saccus posticus fossa; **Th**, tympanohyal; **TT**, tegmen tympani; **TT for**, tegmen tympani foramen; **VLT**, ventrolateral tuberosity. Scale bars are 2 mm.

In the artiodactyl literature, the aperture in the petrosal associated with the membrane-covered round window is typically called the fenestra cochleae (O’Leary 2010; Theodor 2010; Orliac 2013; Orliac et al. 2013, 2017; Costeur 2014; Orliac and O’Leary 2014; Mennecart et al. 2016; Mennecart and Costeur 2016a; Robson et al. 2022, 2021; Robson and Theodor 2025). However, this terminology necessitates some refinement. MacPhee (1981) rightly pointed out that the identification of the fenestra cochleae on the face of the petrosal assumes that the secondary tympanic membrane lies across the cochlear aperture. In most eutherians, the membrane sits within the aperture, meaning that there is a fossula leading to the fenestra cochleae (Bast and Anson 1952; Frick 1952; Anson and Bast 1953; Donaldson 1968; Jollie 1968; MacPhee 1981; Wible et al. 2009; Billet and Muizon 2013; Orliac and O’Leary 2016; Wible and Shelley 2020; Gheerbrant et al. 2022). The aperture on the tympanic surface of the petrosal is not the true fenestra cochleae but rather the aperture of the cochlear fossula (MacPhee 1981). To the best of our knowledge, the precise location of the secondary tympanic membrane has not been described in artiodactyls, but based on a comparison between a µCT scan of the scandentian *Tupaia glis* (shown to have a cochlear fossula by MacPhee 1981), a pig (*Sus scrofa domesticus*), a ruminant (*Antilocapra americana*), and a camelid (*Vicugna vicugna*), it is clear that such a fossula is widespread in the order (Fig. 4). We choose to follow the convention established outside of the artiodactyl literature and identify the aperture on the tympanic face of the petrosal as the cochlear fossula, reserving the term fenestra cochleae for the internal structure bounded by the secondary tympanic membrane. The cochlear fossula is not to be confused with the saccus posticus fossa, which sits directly caudal to the cochlear fossula on the face of the petrosal and contains the saccus posticus of the cavum tympani in life (Fig. 5A) (MacPhee 1981).

### Morphological descriptions

#### Family Oromerycidae

##### Protylopus sp. (SDSNH 40812); Protylopus cf. Pr. stocki (SDSNH 60369)

###### Petrosal

**Fig. 6:**
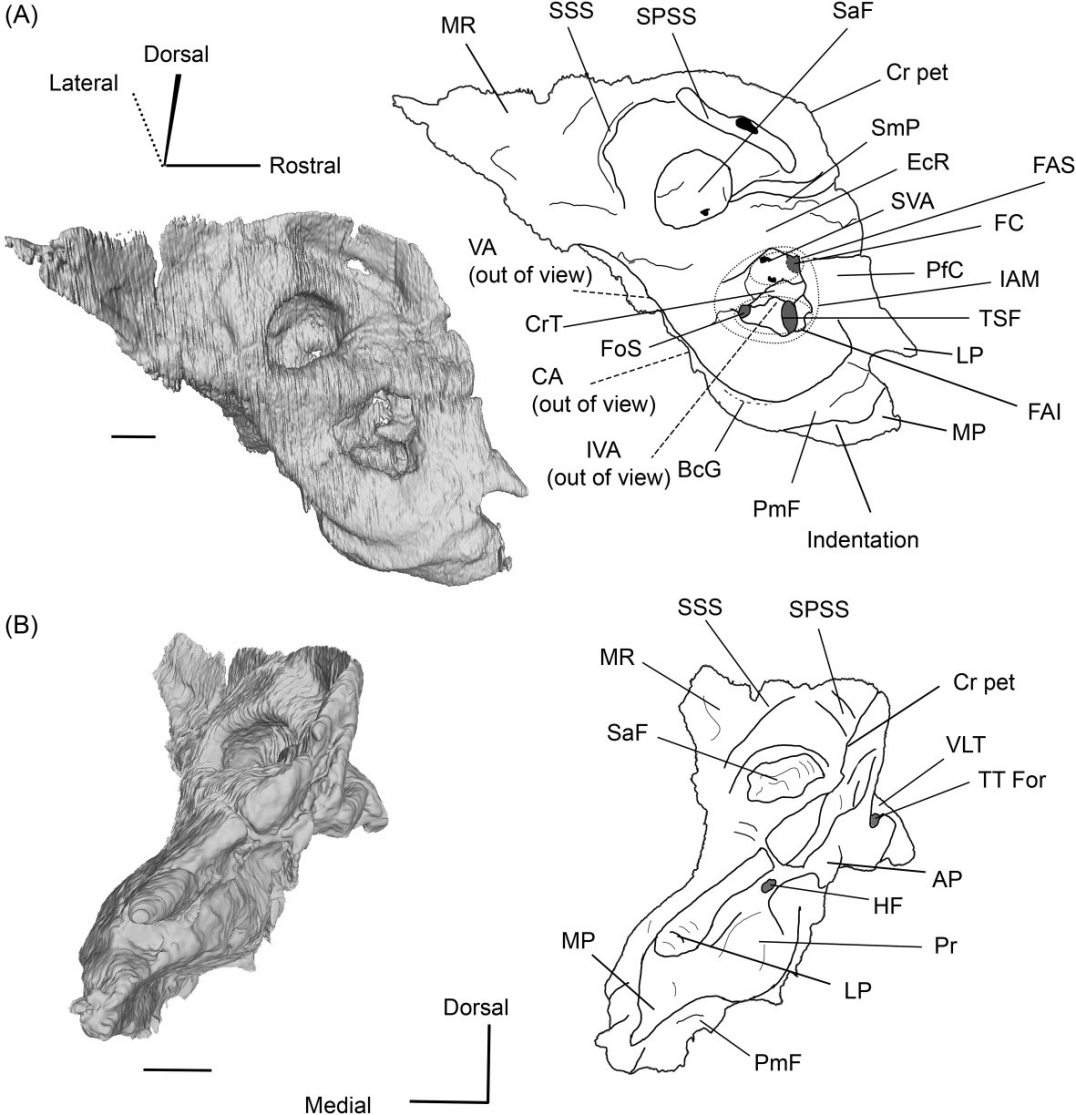
The right (mirrored) petrosal of the oromerycid *Protylopus* cf. *Pr. stocki* (SDSNH 60369) in endocranial **(A)** and rostral **(B)** view. **Abbreviations: AP**, anterior process of the tegmen tympani; **BcG**, basicapsular groove; **CA**, cochlear aqueduct; **CrT**, crista transversa; **Cr pet**, crista petrosa; **EcR**, endocranial ridge; **FAI**, foramen acusticum inferius; **FAS**, foramen acusticum superius; **FC**, facial canal; **FoS**, foramen singulare; **HF**, hiatus Fallopii; **IAM**, internal acoustic meatus; **IAV**, inferior vestibular area; **LP**, lateral process of the epitympanic wing; **MP**, medial process of the epitympanic wing; **MR**, mastoid region; **PfC**, prefacial commissure; **PmF**, posteromedial flange; **Pr**, promontorium; **SaF**, subarcuate fossa; **SmP**, suprameatal plate; **SPSS**, superior petrosal sinus sulcus; **SSS**, sigmoid sinus sulcus; **SVA**, superior vestibular area; **TSF**, tractus spiralis foraminosus; **TT for**, tegmen tympani foramen; **VA**, vestibular aqueduct; **VLT**, ventrolateral tuberosity. Scale bars are 2 mm.

The morphology of the pars cochlearis is not preserved in SDSNH 40812, so the description of this region is solely based on SDSNH 60369. The tympanic face of the *Protylopus* pars cochlearis has an elliptical promontorium (Pr) (Figs. 5A and 6B). While the promontorium does have lateral expansion to accommodate the internal cochlear coils, its rostroventral tip is gently flattened rather than pointed. The epitympanic wing projects anteriorly from the rostroventral border of the promontorium, forming a rounded wedge-like medial process (MP) (Figs. 5A and 6). The lateral process (LP) of the epitympanic wing is a pointed cone, located dorsolateral to the medial process and separated from it by a large “C”-shaped notch corresponding to either the carotid foramen or a confluence between the carotid foramen and piriform fenestra (Figs. 5 and 6) (Orliac and O’Leary 2014). There do not appear to be transpromontorial and stapedial artery sulci, although there is a groove (“Groove” in Fig. 5A) that extends from the muscularis tensor tympani fossa and laterally borders the promontorium. The medial process of the epitympanic wing is confluent with the posteromedial flange (PmF), the latter of which forms a wide ventromedial plate extending from the promontorium caudal to the epitympanic wing (Fig. 5A). The posteromedial flange has a slightly raised ridge along its ventral border (“Ridge” in Fig. 5A) that articulates with the auditory bulla. Caudal to this ridge, there is a small lateral protrusion (“Protrusion” in Fig. 5A) that might be a rostral tympanic process; the protrusion is at the caudal end of the pars cochlearis, as would be expected of the rostral tympanic process, but we cannot determine if it contributes to the tympanic floor of the middle ear (MacPhee 1981; O’Leary 2010). It should be noted that the rostral tympanic process is always a derivative of the pars cochlearis (MacPhee 1981), not the pars canalicularis as was accidentally stated in the definition provided by O’Leary (2010: p. 20–21).

The fenestra vestibuli (FV) and aperture of the cochlear fossula (CF) sit caudal to the promontorium (Fig. 5A). The fenestra vestibuli opens laterally and is oval shaped with a rostrocaudally oriented long axis. Its area ranges from 0.91 mm^2^ to 1.32 mm^2^ (Supplementary Material 4). There is no distinct vestibular fossula surrounding the fenestra vestibuli, but the caudal rim of the fenestra is thick, making the internal length-to-width ratio (1.62 to 1.73) smaller than the external ratio (1.89 to 2.02) (Supplementary Material 4). The fenestra vestibuli is dorsolateral to the cochlear fossula and separated from it by a flat crista interfenestralis (CI) (Fig. 5A). On the right petrosal where the structures are better resolved, the crista interfenestralis is approximately as wide as the fenestra vestibuli (Supplementary Material 4). The aperture of the cochlear fossula is a dorsoventrally elongated oval that opens caudally. There is a very shallow saccus posticus fossa (SPF) directly caudal to the cochlear fossula (Fig. 5A).

The rostral pars canalicularis is also poorly preserved in SDSNH 40812. In SDSNH 60369, the hiatus Fallopii (HF) (the exit for the greater petrosal nerve) is an oval opening on the tegmen tympani (TT) situated dorsal to the promontorium and rostral to the fossa for the tensor tympani muscle (Figs. 5 and 6). The fossa for the muscularis tensor tympani (FMTT) is a circular depression above the promontorium (Fig. 5A). Despite being shallow, it does excavate the tegmen tympani. As mentioned above, a subtle groove extends rostrally from the fossa. Caudal to this fossa, the secondary facial foramen (SFF) opens above the fenestra vestibuli (Fig. 5A). The foramen could not be clearly reconstructed for the right petrosal of SDSNH 60369, but it is clearly present on the left petrosal, and its approximate (Approx.) location is indicated on the right petrosal in Fig. 5A. The secondary facial foramen, and to a lesser extent the fossa for the tensor tympani muscle, are overhung by a broad ventrolateral tuberosity (VLT) (Figs. 5 and 6B). There may be a shallow epitympanic recess (ER), but the morphology in this region is somewhat unclear and there is no indication of a fossa incudis (Fig. 5A). On the left petrosal, there is a bar of bone caudal to the secondary facial foramen that is not indented by the facial sulcus (FS); the facial sulcus originates posterior to this bar, merging with the stapedial muscle fossa (SMF) (Fig. 5A), a structure that is preserved in both *Protylopus* specimens. The fossa is wide and deep and continues caudomedially, terminating at the stylomastoid notch (SMN) (Fig. 5A). The crista parotica (Cr pa) overhangs the stapedial muscle fossa (Fig. 5A). The caudal tympanic process (CTP1) is a small and slightly bulbous projection caudal to the aperture of the cochlear fossula and saccus posticus fossa and medial to the stylomastoid notch (Fig. 5A). This process would be equivalent to the medial portion of the caudal tympanic process described by MacPhee (1981) or cpt1 of Wible and Shelley (2020). Because there is a ridge separating the aperture of the cochlear fossula from the stapedial muscle fossa, it is likely that *Protylopus* also has a lateral portion of the caudal tympanic process equivalent to cpt2 of Wible and Shelley (2020), but the specimens are not preserved well enough to determine if this ridge is continuous with the medial caudal tympanic process. A small portion of the tympanohyal (Th) overhangs the stylomastoid notch (Fig. 5A). The mastoid region (MR) is wedge-shaped with sharp dorsal and lateral ridges (Figs. 5 and 6). The ventromedial part of the mastoid region (visible in endocranial view) is also delimited by a distinct ridge (Fig. 6A). A wedge-shaped mastoid region is common in artiodactyls, but the mastoid region of *Protylopus* is smaller than many others (O’Leary 2010; Orliac and O’Leary 2014; Mennecart et al. 2016; Orliac et al. 2017; Robson et al. 2021; Robson and Theodor 2025).

The dorsolateral face, which could be reconstructed in both specimens, is dominated by the moderately inflated tegmen tympani (TT) (Fig. 5). The body of the tegmen tympani arches dorsally and has a smoothly curving rostral border that intersects with the lateral process of the epitympanic wing (Fig. 5). In SDSNH 60369, a small anterior process (AP) sits just rostral to the ventrolateral tuberosity (Figs. 5A and 6B). The anterior process does not contribute to the visual outline of the petrosal in tympanic view, and it is flush with the tympanic face, but there is a dorsal ridge that defines the process and separates it from the crista petrosa (Cr pet) (Figs. 5B and 6). A reduced tegmen tympani foramen (TTF) is present between the bulbous ventrolateral tuberosity and the anterior process (Figs. 5B and 6B). The rostral portion of the tegmen tympani is a narrow ridge that lacks a tegmen tympani fossa. The anterior process and surrounding area of SDSNH 40812, including the location of the tegmen tympani foramen, were not preserved well enough for description, but it appears that SDSNH 40812 also lacks a tegmen tympani fossa.

**Fig. 7:**
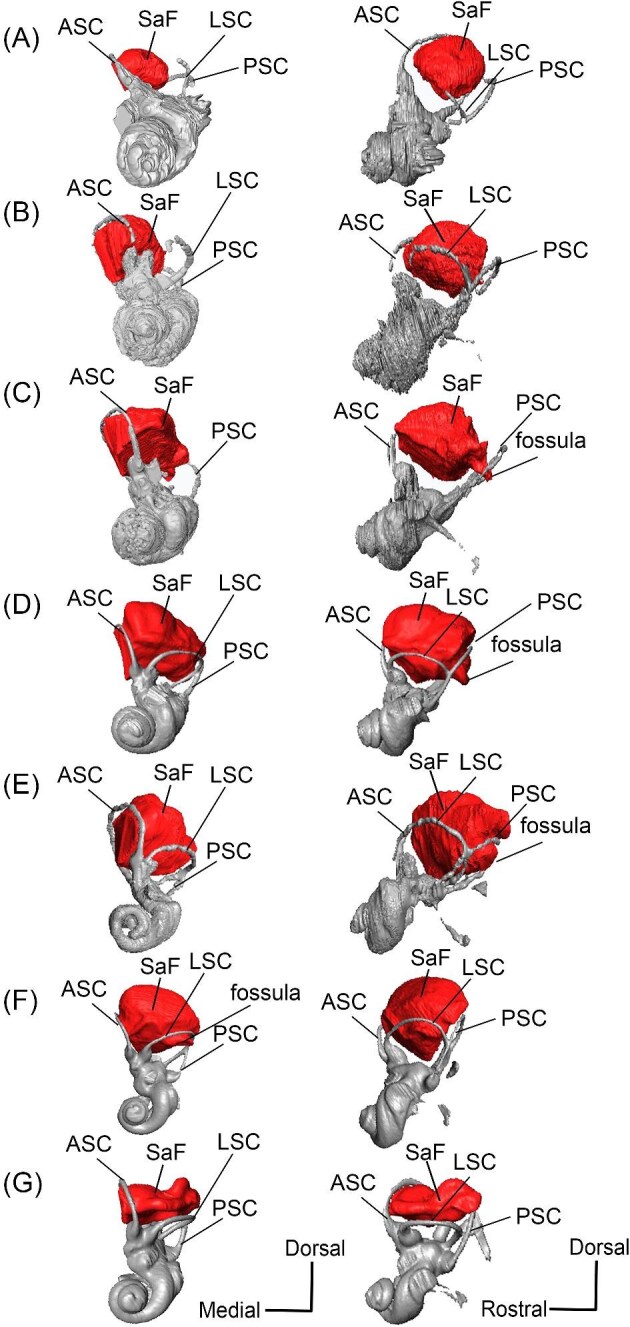
The association between the subarcuate fossa (red) and the bony labyrinth in **A**, *Protylopus* cf. *Pr. stocki* (SDSNH 60369), right (mirrored); **B**, *Eotylopus* cf. *E. reedi* (AMNH FM 47394), right (mirrored); **C**, *Poebrotherium eximium* (AMNH FM 47077), left; **D**, *Poebrotherium wilsoni* (FMNH UM 493), left; **E**, *Paratylopus primaevus* (AMNH FM 9806), left; **F**, *Vicugna vicugna* (UCMZ (M) 1986.309), left; **G**, *Vicugna vicugna* (UCMZ (M) 1986.307), left. Left column is in rostral view. Right column is in tympanic view. **Abbreviations: ASC**, anterior semicircular canal; **LSC**, lateral semicircular canal; **PSC**, posterior semicircular canal; **SaF**, subarcuate fossa. Images not to scale.

A long crista petrosa (Cr pet) extends from above the internal acoustic meatus to the subarcuate fossa (SaF), arching over the endocranial face of the petrosal (Fig. 6). A sulcus, presumably for the superior petrosal sinus (SPSS) (= sinus petrosus dorsalis of Smuts and Bezuidenhout 1987), sits at the base of the tegmen tympani flange, lateral to the subarcuate fossa in both SDSNH 40812 and SDSNH 60369 (Fig. 6). A second sulcus, most likely corresponding to the sigmoid sinus (SSS), is located behind the subarcuate fossa at the caudal end of the posterior semicircular canal (Fig. 6). The subarcuate fossa of SDSNH 60369 is wide and very deep but does not extend through the posterior or lateral semicircular canals (Fig. 7A). While the petrosal of SDSNH 40812 could not be fully reconstructed, the endocranial pars canalicularis was intact; the subarcuate fossa of SDSNH 40812 is much smaller than that of SDSNH 60369, appearing to be a deep cone rather than a very deep sphere (Supplementary Material 3, Fig. S2). The subarcuate fossa of both specimens opens rostromedially, placing it at a different orientation to the dorsomedially directed internal acoustic meatus (Fig. 6A). In SDSNH 40812, a petromastoid canal extends caudally from the posterior end of the subarcuate fossa but cannot be seen in endocranial view.

Based on SDSNH 60369, the subarcuate fossa and the internal acoustic meatus (IAM) are separated by a wide bar of bone that forms an endocranial ridge (EcR) (Fig. 6A). The rostral portion of the ridge flattens out to form a broad suprameatal plate (SmP) (new term) anterior to the subarcuate fossa. The suprameatal plate is not to be confused with the suprameatal bridge of Wible (2003), which is the dorsal boundary of the external acoustic meatus. The area of the prefacial commissure (PfC) rostral and lateral to the internal acoustic meatus is flat and lacks a prefacial commissure fossa (Fig. 6A). There is no anteromedial tuberosity. On the left petrosal, just above the endocranial ridge, there is a deep depression. This depression does not continue onto the surrounding bones and its identity is unclear. The right petrosal has a ridge in this area. The border of the internal acoustic meatus is distinct except along the caudal edge where it is flush with the surrounding bone. The foramen acusticum superius (FAS) is rostrolateral to the foramen acusticum inferius (FAI), separated from it by the crista transversa (CT) (Fig. 6A). The foramen acusticum superius is circular and directed caudomedially while the foramen acusticum inferius is ovate and directed dorsolaterally. The internal aperture of the facial canal (FC), which transmits the greater petrosal nerve in life, is situated rostrolaterally in the foramen acusticum superius (Fig. 6A). Caudally, the superior vestibular area (SVA) is a perforated shallow depression through which part of the vestibular nerve would have passed (Fig. 6A). In the foramen acusticum inferius, the aperture for the cochlear nerve, which traveled through the tractus spiralis foraminosus (TSF), is located rostrally (Fig. 6A). A much smaller foramen singulare (FoS) for a branch of the vestibular nerve is present caudolaterally (Fig. 6A). Just lateral to this, there is a depression corresponding to the inferior vestibular area (IVA) (Fig. 6A).

A shallow basicapsular groove (BcG) is carried on the posteromedial flange, medial to the internal acoustic meatus (Fig. 6A). Because the basicapsular groove is on the endocranial (i.e., dorsomedial) face of the petrosal, it would be coded as having a dorsal position (O’Leary 2010), but it is important to recognize that this coding refers to which face of the petrosal carries the groove, not the position of the groove in relation to other structures. The opening for the cochlear aqueduct (CA) (= cochlear canaliculus) of the perilymphatic duct is a small medially directed slit caudal and lateral to the posteromedial flange (Fig. 6A). The opening for the vestibular aqueduct (VA), which conducts the endolymphatic duct, is a slit on the caudal face located at the posterior end of the endocranial ridge (Fig. 6A). Both openings are slightly obscured in endocranial view. The ventromedial surface of the petrosal is formed by the rounded, wide base of the posteromedial flange (Figs. 5A and 6). The flange is indented medially (“Indentation” in Fig. 6A) but, because of breakage and distortion, we cannot definitively determine whether the indentation is from contact with the basioccipital.

**Fig. 8:**
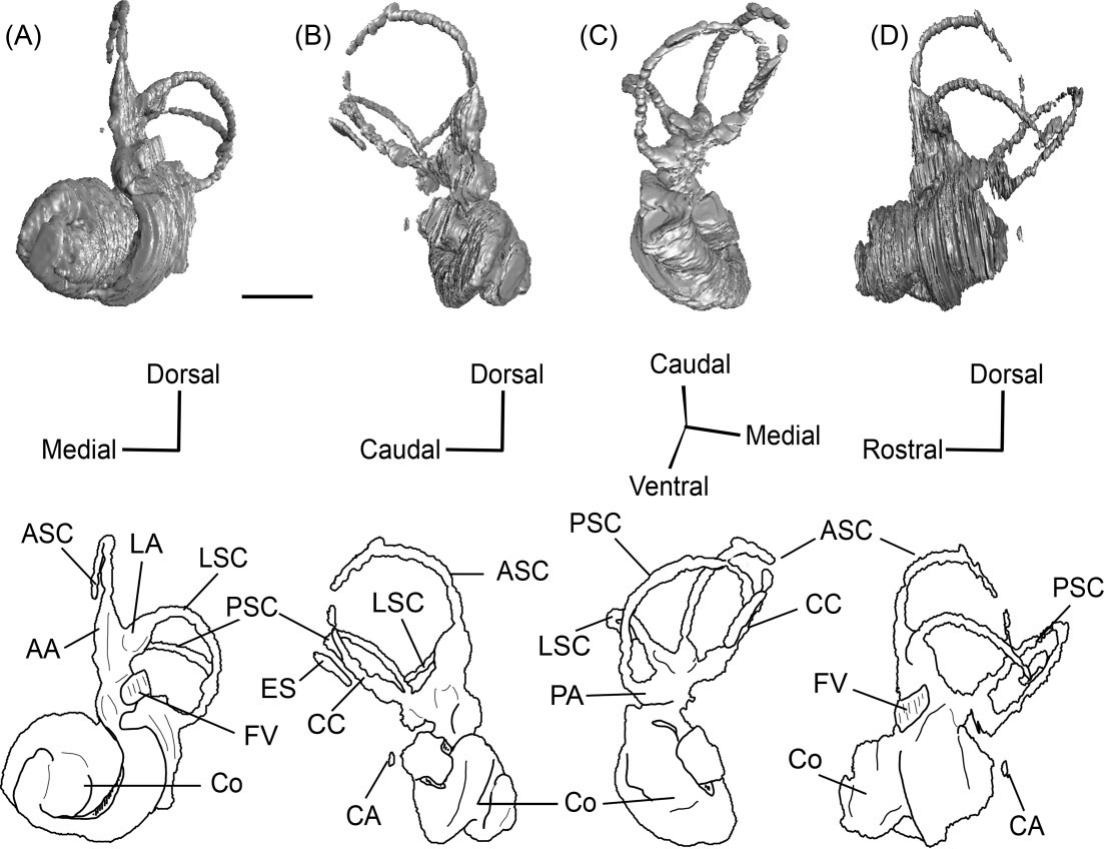
The right (mirrored) bony labyrinth of the oromerycid *Protylopus* cf. *Pr. stocki* (SDSNH 60369) in rostral **(A)**, endocranial **(B)**, ventromedial **(C)**, and tympanic **(D)** view. **Abbreviations: AA**, anterior ampulla; **ASC**, anterior semicircular canal; **CA**, cochlear aqueduct; **CC**, common crus; **Co**, cochlea; **ES**, endolymphatic sac; **FV**, fenestra vestibuli; **LA**, lateral ampulla; **LSC**, lateral semicircular canal; **PA**, posterior ampulla; **PSC**, posterior semicircular canal. Scale bar is 2 mm.

*Bony labyrinth*. The cochlea of *Protylopus* has somewhere between 2.5 to 3 turns, but neither of the specimens are preserved well enough to determine the exact length or number of coils (Fig. 8). It appears to be at least 17 mm long based on rough measurement of SDSNH 60369 (Supplementary Material 4). In both specimens, there is a gap between the basal and second turn, but the apex of the cochlea is tightly wound, contributing to the lack of resolution close to the tip (Fig. 8A). A cochlear aspect ratio could not be calculated for SDSNH 40812, but in SDSNH 60369, the cochlea has an average aspect ratio of 0.56 (Supplementary Material 4). In the left bony labyrinth of SDSNH 40812, for which the outer edge is clearly distinguishable from the surrounding bone, the secondary bony lamina creates a deep groove along the first half of the basal turn of the cochlear endocast. No primary lamina could be observed. The cochlear aqueduct (CA) is a short passage extending from the base of the cochlea that terminates rostral to the arch of the posterior semicircular canal (Fig. 8B, D). The location of its external aperture can be seen in Fig. 6A. Rather than forming a round tube, the internal passage of the cochlear aqueduct is a slit. The internal passage of the vestibular aqueduct could not be reconstructed in either specimen, but a small portion of the endolymphatic sac (ES) was found (Fig. 8B). The recessus sphaericus (associated with the sacculus) and recessus ellipticus (associated with the utriculus) are not clear and cannot be described.

Semicircular canals were reconstructed for both SDSNH 40812 and SDSNH 60369. All three semicircular canals have ampullae, but the amount of inflation could not be determined (Fig. 8). The anterior semicircular canal (ASC) is consistently the largest of the three canals (Supplementary Material 4) and is sigmoidal in shape with the caudal portion of the arch flaring laterally compared to the rostral portion (Fig. 8). The posterior (PSC) and lateral (LSC) semicircular canals are not sigmoid: their arches remain within a single plane (Fig. 8). The lateral semicircular canal is always smaller than the posterior semicircular canal (Supplementary Material 4). The two canals cross paths but remain distinct and do not form a secondary common crus (Fig. 8): the lateral semicircular canal travels medial to the posterior semicircular canal. The common crus (CC) is relatively long, being approximately 70% the height of the anterior semicircular canal (Fig. 8B, C) (Supplementary Material 4). The angles between the three semicircular canals are variable. In SDSNH 40812, the posterior and lateral canals form an angle of much more than 90°, making them the canals with the largest angle, whereas in SDSNH 60369, the posterior/lateral angle is slightly less than 90° and the anterior and posterior canals form the largest angle, being more than 95° (Supplementary Material 4). In both specimens, the anterior and lateral canals form the smallest angle, ranging from 70°–77° (Supplementary Material 4). The angle between the lateral semicircular canal and the basal turn of the cochlea is approximately 16° (Supplementary Material 4). Additional measurement notes can be found in Supplementary Material 3.

#### Family Oromerycidae

##### Eotylopus cf. E. reedi (AMNH FM 47394)

###### Petrosal

**Fig. 9:**
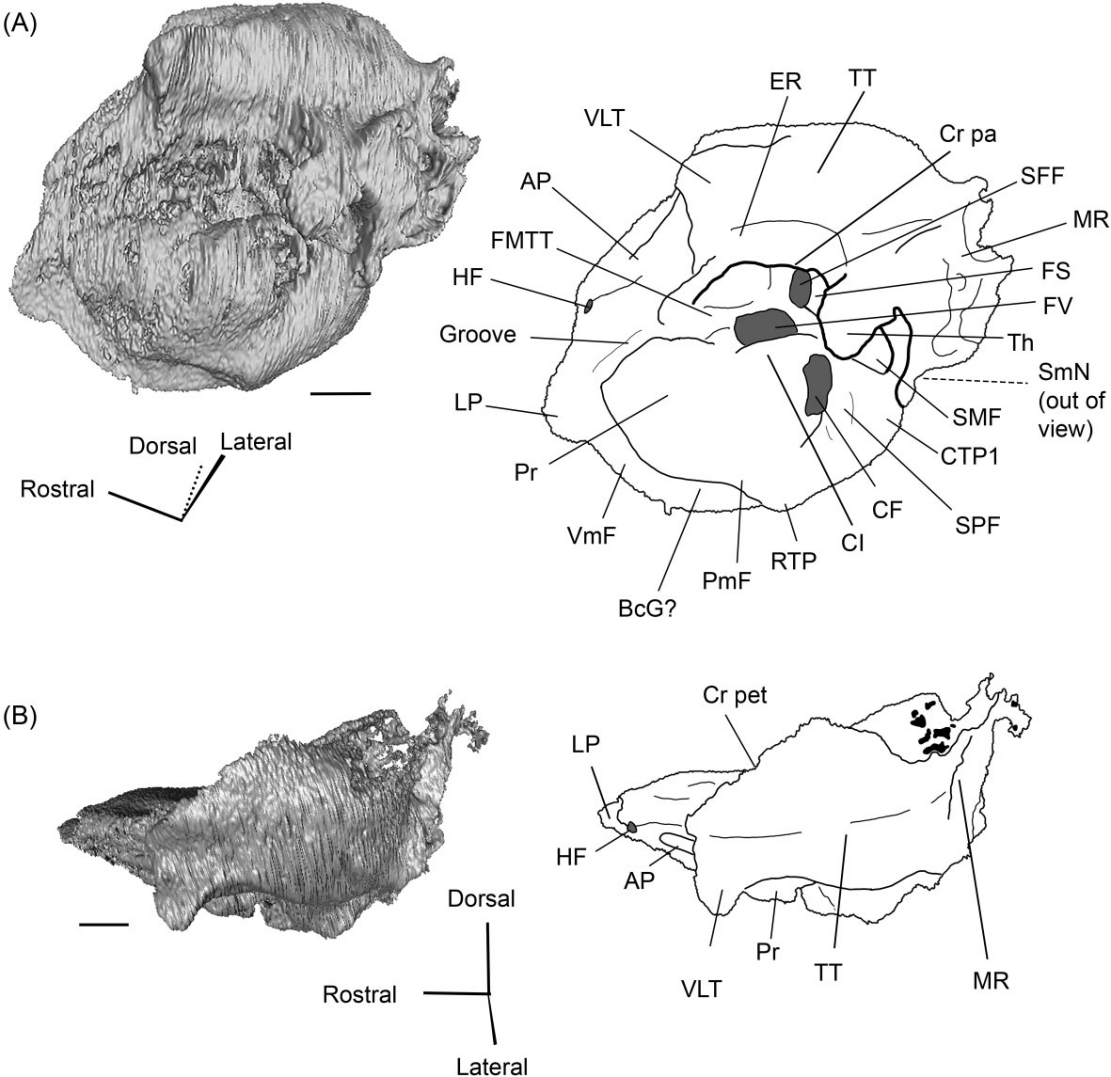
The right (mirrored) petrosal of the oromerycid *Eotylopus* cf. *E. reedi* (AMNH FM 47394) in tympanic **(A)** and dorsolateral **(B)** view. **Abbreviations: AP**, anterior process of the tegmen tympani; **BcG**, basicapsular groove; **CF**, cochlear fossula; **CI**, crista interfenestralis; **CTP1**, medial caudal tympanic process; **Cr pa**, crista parotica; **Cr pet**, crista petrosa; **ER**, epitympanic recess; **FMTT**, fossa for the muscularis tensor tympani; **FS**, facial sulcus; **HF**, hiatus Fallopii; **LP**, lateral process of the epitympanic wing; **MR**, mastoid region; **PmF**, posteromedial flange; **Pr**, promontorium; **RTP**, rostral tympanic process; **SFF**, secondary facial foramen; **SMF**, stapedial muscle fossa; **SmN**, stylomastoid notch; **SPF**, saccus posticus fossa; **Th**, tympanohyal; **TT**, tegmen tympani; **VLT**, ventrolateral tuberosity; **VmF**, ventromedial flange. Scale bars are 2 mm.

**Fig. 10:**
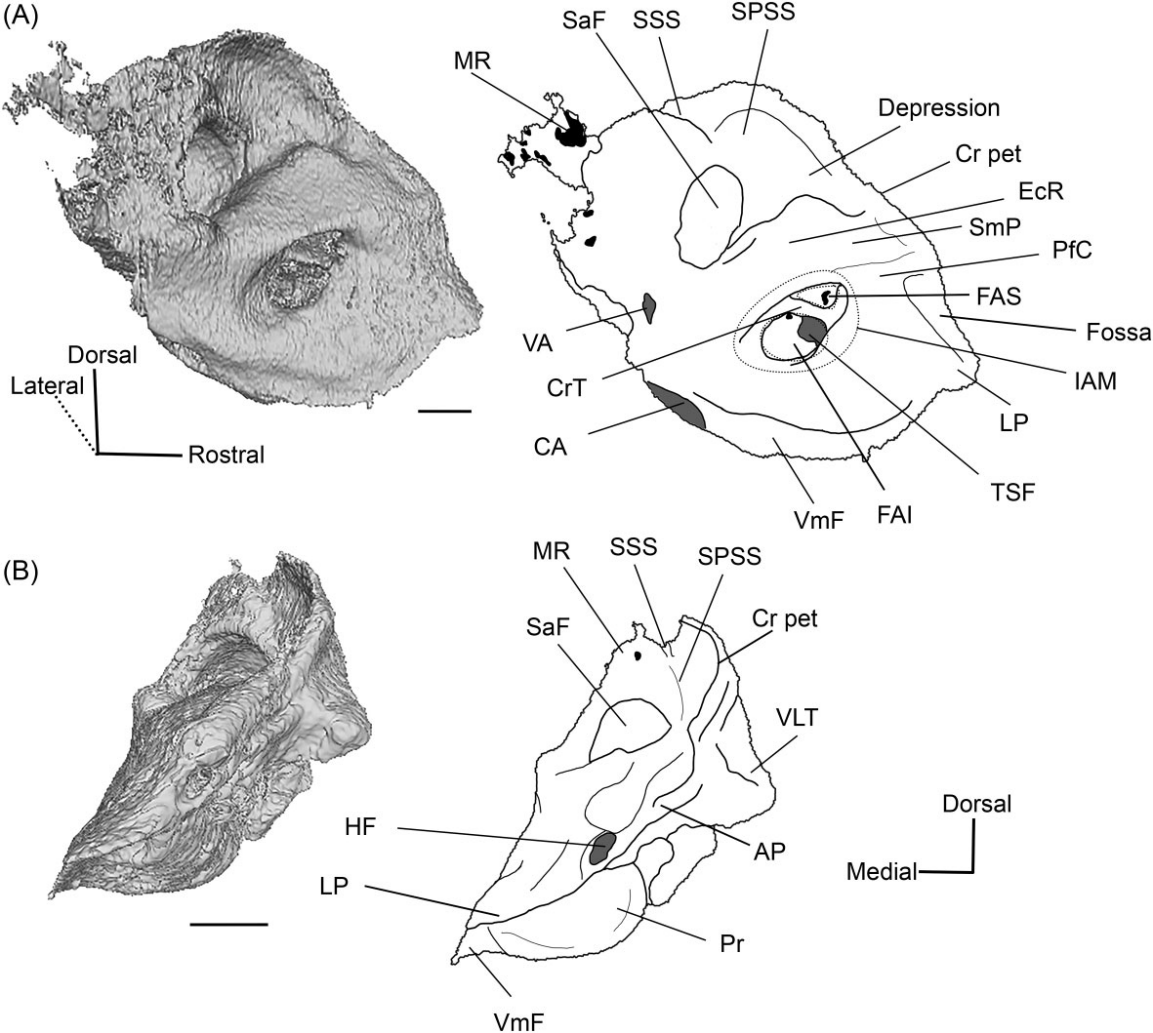
The right (mirrored) petrosal of the oromerycid *Eotylopus* cf. *E. reedi* (AMNH FM 47394) in endocranial **(A)** and rostral **(B)** view. **Abbreviations: AP**, anterior process of the tegmen tympani; **CA**, cochlear aqueduct; **CrT**, crista transversa; **Cr pet**, crista petrosa; **EcR**, endocranial ridge; **FAI**, foramen acusticum inferius; **FAS**, foramen acusticum superius; **HF**, hiatus Fallopii; **IAM**, internal acoustic meatus; **LP**, lateral process of the epitympanic wing; **MR**, mastoid region; **PfC**, prefacial commissure; **Pr**, promontorium; **SaF**, subarcuate fossa; **SmP**, suprameatal plate; **SPSS**, superior petrosal sinus sulcus; **SSS**, sigmoid sinus sulcus; **TSF**, tractus spiralis foraminosus; **VA**, vestibular aqueduct; **VLT**, ventrolateral tuberosity; **VmF**, ventromedial flange. Scale bars are 2 mm.

The promontorium (Pr) of *Eotylopus* is hemi-ellipsoid (teardrop shaped) and domed by the cochlea encapsulated within (Figs. 9 and 10B). There is a single, dorsoventrally flattened process that projects rostral to the promontorium. This process appears to border the carotid foramen caudally and laterally, meaning that it is best identified as the lateral process of the epitympanic wing (LP) (Figs. 9 and 10). The medial process of the epitympanic wing is absent. The lateral process of the epitympanic wing connects to a thin ventromedial flange (VmF) that forms the ventromedial border of the petrosal (Figs. 9A and 10). The posteromedial flange (PmF) is more robust, and the flanges are distinct for most of their length (Fig. 9A). They grade into each other at the level of the fossa for the muscularis tensor tympani (FMTT), forming a slight indentation that deforms the promontorium (Fig. 9A).

The region around the secondary facial foramen (SFF) is broken on the left petrosal and difficult to reconstruct on the right petrosal, but the foramen is located lateral to the fenestra vestibuli (FV) (Fig. 9A). The size and shape are unclear. The fenestra vestibuli is large, oval, and oriented ventrally (Fig. 9A). Like *Protylopus*, there is no distinct vestibular fossula, but the caudal rim of the fenestra vestibuli is thicker than the rostral rim. The internal and external fenestra vestibuli ratios are similar, with the internal ratio being 1.60 and the external ratio being 1.68 (Supplementary Material 4). Based on the right petrosal, the fenestra vestibuli area is 2.60 mm^2^ (Supplementary Material 4). It is separated from the cochlear fossula (CF) by a crista interfenestralis (CI) that is approximately the same width as the fenestra vestibuli (Fig. 9A) (Supplementary Material 4). The cochlear fossula is also large, but it is circular and directed caudally (Fig. 9A). A shallow saccus posticus fossa (SPF) extends caudomedially from its aperture (Fig. 9A). There is a knob-like rostral tympanic process (RTP) ventromedial to the fenestra cochleae (Fig. 9A). This process articulates with the basisphenoid and defines the rostral border of the jugular foramen.

The ventromedial face of the petrosal is composed of the body of the posteromedial flange and the lateral side of the ventromedial flange (Fig. 9A). Anterior to the rostral tympanic process, which comes into contact with the basioccipital, the ventromedial flange seemingly carries the basicapsular groove (BcG), reflecting the petrosal contribution to the petrobasilar canal (Fig. 9A). The basicapsular groove is typically associated with the endocranial (i.e., dorsomedial) face rather than the tympanic face, but it is on the tympanic (i.e., ventrolateral) or ventromedial face of anthracotheriids and hippopotamids (O’Leary 2010; Orliac et al. 2013, 2023). Whether the morphology of *Eotylopus* constitutes a true groove may be debatable given that there is no raised rim (see description of *V. vicugna* for additional discussion), but even if no groove is present, it appears that the location of the petrobasilar canal was laterally shifted.

A groove (“Groove” in Fig. 9A) runs along the dorsolateral border of the promontorium. It intersects with the fossa for the muscularis tensor tympani (FMTT) which, though incompletely reconstructed, appears to be circular and deep (Fig. 9A). The epitympanic recess (ER) is shallow but long and it indents the tegmen tympani caudal to the muscularis tensor tympani fossa (Fig. 9A). There is no fossa incudis. Caudal to the secondary facial foramen, the facial sulcus (FS) forms a wide channel that is roofed by the crista parotica (Cr pa) (Fig. 9A). The stapedial muscle fossa (SMF) extends from the fenestra vestibuli and passes medial to the facial sulcus before the two overlap and create a large sulcus that terminates at the stylomastoid notch (SmN) (Fig. 9A). The right tympanohyal (Th) is present—it forms an arch that rostrally borders the notch (Fig. 9A). The medial caudal tympanic process (CTP1) sits directly behind the saccus posticus fossa and is smoothly rounded (Fig. 9A). Based on morphology and location, it fulfils the criteria for a caudal tympanic process widely used in the artiodactyl literature (O’Leary 2010), but the process is not a distinct ridge or knob. It is unclear whether a lateral portion of the caudal tympanic process is present. The mastoid region (MR) could not be fully reconstructed but appears to have had little caudal expansion (Figs. 9 and 10).

The tegmen tympani (TT) is moderately inflated and divided into two sections separated by a step (Fig. 9). The more ventrolateral portion of the tegmen tympani is rounded caudally but indented rostrally by the epitympanic recess (ER) (Fig. 9A). There is a large and blunt ventrolateral tuberosity (VLT) (Figs. 9 and 10B). The more dorsal portion of the tegmen tympani is a curved plate that extends slightly over the endocranial face of the petrosal and carries a long crista petrosa (Cr pet) (Figs. 9B and 10). The tegmen tympani lacks a tegmen tympani foramen and tegmen tympani fossa. The anterior process (AP) of the tegmen tympani is small and not expanded enough to contribute to the border of the petrosal or extend beyond the start of the epitympanic wing (Figs. 9 and 10B). It is flush with the tympanic surface, although it has a dorsolateral ridge that makes it distinct from the rest of the tegmen tympani. The hiatus Fallopii (HF) is an oval opening that pierces the rostrolateral edge of the tegmen tympani adjacent to the anterior process (Figs. 9 and 10B).

The endocranial face of the petrosal is occupied by a large internal acoustic meatus (IAM) and subarcuate fossa (SaF) (Fig. 10). The foramen acusticum superius (FAS) is dorsolateral to the foramen acusticum inferius (FAI), but the two are not greatly rostrocaudally offset from each other and they are separated by a narrow crista transversa (CrT) (Fig. 10A). The aperture for the facial canal is nestled within the rostrolateral area of the foramen acusticum superius, but it is not figured because it is obscured in endocranial view. A larger opening leading to the canal and cribriform tract of the cochlear nerve (i.e., tractus spiralis foraminosus) (TSF) is present rostrally in the foramen acusticum inferius (Fig. 10A). The foramen singulare and the superior and inferior vestibular areas could not be located. The internal acoustic meatus is bordered by a ridge for most of its circumference. This is particularly pronounced caudally where the ridge overhangs the foramen acusticum superius. However, the ridge is absent ventromedially, leaving the internal acoustic meatus flush with the surrounding area. The area around the internal acoustic meatus is generally flat, but there is a small step at the ventral border that corresponds to the medial edge of the ventromedial flange (VmF) (Fig. 10A). The cochlear aqueduct (CA) is located at the caudal end of this step and is visible in endocranial view (Fig. 10A).

Rostrolaterally, there is also a shallow fossa (“Fossa” in Fig. 10A) at the level of the hiatus Fallopii at the termination of the crista petrosa (Cr pet). This fossa may correspond to the trigeminal sulcus (Fig. 10A). The prefacial commissure (PfC) is located just caudal to this fossa (Fig. 10A). There is no anteromedial tuberosity or prefacial commissure fossa. The internal acoustic meatus and the subarcuate fossa are separated by a wide and flat endocranial ridge (EcR) that forms a large suprameatal plate (SmP) rostrally (Fig. 10A). A deep depression (“Depression” in Fig. 10A) sits just lateral to the subarcuate fossa. The vestibular aqueduct (VA) is a slit posterior to the ridge (Fig. 10A). There is a faint sulcus extending caudally from the depression that probably corresponds to the superior petrosal sinus (SPSS) (Fig. 10). Based on the left petrosal, a sigmoid sinus sulcus (SSS) travels along the mastoid region caudal to the subarcuate fossa (location inferred in Fig. 10). The subarcuate fossa (SaF) opens almost entirely rostrally; in endocranial view, the aperture is partially blocked by the endocranial ridge (Fig. 10). The fossa is very deep and subspherical (Fig. 7B). No part of the fossa extends through the arches of the posterior and semicircular canals, but the fossa does extend above the arch of the anterior semicircular canal (Fig. 7B). The petromastoid canal is at the caudal end of the subarcuate fossa but its exit cannot be located externally. There is another canal associated with the subarcuate fossa, present bilaterally. It begins in the caudolateral wall of the fossa, ventral to the petromastoid canal and exits the petrosal in the mastoid region, rostroventral to the petromastoid canal. This canal likely also conducted a soft tissue structure associated with the subarcuate fossa.

**Fig. 11:**
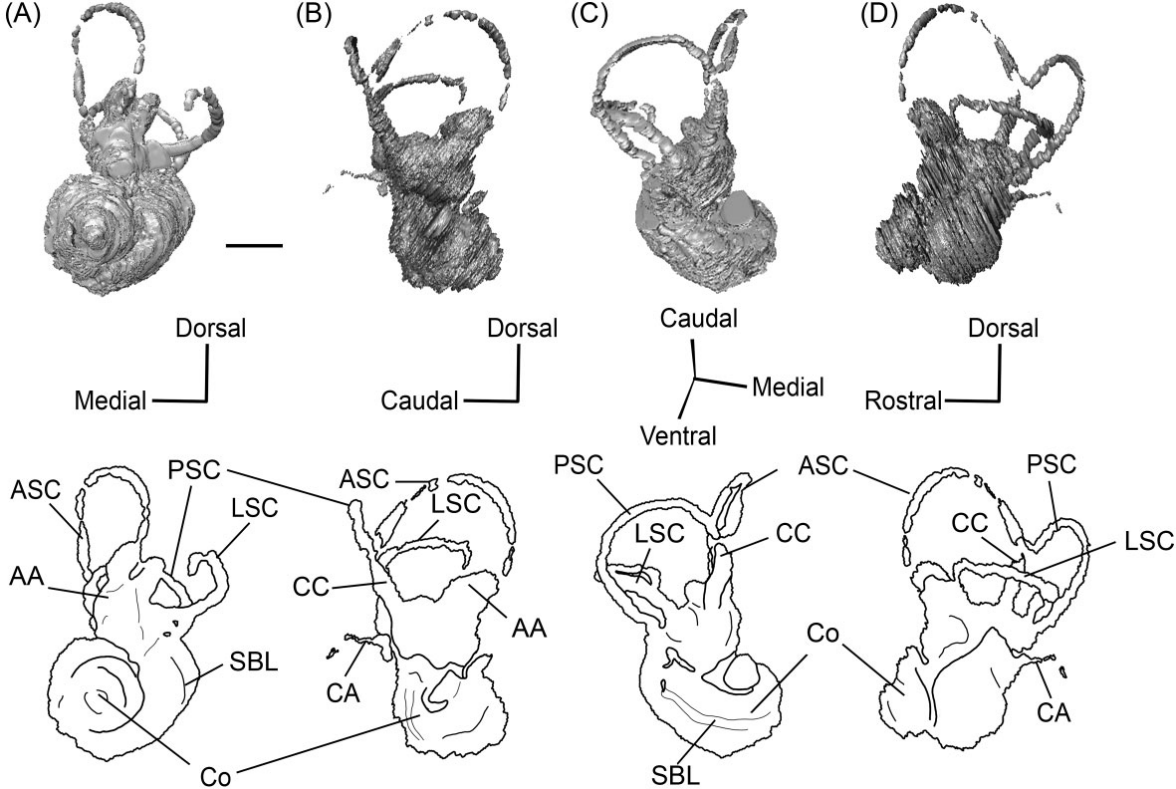
The right (mirrored) bony labyrinth of the oromerycid *Eotylopus* cf. *E. reedi* (AMNH FM 47394) in rostral **(A)**, endocranial **(B)**, ventrolateral **(C)**, and tympanic **(D)** view. Abbreviations: **AA**, anterior ampulla; **ASC**, anterior semicircular canal; **CA**, cochlear aqueduct; **CC**, common crus; **Co**, cochlea; **LSC**, lateral semicircular canal; **PSC**, posterior semicircular canal; **SBL**, secondary bony lamina. Scale bar is 2 mm.

*Bony labyrinth.* The cochlea is not well preserved. Its gross morphology appears to be like that of *Protylopus* with a gap between the basal and second coil and somewhere between 2–3 turns (Fig. 11). It is at least 17.5 mm long and the aspect ratio is approximately 0.72 (Supplementary Material 4). The secondary bony lamina (SBL) is expressed as a wide groove on at least the first half of the basal turn of the cochlea (its presence cannot be determined past this point) (Fig. 11A, C). The primary bony lamina could not be identified. The cochlear aqueduct (CA) is relatively long and narrow, extending past the arc of the posterior semicircular canal (PSC) (Fig. 11B, D). The vestibular aqueduct and endolymphatic sac could not be located. The anterior and lateral ampullae (AA; LA) and portions of all three semicircular canals could be reconstructed; the anterior and posterior canals are mostly intact, while only the caudal part of the lateral semicircular canal (LSC) is present (Fig. 11). The anterior semicircular canal (ASC) is larger than the posterior semicircular canal (PSC) and looks to have been larger than the lateral semicircular canal (Fig. 11) (Supplementary Material 4). Its arch is sigmoidal. The posterior semicircular canal has a slight rostral inflection at the apex of its arch, but this may be due to taphonomy. The angle between the anterior and posterior semicircular canals is less than 90° (Supplementary Material 4), and they merge into a typical common crus (CC) that is approximately 60% the height of the anterior semicircular canal (Fig. 11) (Supplementary Material 4). The shape of the lateral semicircular canal could not be fully determined. The lateral semicircular canal passes medial to the posterior semicircular canal—they do not form a secondary common crus (Fig. 11). Based on the portion of the lateral semicircular canal that is present, the posterior and lateral semicircular canals are at an angle of approximately 98°, and the anterior and lateral semicircular canals are at an angle of approximately 81° (Supplementary Material 4). The lateral semicircular canal forms an approximately 32° angle with the basal turn of the cochlea (Supplementary Material 4).

#### Family Camelidae

##### Poebrotherium eximium (AMNH FM 42298; AMNH FM 47077); Poebrotherium wilsoni (FMNH UM 493; FMNH UM 465); Poebrotherium sp. (AMNH FM 147015)

###### Petrosal

The petrosal morphology of *Poebrotherium* has been described by Whitmore (1953) based on cranial serial sections, and by O’Leary (2010) based on the morphology of a petrosal dissected out of AMNH FM 140343. The specimen described by Whitmore (1953) was referred to *P. wilsoni*, and the specimen described by O’Leary (2010) was not identified to species level. We expand on these descriptions with additional specimens referred to *Poebrotherium* sp. and *Po. wilsoni*, and we add a description of *Po. eximium*, along with documenting ontogenetic changes.

**Fig. 12:**
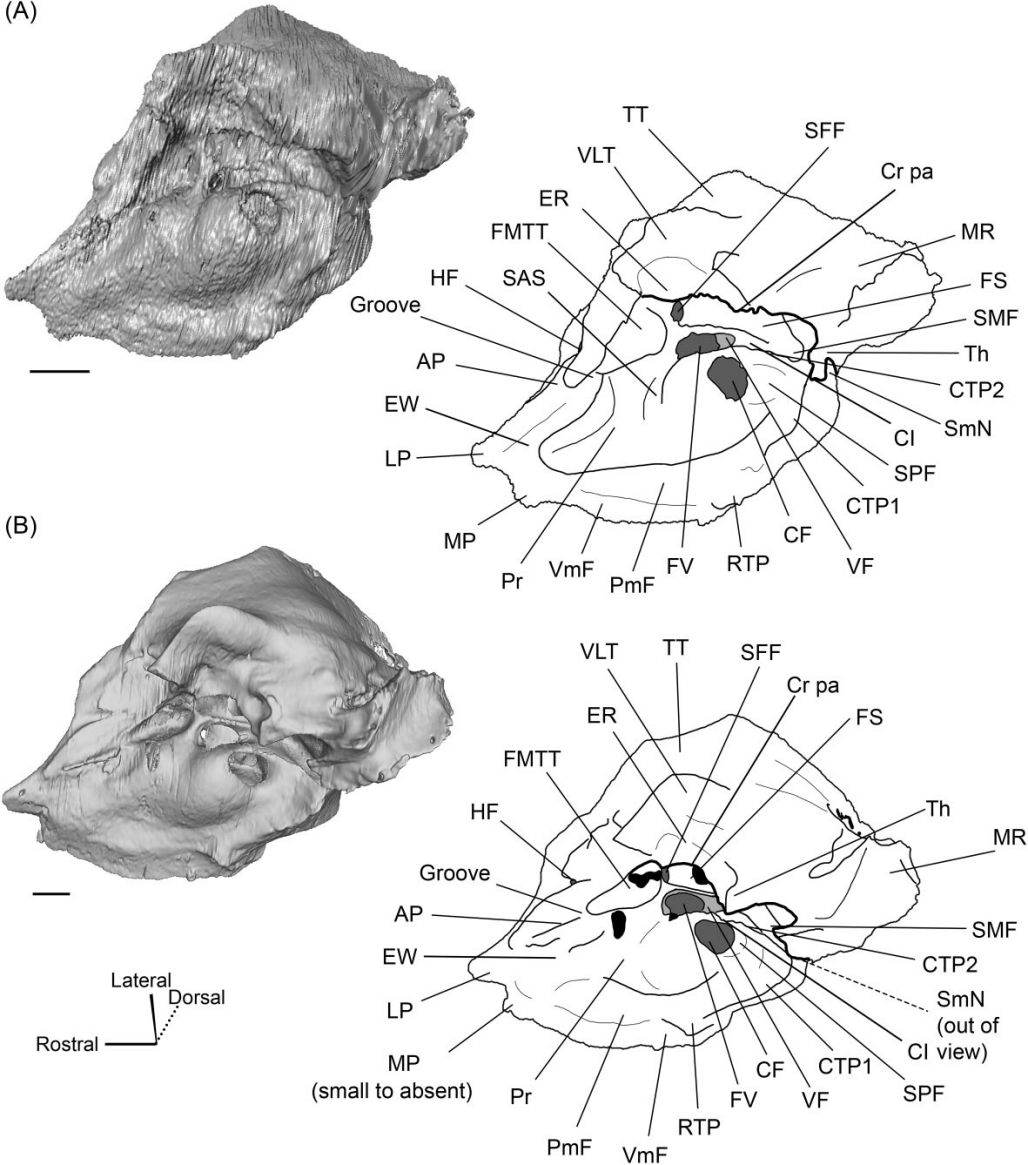
The left petrosal of the camelids *Poebrotherium eximium* (AMNH FM 42298) (**A**) and *Poebrotherium wilsoni* (FMNH UM 493) (**B**) in tympanic view. **Abbreviations: AP**, anterior process of the tegmen tympani; **CF**, cochlear fossula; **CI**, crista interfenestralis; **CTP1**, medial caudal tympanic process; **CTP2**, lateral caudal tympanic process; **Cr pa**, crista parotica; **ER**, epitympanic recess; **EW**, epitympanic wing; **FMTT**, fossa for the muscularis tensor tympani; **FS**, facial sulcus; **FV**, fenestra vestibuli; **HF**, hiatus Fallopii; **LP**, lateral process of the epitympanic wing; **MP**, medial process of the epitympanic wing; **MR**, mastoid region; **PmF**, posteromedial flange; **Pr**, promontorium; **RTP**, rostral tympanic process; **SAS**, stapedial artery sulcus; **SFF**, secondary facial foramen; **SMF**, stapedial muscle fossa; **SmN**, stylomastoid notch; **SPF**, saccus posticus fossa; **Th**, tympanohyal; **TT**, tegmen tympani; **VF**, vestibular fossula; **VLT**, ventrolateral tuberosity; **VmF**, ventromedial flange. Scale bars are 2 mm.

**Fig. 13:**
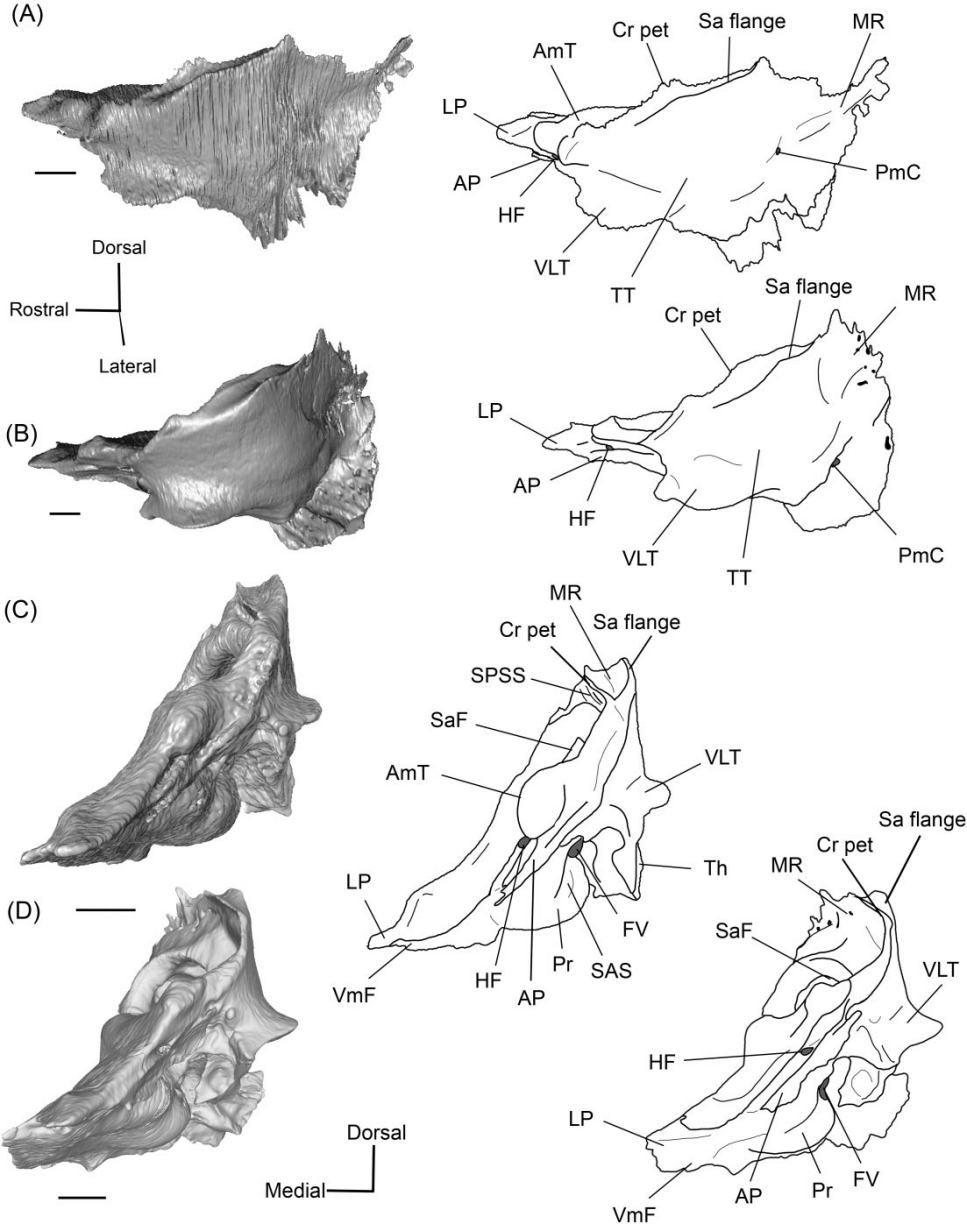
The left petrosal of the camelids *Poebrotherium eximium* (AMNH FM 42298) **(A, C)** and *Poebrotherium wilsoni* (FMNH UM 493) **(B, D)** in dorsolateral **(A, B)** and rostral view **(C, D)**. Axes of orientation are shared in each view. **Abbreviations: AmT**, anteromedial tuberosity; **AP**, anterior process of the tegmen tympani; **Cr pet**, crista petrosa; **FV**, fenestra vestibuli; **HF**, hiatus Fallopii; **LP**, lateral process of the epitympanic wing; **MR**, mastoid region; **PmC**, petromastoid canal; **Pr**, promontorium; **Sa flange**, subarcuate flange; **SaF**, subarcuate fossa; **SAS**, stapedial artery sulcus; **SPSS**, superior petrosal sinus sulcus; **Th**, tympanohyal; **TT**, tegmen tympani; **VLT**, ventrolateral tuberosity; **VmF**, ventromedial flange. Scale bars are 2 mm.

**Fig. 14:**
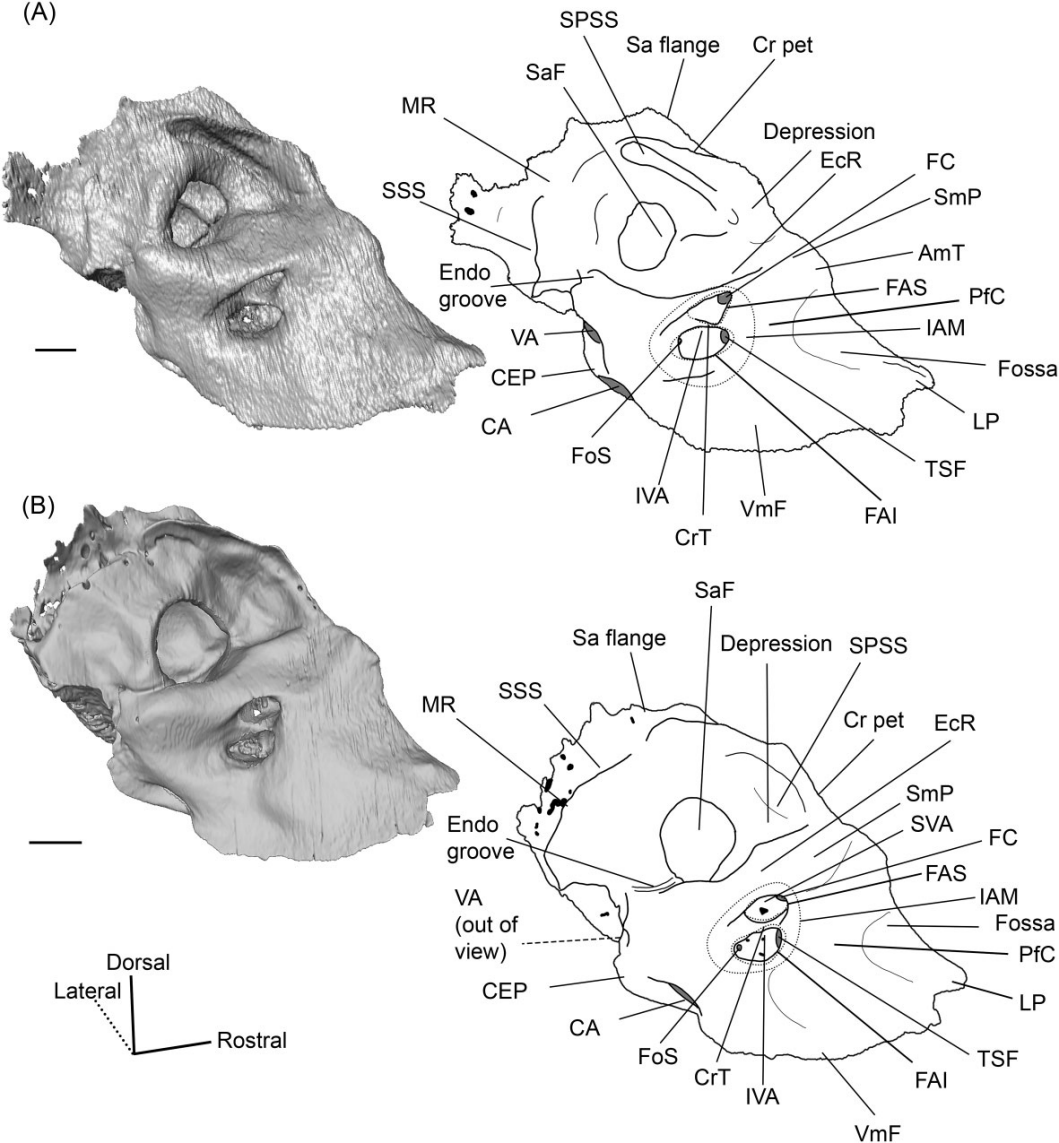
The left petrosal of the camelids *Poebrotherium eximium* (AMNH FM 42298) (**A**) and *Poebrotherium wilsoni* (FMNH UM 493) (**B**) in endocranial view. **Abbreviations: AmT**, anteromedial tuberosity; **CA**, cochlear aqueduct; **CEP**, caudal endocranial process; **CrT**, crista transversa; **Cr pet**, crista petrosa; **EcR**, endocranial ridge; **FAI**, foramen acusticum inferius; **FAS**, foramen acusticum superius; **FC**, facial canal; **FoS**, foramen singulare; **IAM**, internal acoustic meatus; **IVA**, inferior vestibular area; **LP**, lateral process of the epitympanic wing; **MR**, mastoid region; **PfC**, prefacial commissure; **Sa flange**, subarcuate flange; **SaF**, subarcuate fossa; **SmP**, suprameatal plate; **SPSS**, superior petrosal sinus sulcus; **SSS**, sigmoid sinus sulcus; **SVA**, superior vestibular area; **TSF**, tractus spiralis foraminosus; **VA**, vestibular aqueduct; **VmF**, ventromedial flange. Scale bars are 2 mm.

The morphology of the petrosal does not appear to greatly differ between *Po. wilsoni* and *Po. eximium* (Figs. 12–14). It is a wedge-shaped bone, a morphology that is caused in large part because of the epitympanic wing (EW) and posteromedial and ventromedial flanges (Fig. 12). As noted by O’Leary (2010), the epitympanic wing is a sizable flat expanse rostral to the promontorium (Pr) (Fig. 12). The medial process of the epitympanic wing (MP) is greatly reduced and sometimes absent (e.g., AMNH FM 47077) (Fig. 12). The lateral process of the epitympanic wing (LP) is a distinct triangular projection that terminates at a sharp point (Figs. 12–14). There is sometimes a C-shaped notch medial to the lateral process that corresponds to the caudal border of the carotid foramen (Fig. 12). The posteromedial flange (PmF) is confluent with the ventromedial flange (VmF), which in turn forms a large keel below the promontorium (Fig. 12). The change in orientation between the posteromedial flange and ventromedial flange is apparent in some (e.g., AMNH FM 42298; FMNH UM 493) (Fig. 12) but not all (e.g., AMNH FM 147015) specimens; this seems to be individual variation rather than a phylogenetic or ontogenetic effect. A rostral tympanic process (RTP) consistently projects from the ventral border of the posteromedial flange (Fig. 12) and can be used to distinguish the posteromedial flange from the ventromedial flange when the two are indistinct.

The presence of a rostral tympanic process that projects from the ventral surface of the pars cochlearis and contributes to the bony floor of the tympanic cavity (MacPhee 1981; O’Leary 2010) was not noted by Whitmore (1953) or O’Leary (2010). Examination of AMNH FM 140343, the specimen described by O’Leary (2010), reveals that there is a rostral tympanic process and, with the petrosal in situ and the auditory bulla articulated, the process contributes to the bony floor of the tympanic cavity (Supplementary Material 3, Fig. S3). However, the contact between the process and the bulla is not complete (Supplementary Material 3, Fig. S3). When the petrosal is not in situ or the contact with adjacent bones cannot be determined, the rostral tympanic process of *Poebrotherium* can be identified primarily based on location: it sits ventromedial to the promontorium and slightly rostral to the aperture of the cochlear fossula (CF), adjacent to the ventromedial flange (Fig. 12). Although it may contact the caudal tympanic process (CTP) of the pars canalicularis, the rostral tympanic process always projects more ventromedially (Fig. 12). O’Leary (2010) described a groove on AMNH FM 140343 which is in the vicinity of what we identify as the rostral tympanic process. We did not observe such a groove on other *Poebrotherium* specimens, and it is likely a product of individual variation.

The promontorium (Pr) is hemi-ellipsoid (O’Leary 2010) with the rostral portion flatter and narrower than the expanded caudal portion (Figs. 12 and 13B–C). There is variably a shallow stapedial artery sulcus (SAS) descending from the fenestra vestibuli (Fig. 12A), and while there is no transpromontorial sulcus, there is often a groove (“Groove” in Fig. 12) extending rostrally from the fossa for the muscularis tensor tympani (FMTT). O’Leary (2010) described the promontorium as having two ovoid bulges; based on our reconstruction of the bony labyrinth, these correspond to the basal and second coils of the cochlea. The convexity of the promontorium is most pronounced in the region of the vestibular and cochlear fossulae where it covers the basal coil. The fenestra vestibuli (FV) and vestibular fossula (VF) of *Poebrotherium* are distinct structures, with the former recessed in the latter (Fig. 12). Both structures have a rostrocaudal long axis and open ventrolaterally, but unlike the oval fenestra vestibuli, most of the anteroposterior expansion of the vestibular fossula occurs caudally, resulting in a tear-drop shape. The fenestra vestibuli has a length-to-width ratio ranging from 1.31 to 2.23, while the vestibular fossula has a ratio of 1.86 to 3.31 (Supplementary Material 4). Specimens of *Poebrotherium* have a fenestra vestibuli area of 1.42 mm^2^ to 2.44 mm^2^, and the two measured species overlap in size, although *Po. wilsoni* is on average slightly larger: *Po. eximium* ranges from 1.42 mm^2^ to 1.93 mm^2^, while *Po. wilsoni* ranges from 1.81 mm^2^ to 2.44 mm^2^ (Supplementary Material 4). Two of the two juveniles that could be measured (AMNH FM 147015; FMNH UM 493) are at the high end of this range, while the third (AMNH FM 42298) has the smallest area. One specimen of *Poebrotherium* sp. (AMNH FM 147015) has the largest area, while the other specimen (FMNH PM 14560) has the second-smallest area (Supplementary Material 4). The secondary facial foramen (SFF) sits dorsolateral to the fenestra vestibuli (Fig. 12) (O’Leary 2010). It is directed caudally and is either subcircular or ovate. The crista interfenestralis (CI) is typically slightly narrower than the vestibular fossula, but in FMNH UM 456, it is slightly wider (Fig. 12) (Supplementary Material 4). This crista interfenestralis separates the vestibular fossula from the aperture of the cochlear fossula (CF), which is a large opening that is either circular (Fig. 12A) or oval with a rostrocaudal long axis (Fig. 12B). O’Leary (2010) considered the aperture (identified as the fenestra cochleae by O’Leary) to be circular, but the oval morphology is more common in the specimens we examined—AMNH FM 42298 is the only specimen that has a distinctly circular aperture. A wide saccus posticus fossa (SPF) extends caudally from the aperture of the cochlear fossula (Fig. 12).

The fossa for the muscularis tensor tympani (FMTT) is shallow and the caudal portion is subcircular (Fig. 12). The bone in this area is very thin and sometimes eroded to expose the internal passage of the greater petrosal nerve, but this is likely an artifact of preservation. In some specimens, the rostral portion of the fossa is greatly elongated, giving the fossa a triangular shape (e.g., FMNH UM 493) (Fig. 12B). It is separated from the secondary facial foramen by a narrow crista. We concur with O’Leary (2010) that the fossa does not excavate the tegmen tympani. The facial sulcus (FS) is a broad, open channel extending caudally from the secondary facial foramen and overhung by a distinct crista parotica (Cr pa) (Fig. 12). In the area of the stylomastoid notch (SmN), the stapedial muscle fossa (SMF) forms a deep subcircular or oval indentation (O’Leary 2010), increasing the apparent size of the notch (Fig. 12). In many specimens, a portion of the tympanohyal (Th) is preserved as a small ventral projection extending from the crista parotica and mastoid region (MR) (Figs. 12 and 13C). When present, the tympanohyal encloses the stylomastoid notch (SmN), forming the stylomastoid foramen (Fig. 12). The facial sulcus (FS) travels through the stylomastoid notch/foramen and wraps ventromedially around the caudal portion of the petrosal (Fig. 12). A wedge-like medial caudal tympanic process (CTP1) rostrally borders the facial sulcus just ventral to the stylomastoid notch and caudal to the saccus posticus fossa (Fig. 12). There is also a clear ridge that extends from the crista interfenestralis to laterally border the saccus posticus fossa and the aperture of the cochlear fossula—this would correspond to the lateral portion of the caudal tympanic process (CTP2) (Fig. 12) (MacPhee 1981). O’Leary (2010) did not distinguish between the medial and lateral portions of the process but did observe that the crista interfenestralis was continuous with the caudal tympanic process. O’Leary (2010) also noted that the continued groove of the facial sulcus separates the medial caudal tympanic process from the mastoid region. When intact, the mastoid region (MR) is a small fan that does not greatly extend caudal to the rest of the petrosal (Figs. 12-14) (O’Leary 2010).

The tegmen tympani (TT) is moderately inflated with a shelf- or knob-like ventrolateral tuberosity (VLT) (Figs. 12 and 13). The shape of the ventrolateral tuberosity is most likely a result of individual variation as there are no consistent differences between ontogenetic stages or species. Regardless of shape, all specimens of *Poebrotherium* have a large ventrolateral tuberosity.

The epitympanic recess (ER) is a shallow depression nestled under the ventrolateral tuberosity, separated from the facial sulcus by the crista parotica (Fig. 12) (O’Leary 2010). The fossa incudis is absent. Rostral to the ventrolateral tuberosity, there is small anterior process (AP) (O’Leary 2010) that frequently has a flattened dorsolateral face and a dorsolateral ridge (Figs. 12 and 13). The process is flush with the tympanic face of the petrosal and does not contribute to the outline of the bone. The tegmen tympani anteriorly narrows into a blade that intersects with the lateral process of the epitympanic wing (Figs. 12 and 13). There is no tegmen tympani foramen or tegmen tympani fossa. The hiatus Fallopii (HF) is a small rostrally-directed oval that sits on the lateral portion of the tegmen tympani, separated from the promontorium by the anterior process (Figs. 12 and 13). A hiatus Fallopii sulcus extends rostrally from the opening.

The endocranial pars canalicularis is dorsolaterally bordered by two ridges. It appears that the more lateral ridge is the subarcuate flange (Sa flange) (see Mourlam 2019) and the more medial ridge is the crista petrosa (Cr pet), but it may be that the crista petrosa of *Poebrotherium* grades into what Mourlam (2019) identified as the subarcuate flange, leaving the more lateral structure unnamed (Figs. 13 and 14). For the purpose of this description, we restrict “crista petrosa” to the medial ridge bounding the subarcuate fossa and reserve “subarcuate flange” for the lateral ridge sensu Mourlam (2019). When the two cannot be distinguished, we preferentially refer to the entire structure as the crista petrosa sensu lato.

**Fig. 15:**
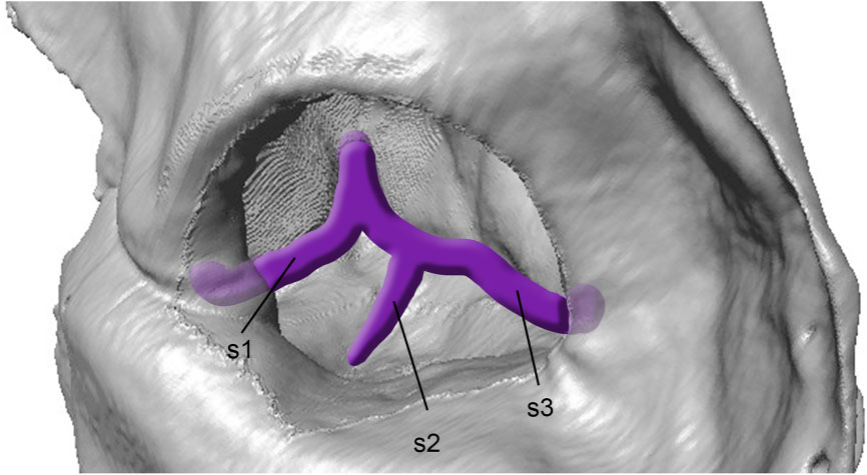
The left subarcuate fossa of *Poebrotherium wilsoni* (FMNH UC 493) showing the three sulci (s1, s2, s3) within the subarcuate fossa. Image not to scale.

The crista petrosa is typically quite pronounced (Figs. 13 and 14), but it is less defined in AMNH FM 47077. A deep superior petrosal sinus sulcus (SPSS) is often present between the crista petrosa and the subarcuate fossa and a sigmoid sinus sulcus (SSS) is often present caudal to the subarcuate fossa (Figs. 13C and 14). As described by Whitmore (1953) and O’Leary (2010), the subarcuate fossa (SaF) is shallow close to its opening but quickly expands into a large cavity (Fig. 14, 15). The fossa has a ventral fossula bordered by the posterior semicircular canal; when the volume of the subarcuate fossa is reconstructed, the fossula extends through the arch of the canal (Fig. 7C, D). The rest of the subarcuate fossa is wedged between the arches of the posterior and lateral semicircular canals and extends slightly caudal to both (Fig. 7C, D). A petromastoid canal (PmC), which transmits the subarcuate artery, pierces the floor of the subarcuate fossa rostrolateral to the ventral fossula and exits laterally just rostral to the mastoid region (Fig. 13A, B). Inside the subarcuate fossa, three sulci consistently extend from the petromastoid canal (Fig. 15). One sulcus (s1) travels medially and then curves dorsally along the wall of the subarcuate fossa, terminating before reaching the rim (Fig. 15). This sulcus is deep, particularly at its point of termination. A shallower sulcus (s2) extends rostrally but fades away before reaching the aperture (Fig. 15). The third sulcus (s3), which is also deep, extends rostrolaterally, often coming close to the rim of the fossa (Fig. 15). In FMNH PM 14560, this third sulcus extends past the rim to intersect with the superior petrosal sulcus, suggesting that it may have transmitted the subarcuate vein.

A wide and flat endocranial ridge (EcR) divides the subarcuate fossa from the internal acoustic meatus (IAM) (Fig. 14). The endocranial ridge laterally terminates in a broad expanse of bone that we call the suprameatal plate (SmP) (Fig. 14). A deep depression (“Depression” in Fig. 14) sits lateral to the subarcuate fossa and caudal to the ridge. The endocranial ridge typically has a lip that overhangs the internal acoustic meatus (IAM), partially obscuring the foramen acusticus superius (FAS) and giving the internal acoustic meatus a triangular appearance (Fig. 14). O’Leary (2010) described the internal acoustic meatus of AMNH VP 140343 as oval with a well-defined border expect caudally. Based on this description and associated figure, which shows an overhung foramen acusticus superius (O’Leary 2010: fig. 39), we would consider this specimen to also have a triangular internal acoustic meatus, but there is variation within the genus. The foramen acusticus superius is smaller than the foramen acusticus inferius (FAI) (Fig. 14). The two are either mediolaterally in line with each other or the foramen acusticus superius is just slightly rostral to the foramen acusticus inferius. The relative position does not appear to be influenced by phylogeny or ontogeny. The aperture of the facial canal (FC) sits rostrolateral in the foramen acusticus superius, and the superior vestibular area (SVA) is located just caudal to it, but it is often obscured from view by the overhanging endocranial ridge (Fig. 14). The tractus spiralis foraminosus (TSF) occupies the rostral portion of the foramen acusticus inferius (Fig. 14). Caudally, a small foramen singulare (FoS) is present, and the inferior vestibular area (IVA) lies just lateral to the foramen singulare (Fig. 14). The crista transversa (CrT) separating the foramina acusticus superius and inferius is a narrow ridge (Fig. 14). The rim of the internal acoustic meatus is distinct except medial or mediocaudal to the foramen acusticus inferius where it is level with the surrounding bone (Fig. 14) (O’Leary 2010).

The crista petrosa (Cr pet) dorsolaterally borders the subarcuate fossa and continues anteriorly to a flat shelf at the junction between the pars canalicularis and pars cochlearis (Figs. 13 and 14). The shelf is sometimes, but not always, developed into an anteromedial tuberosity (AmT) (possibly synonymous with the processus durante of Macintyre 1972) of variable size (Fig. 14). Rostromedial to the anteromedial tuberosity, the prefacial commissure (PfC) is a wide swath of bone that is slightly concave but lacks a prefacial commissure fossa (Fig. 14) (O’Leary 2010). The prefacial commissure lies rostrolateral to the internal acoustic meatus. The rest of the bone surrounding the internal acoustic meatus is similarly expansive, forming a large plate (O’Leary 2010). Rostrolaterally, there is a wide and shallow fossa (“Fossa” in Fig. 14) on the plate that may be for the trigeminal ganglion. Medially, this plate smoothly grades into the ventromedial flange (VmF), which works with the auditory bulla and the basioccipital to form the petrobasilar canal. In AMNH FM 47077, the ventromedial flange interdigitates with the basioccipital for part of its length. There is no basicapsular groove. The cochlear aqueduct (CA) is a thin slit just caudal to the ventromedial flange (Fig. 14). There is another, smaller projection that lies ventral and caudal to the cochlear aqueduct. It extends into a narrow ridge that contributes to the posterior border of the petrosal in endocranial view. We have not found a term for such a process in the literature, so we refer to it as the caudal endocranial process (CEP) (new term) (Fig. 14). The vestibular aqueduct (VA) is located just lateral to the caudal endocranial process, at the base of the endocranial ridge (Fig. 14). In AMNH FM 42298 and FMNH UC 493, there is a narrow groove (“Endo Groove” in Fig. 14) extending from the dorsolateral lip of the vestibular aqueduct aperture into the subarcuate fossa.

**Fig. 16:**
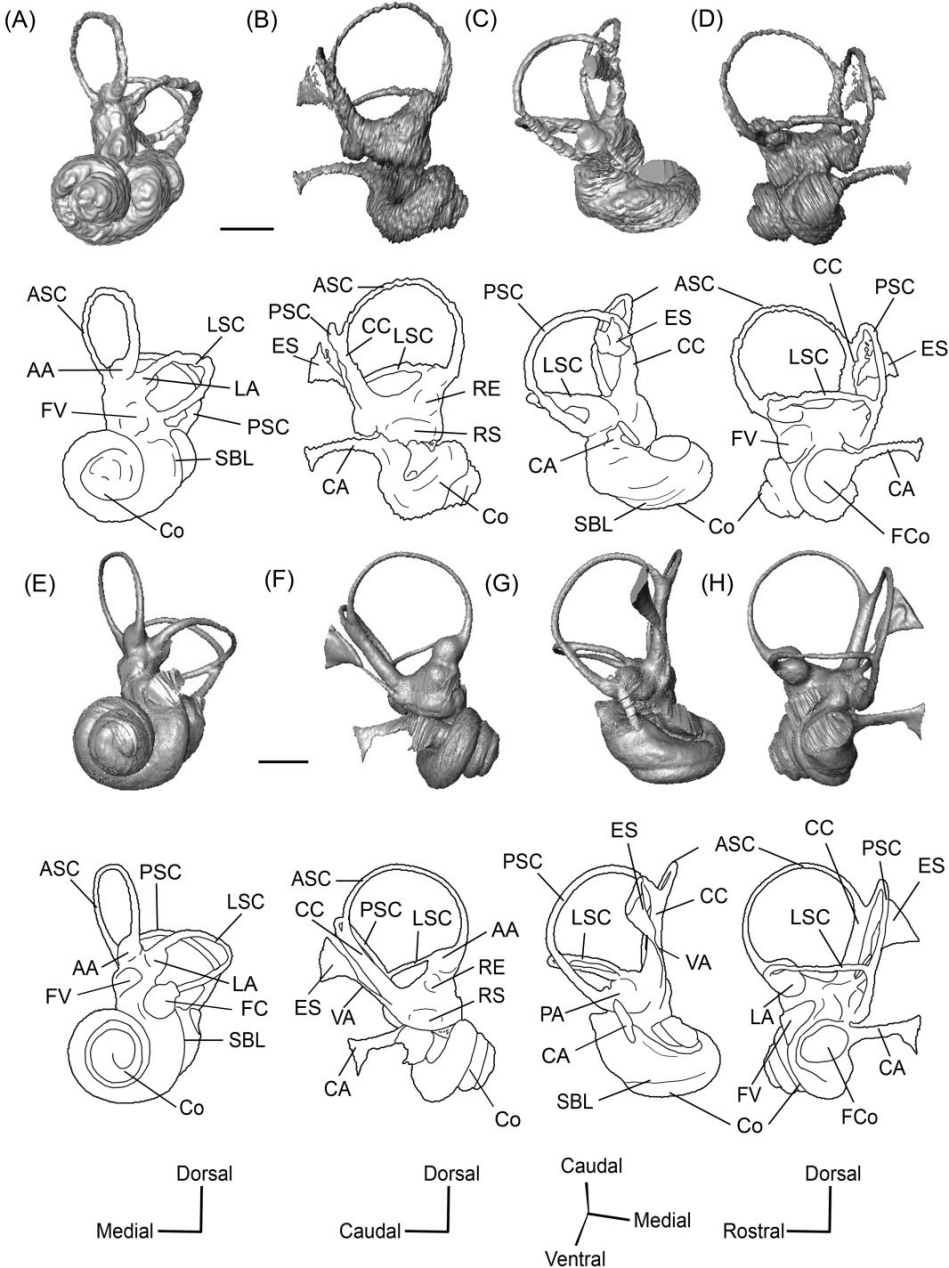
The left bony labyrinth of the camelids **A–D**, *Poebrotherium eximium* (AMNH FM 42298) and **E–H**, *Poebrotherium wilsoni* (FMNH UM 493) in rostral **(A, E)**, endocranial **(B, F)**, ventromedial **(C, G)**, and tympanic **(D, H)** view. Axes of orientation are shared by each column. Scale bars are shared by each row. **Abbreviations: AA**, anterior ampulla; **ASC**, anterior semicircular canal; **CA**, cochlear aqueduct; **CC**, common crus; **Co**, cochlea; **ES**, endolymphatic sac; **FCo**, fenestra cochleae; **FV**, fenestra vestibuli; **LA**, lateral ampulla; **LSC**, lateral semicircular canal; **PA**, posterior ampulla; **PSC**, posterior semicircular canal; **RE**, recessus ellipticus; **RS**, recessus sphaericus; **SBL**, secondary bony lamina; **VA**, vestibular aqueduct. Scale bars are 2 mm.

*Bony labyrinth.* The apex of the cochlea (Co) of three *Poebrotherium* specimens, including both specimens of *Po. eximium* (AMNH FM 47077; AMNH FM 42298), could not be reconstructed in detail, but these specimens match the others in gross morphology (Fig. 16). The length of the *Po. eximium* cochlea is likely slightly more than 21 mm to 23 mm based on measurements that include the incomplete apex (Supplementary Material 4), and it appears to have had between 2.5 and 3.5 turns. The cochlea of *Po. wilsoni* has an average length of around 30 mm and has between 3.25 and 3.50 turns (Supplementary Material 4). The cochlear length and cochlear aspect ratio do not greatly vary between the juvenile and adult *Po. wilsoni* specimens, but the cochlear aspect ratio is lower in *P. wilsoni* (0.59–0.62) than in *P. eximium* (0.66–0.72), and the two *Poebrotherium* sp. specimens have a cochlear aspect ratio similar to that of *P. eximium* (0.64–0.74) (Supplementary Material 4). The basal coil of the *Poebrotherium* cochlea is wide and there is a small gap between the first half of the basal turn and the start of the second turn (Fig. 16A, E). The rest of the coils are appressed to each other without gaps. The secondary bony lamina (SBL) creates a deep groove on the first half of the basal turn, and the primary bony lamina is present for most of the length of the cochlea (Fig. 16C, G).

The cochlear aqueduct (CA) is consistently a long and narrow passage extending from the base of the cochlea to beyond the posterior semicircular canal in both juveniles and adults (Fig. 16B–D, F–H). Like the oromerycids, it is a slit rather than a tube. It is directed caudomedially and terminates in a triangular perilymphatic sac. The vestibular aqueduct (VA) begins parallel to the common crus, but it curves slightly medially, and the endolymphatic sac (ES) originates well below the apex of the crus (Fig. 16B–D, F–H). The recessus sphaericus (RS) (sacculus) and the recessus ellipticus (RE) (utriculus) are present, but neither is particularly pronounced (Fig. 16B, F). The recessus sphaericus is larger in area, but the recessus ellipticus is more distinctive, forming a somewhat circular protrusion ventral to the anterior ampulla (Fig. 16C, F). All ampullae are well defined (Fig. 16A, E–H). The anterior semicircular canal (ASC) is the largest of the three, and the lateral semicircular canal (LSC) is the smallest (Fig. 16) (Supplementary Material 4). When viewed from above, the anterior semicircular canal is sigmoidal, having a distinctive S-shape to its arch with the rostral part of the curve trending medially and the caudal part of the curve trending laterally. The posterior and lateral semicircular canals have much less of a curve, typically sitting in a single plane. There is no consistent pattern in the angles between the semicircular canals, even within a specimen (Supplementary Material 4). Angles range between 82° and 100°, with some of the smallest and largest angles (79° and 94°) found in the same juvenile (FMNH UM 493). The other juveniles have a relatively small range of angles, so the large range in FMNH UM 493 is unlikely to be an ontogenetic signal. The lateral semicircular canal and the cochlea are typically set at an angle of around 35°, with the smallest angle being 30.34° and the largest angle being 45.53° (Supplementary Material 4). This distribution once again does not appear to be connected to phylogeny or late-stage ontogeny.

*Ear ossicles.* At least one ear ossicle was present in every specimen, but many were poorly preserved. A table of which specimens have which ossicles can be found in Supplementary Material 5. We could not determine if there is evidence of ontogenetic or interspecific variation, but juvenile ear ossicles have been compared to adults in the past (Wible and Spaulding 2012), and it is unlikely that doing so here poses a problem. None of the ossicles were found in their natural position, so orientations are based on the ossicles of *Vicugna vicugna* (Supplementary Material 3, Fig. S5; Supplementary Material 5).

**Fig. 17:**
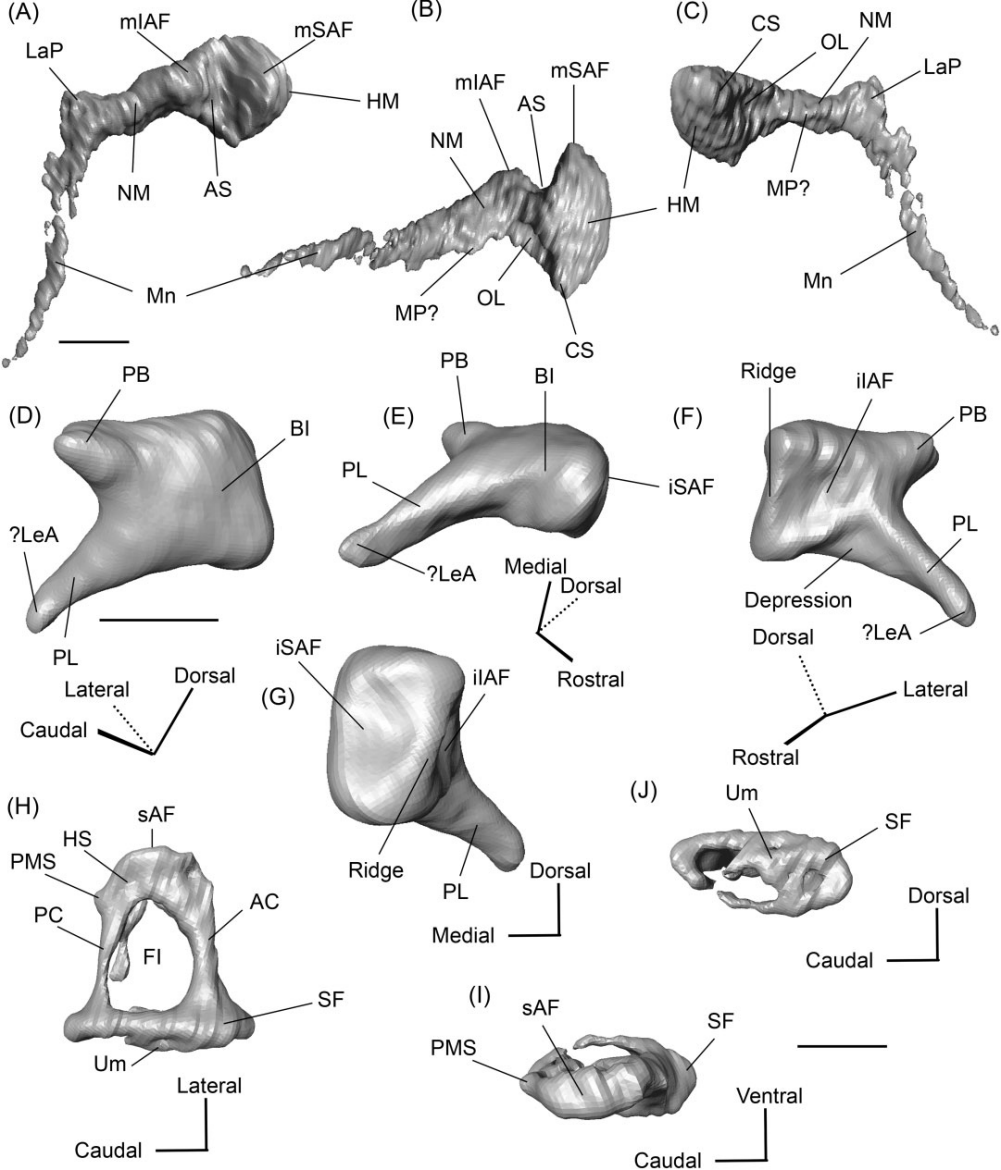
The right (mirrored) malleus of *Poebrotherium eximium* (AMNH FM 42298) (**A–C**), the left incus of *Poebrotherium* sp. (FMNH PM 14560) (**D–G**), and the left stapes of *Poebrotherium wilsoni* (FMNH CM 465) (**H–J**) in caudomedial (**A, D**), ventral (**B, E**), rostrolateral (**C, F**), rostral (**G**), dorsal (**H**), lateral (**I**), and medial (**J**) view. Orientations are based on *Vicugna vicugna*. Axes of orientation are shared by each view. Scale bars are shared by each bone. **Abbreviations: AC**, anterior crus; **AS**, mallear articular surface for the incus; **BI**, body of the incus; **CS**, capitular spine; **FI**, foramen intercrurale; **HM**, head of the malleus; **HS**, head of the stapes; **iIAF**, incudal inferior articular facet; **iSAF**, incudal superior articular facet; **LaP**, lateral process of the malleus; **LeA**, lenticular apophysis; **mIAF**, mallear inferior articular facet; **Mn**, manubrium; **MP**, muscular process; **mSAF**, mallear superior articular facet; **NM**, neck of the malleus; **OL**, osseous lamina; **PB**, processus brevis; **PC**, posterior crus; **PL**, processus longum; **PMS**, processus muscularis stapedius; **sAF**, stapedial articular facet; **SF**, stapedial footplate; **Um**, umbo. Scale bars are 1 mm.

The head of the malleus (HM) is pronounced and laterally expanded, coming to a point as a capitular spine (CS) (Fig. 17A–C). The articular surface for the incus (AS) forms a dorsolaterally-trending groove on the head and is bordered by two facets, with the rostromedially placed superior articular facet (mSAF) being much larger than the caudolaterally placed inferior articular facet (mIAF) (Fig. 17A, B). The groove and facets give the surface a saddle-like shape. Adjacent to the capitular spine is the osseous lamina (OL) (Fig. 17B, C), which appears to vary in size from small (e.g., FMNH PM 14560) to quite large (e.g., AMNH FM 42298), but this variation may be an artifact of scan quality and does not seem to be related to taxonomy or ontogeny. There may be a very small muscular process (MP) for the tensor tympani muscle ventrolateral to the osseous lamina, but this again is difficult to judge because of scan quality (Fig. 17B, C). The neck of the malleus (NM) is fairly straight and relatively long (Fig. 17A–C). The manubrium (Mn) is a long, extremely thin, straight triangular plate of bone that is only preserved in *Po. eximium* (AMNH FM 42298). It forms a slightly obtuse angle with the neck in the sagittal plane, and a large lateral process (LaP) sits at the junction between the two. Whether the manubrium is wider proximally than distally cannot be fully determined, but based on the digital models, it is fairly uniform in thickness. The distal tip is not greatly curved.

The body of the incus (BI) is square in shape (Fig. 17D–F). The medial side ranges from flat to gently convex while the lateral side is concave because of a ventrolateral depression (“Depression” in Fig. 17F). The rostral face of the incus is wider dorsally than ventrally, giving the surface a triangular appearance. However, there is clear intrageneric variation in this morphology—some specimens appear almost rectangular because of an unusually wide ventral side (Fig. 17G). Rostrally and laterally, there are two distinct facets that are part of the incudomallear joint. The lateral inferior articular facet (iIAF) is deeper than the rostral superior articular facet (iSAF), but the superior articular facet is broader (Fig. 17F, G), corresponding to the larger superior articular facet of the malleus. The rostral face of the incus, where the superior articular facet sits, narrows slightly ventrally, but not by much (Fig. 17G). The facets are separated by a prominent ridge (“Ridge” in Fig. 17F, G) that fits into the deep groove on the malleus (Fig. 7A, B). The processes brevis and longum are at an approximately 90° angle from each other. Although the relative length varies among individuals, the processus brevis (PB) is typically shorter and thicker than the processus longum (PL) (Fig. 17D–F), a morphology that is unusual in artiodactyls (Doran 1878). The distal portion of the processus longum is somewhat flattened, which may be the attachment for the lenticular apophysis (LeA), but the full structure does not appear to have been preserved (Fig. 17D–F).

The stapes of *Poebrotherium* is trapezoidal with long anterior and posterior crura (AC; PC) and a somewhat narrow foramen intercrurale (FI) that extends from the stapedial footplate (SF) to the head of the stapes (HS) (Fig. 17H–J). The two crura appear to be the same width, but the edges of the crura are incomplete. There is a distinct processus muscularis stapedius (PMS) at the top of the posterior crus (Fig. 17H, I). The head of the stapes is mediolaterally deep, and the articular facet (sAF) for the lenticular apophysis is narrow, ovate, and flat (Fig. 17H, I). The stapedial footplate is also ovate, although much larger than the head, and it is narrower caudally than rostrally (Fig. 17J). The rim of the stapedial footplate is fairly thin, and the center of the footplate is incomplete in the examined specimens, but an umbo (Um) does appear to be present (Fig. 17H–J). The stapedial footplate area (1.25 mm^2^ to 1.63 mm^2^) is consistently smaller than the fenestra vestibuli area (1.61 mm^2^ to 2.44 mm^2^) of the respective specimens (Supplementary Material 4; Supplementary Material 5), supporting the findings of Orliac and Billet (2016) and demonstrating a need to differentiate between the two measurements. The two values appear to be strongly correlated, with the specimen (FMNH PM 14560) with the smallest stapedial footplate area also having the smallest fenestra vestibuli area, and the specimen (FMNH UC 493) with the largest stapedial footplate area also having the largest fenestra vestibuli area (Supplementary Material 4).

#### Family Camelidae

##### Poebrotherium sp. (FMNH PM 14560)

We describe the petrosal of FMNH PM 14560 separately because it has a somewhat unusual morphology. The bony labyrinth and the ossicular chain morphology of FMNH PM 14560 do not greatly differ from that of other *Poebrotherium* specimens, and they are included in the preceding section, although it is worth noting that the rostral face of the FMNH PM 14560 incus is much squarer than that of the other *Poebrotherium* incudes examined. In terms of measurements, the bony labyrinth of FMNH PM 14560 is more similar to *Po. eximium* than to *Po. wilsoni*: it has a cochlear length of less than 30 mm, a high cochlear aspect ratio (0.64 left, 0.74 right), and the second smallest fenestra vestibuli area (1.61 mm^2^ left, 1.72 mm^2^ right) (Supplementary Material 4).

**Fig. 18:**
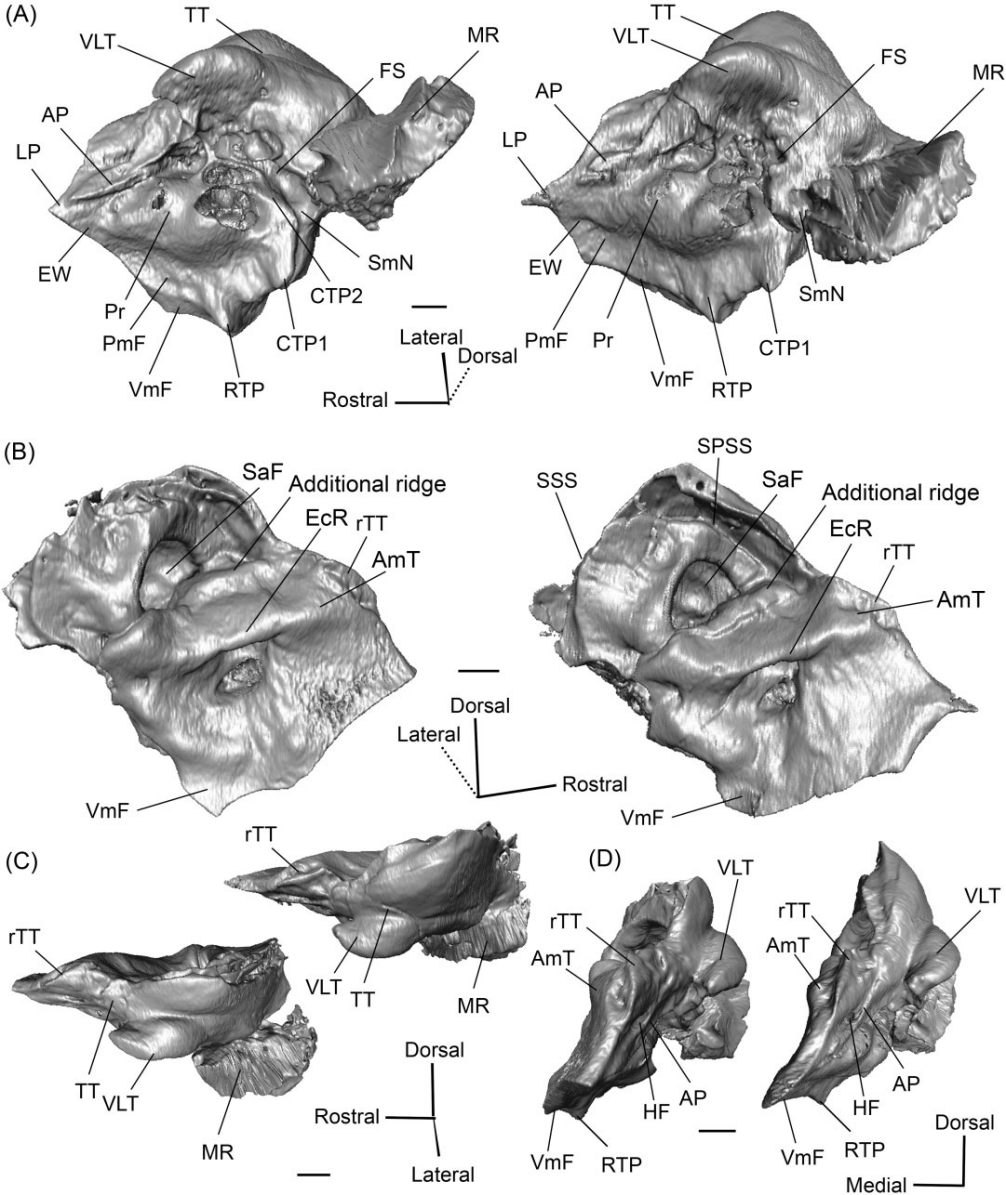
The left and right (mirrored) petrosals of *Poebrotherium* sp. (FMNH PM 14560) in **A**, tympanic; **B**, endocranial; **C**, dorsolateral; and **D**, rostral view. The left petrosal is on the left; the right petrosal is on the right. **Abbreviations: AmT**, anteromedial tuberosity; **AP**, anterior process of the tegmen tympani; **CTP1**, medial caudal tympanic process; **CTP2**, lateral caudal tympanic process; **EcR**, endocranial ridge; **EW**, epitympanic wing; **FS**, facial sulcus; **HF**, hiatus Fallopii; **LP**, lateral process of the epitympanic wing; **MR**, mastoid region; **PmF**, posteromedial flange; **Pr**, promontorium; **RTP**, rostral tympanic process; **SPSS**, superior petrosal sinus sulcus; **SSS**, sigmoid sinus sulcus; **rTT**, rostral tegmen tympani; **SaF**, subarcuate fossa; **SmN**, stylomastoid notch; **TT**, tegmen tympani; **VLT**, ventrolateral tuberosity; **VmF**, ventromedial flange. Scale bars are 2 mm.

On the left petrosal, the epitympanic wing (EW) forms an almost perfect triangle that terminates at the lateral process of the epitympanic wing (LP) (Fig. 18A). There is no medial process of the epitympanic wing. The distinction between the lateral process and the rest of the wing is more pronounced on the right petrosal, but it is still very triangular. On both petrosals, rather than forming a continuous shelf around the rostral and medial sides of the promontorium (Pr), as is the standard condition in artiodactyls (O’Leary 2010), the epitympanic wing travels caudomedially in a nearly straight line before intersecting with the posteromedial flange (PmF) (Fig. 18A). This intersection occurs far more rostrally on the right petrosal. The abrupt intersection greatly changes the profile of the FMNH PM 14560 petrosals as there is only a thin strip of bone rostromedially bordering the promontorium. The posteromedial flange is much broader than the ventromedial flange (VmF), the latter of which only becomes visually distinct along the caudal portion of the posteromedial flange (Fig. 18A, B). Rostrally, it is a narrow and smooth strip of bone that is not visible in tympanic view (Fig. 18D). Because of this morphology, the ventromedial border of the petrosals is flat rather than keeled, and there is a sharp change in orientation where the ventromedial flange becomes visible. The rostral tympanic process (RTP) is large and extends far medial to the promontorium (Fig. 18A, D). The specimen has a very narrow crista interfenestralis, with the ratio between the crista interfenestralis and vestibular fossula widths being 0.56 (left) and 0.65 (right), which is much narrower than that of the *Po. eximium* and *Po. wilsoni* specimens (Supplementary Material 4). The morphology of the rest of the tympanic pars cochlearis is like that of other *Poebrotherium* specimens, although the rostral portion of the promontorium may be slightly more convex than usual, particularly in the left petrosal.

As in most specimens of *Poebrotherium*, the facial sulcus (FS) travels through the stylomastoid notch (SmN) and wraps ventromedially around the caudal portion of the petrosal, leaving a deep groove (Fig. 18A). However, unlike in most individuals, the medial caudal tympanic process (CTP1) is expanded into a notable flange that ventrally borders this groove (Fig. 18A). In addition, the mastoid region (MR) is more greatly expanded than is normal of *Poebrotherium* specimens (Fig. 18A–C). On the tegmen tympani (TT), the ventrolateral tuberosity (VLT) is much larger than in other specimens of *Poebrotherium* (Fig. 18A, C, D). The anterior process of the tegmen tympani (AP) has the same morphology as other specimens, but on the left petrosal, it extends to almost the tip of the lateral process of the epitympanic wing (Fig. 18A–D).

Unlike in most *Poebrotherium* specimens, where the tegmen tympani medial to the hiatus Fallopii (HF) forms a thin ridge that intersects the blunt anteromedial tuberosity (AmT) (Fig. 18B, D), the rostral tegmen tympani (rTT) of FMNH PM 14560 is greatly expanded (Fig. 18B–D). A ridge is present, but the base of the ridge is wider than usual and laterally forms a curved shelf. The endocranial ridge (EcR) of FMNH PM 14560 is extremely wide and the area around the subarcuate fossa (SaF) is unusually “crinkled” with an additional ridge immediately rostral to the fossa; this results in the aperture of the subarcuate fossa being compressed with a ventromedially flat, rather than curved, border (Fig. 18B). This morphology is present bilaterally and, given that the petrosals and surrounding bones are intact, it appears to be biological rather than taphonomic. As noted previously, unlike in other *Poebrotherium* specimens, the third subarcuate fossa sulcus (s3) of FMNH PM 14560 makes direct contact with the sulcus for the superior petrosal sinus.

#### Family Camelidae

##### Paratylopus primaevus (AMNH FM 9806)

###### Petrosal

**Fig. 19:**
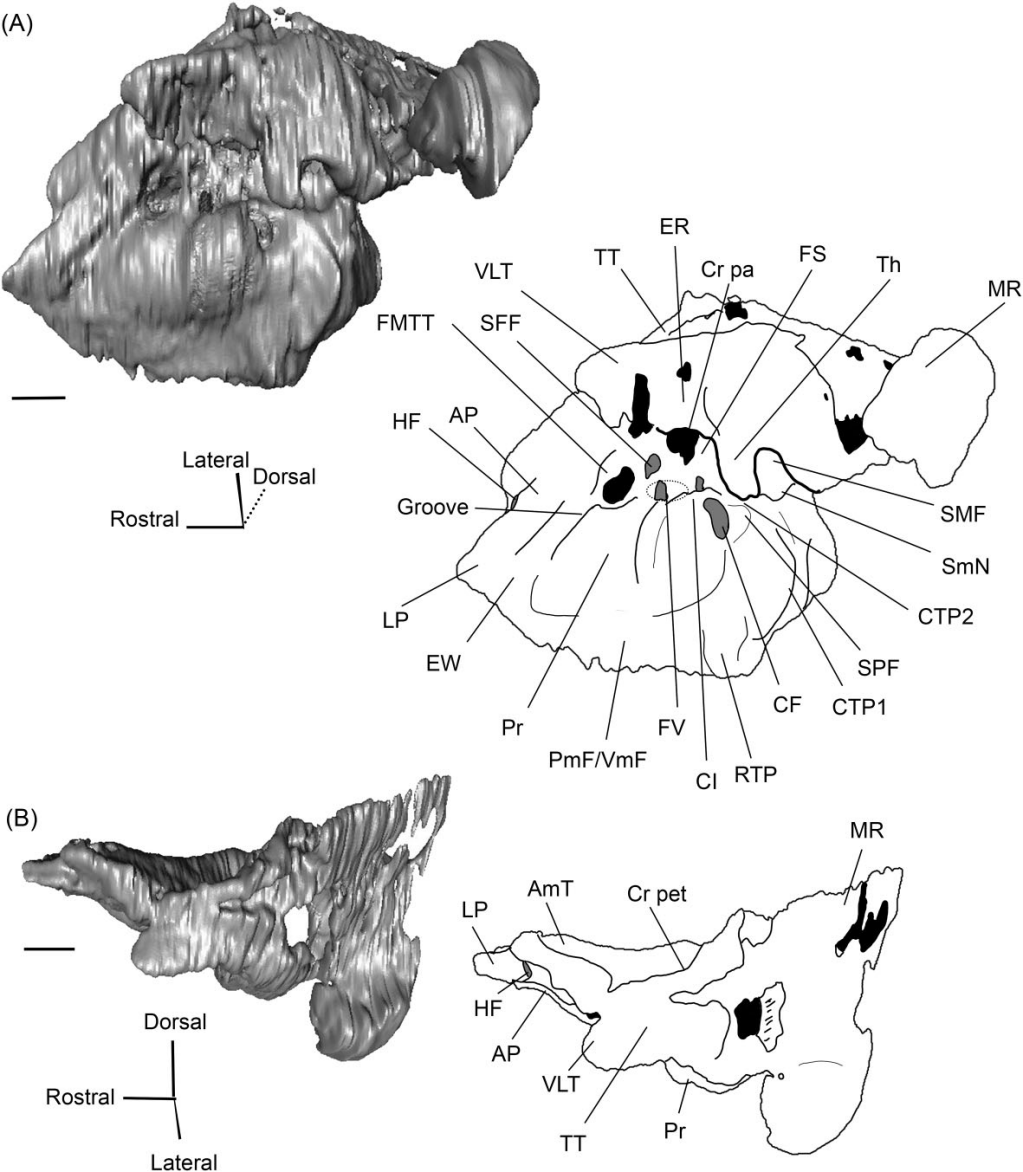
The left petrosal of the camelid *Paratylopus primaevus* (AMNH 9806) in tympanic **(A)** and dorsolateral **(B)** view. **Abbreviations: AmT**, anteromedial tuberosity; **AP**, anterior process of the tegmen tympani; **CF**, cochlear fossula; **CI**, crista interfenestralis; **Cr pa**, crista parotica; **Cr pet**, crista petrosa; **CTP1**, medial caudal tympanic process; **CTP2**, lateral caudal tympanic process; **ER**, epitympanic recess; **EW**, epitympanic wing; **FMTT**, fossa for the muscularis tensor tympani; **FS**, facial sulcus; **FV**, fenestra vestibuli; **HF**, hiatus Fallopii; **LP**, lateral process of the epitympanic wing; **MR**, mastoid region; **PmF**, posteromedial flange; **Pr**, promontorium; **RTP**, rostral tympanic process; **SFF**, secondary facial foramen; **SMF**, stapedial muscle fossa; **SmN**, stylomastoid notch; **SPF**, saccus posticus fossa; **Th**, tympanohyal; **TT**, tegmen tympani; **VLT**, ventrolateral tuberosity; **VmF**, ventromedial flange. Scale bars are 2 mm.

**Fig. 20:**
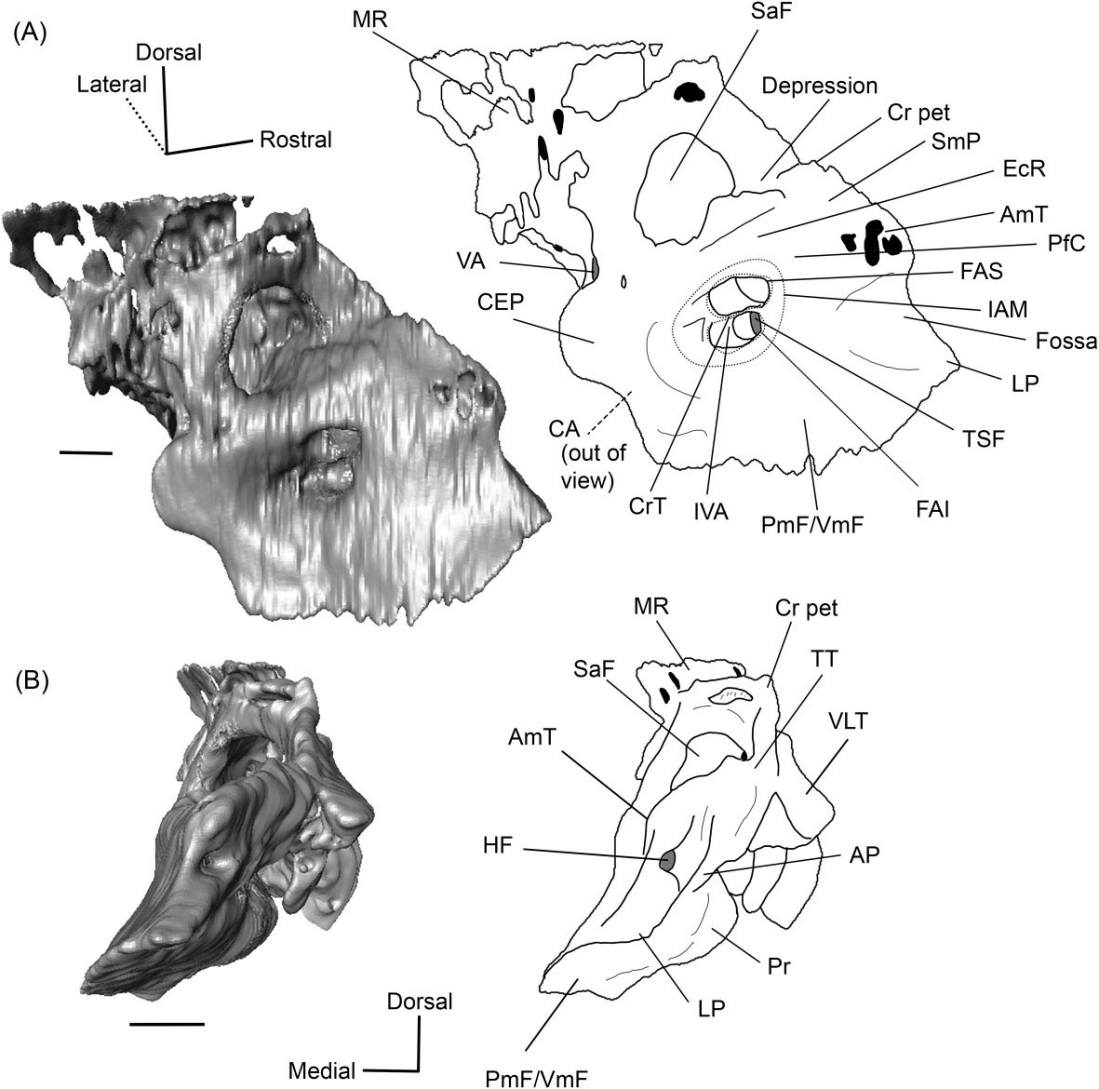
The left petrosal of the camelid *Paratylopus primaevus* (AMNH 9806) endocranial **(A)** and rostral **(B)** view. **Abbreviations: AmT**, anteromedial tuberosity; **AP**, anterior process of the tegmen tympani; **CA**, cochlear aqueduct; **CEP**, caudal endocranial process; **Cr pet**, crista petrosa; **CrT**, crista transversa; **EcR**, endocranial ridge; **FAI**, foramen acusticus inferius; **FAS**, foramen acusticus superius; **HF**, hiatus Fallopii; **IAM**, internal acoustic meatus; **IVA**, inferior vestibular area; **LP**, lateral process of the epitympanic wing; **MR**, mastoid region; **PfC**, prefacial commisure; **PmF**, posteromedial flange; **Pr**, promontorium; **SaF**, subarcuate fossa; **SmP**, suprameatal plate; **TSF**, tractus spiralis foraminosus; **TT**, tegmen tympani; **VA**, vestibular aqueduct; **VLT**, ventrolateral tuberosity; **VmF**, ventromedial flange. Scale bars are 2 mm.

The petrosal of *Pa. primaevus* has the large epitympanic wing (EW) characteristic of camelids (Fig. 19A). The lateral process of the epitympanic wing (LP) ends in a point (Figs. 19 and 20). There does not appear to be a medial process of the epitympanic wing, but the border of the right petrosal is somewhat broken so the absence cannot be confirmed bilaterally. Ventrally, the epitympanic wing smoothly grades into a large flange (Fig. 19).

The promontorium (Pr) is convex along its entire length (Fig. 19). There is no evidence of a transpromontorial or stapedial sulcus, but this may simply be because of poor preservation. As in *Poebrotherium*, there is a clear groove extending rostrally from the fossa for the muscularis tensor tympani (FMTT) (“Groove” in Fig. 19A). The fenestra vestibuli (FV) is lateral to the promontorium and appears to be oval in outline with a rostrocaudal long axis (Fig. 19A), but there was not enough clarity in the scan to reconstruct the fenestra vestibuli and vestibular fossula with high fidelity. The secondary facial foramen (SFF) is just lateral to the fenestra vestibuli (Fig. 19A). The fenestra vestibuli and the aperture of the cochlear fossula (CF) are separated by the crista interfenestralis (CI) (Fig. 19A). The cochlear fossula is caudally oriented and sits lateral to the rostral tympanic process, the latter of which extends to the ventromedial border of the petrosal. A small saccus posticus fossa (SPF) is just caudal to the cochlear fossula (Fig. 19A). The termination of the rostral tympanic process (RTP) typically demarcates the transition from the posteromedial flange (PmF) to the ventromedial flange (VmF) in camelids (Figs. 12 and 18). In *Pa. primaevus*, the long rostral tympanic process means that either the posteromedial flange is indistinct, or it is large and contributes to the ventromedial border of the bone (Fig. 19A). The latter is likely: there is a change in flange orientation, similar to what is seen in other camelids, just lateral to the ventromedial border. Because this region was not well defined in the µCT scan, it is possible that the ventromedial flange is more extensive but could not be fully reconstructed, in which case the posteromedial flange may be large but not truly contribute to the ventromedial border.

On the tympanic pars canalicularis, the fossa for the muscularis tensor tympani is small and does not excavate the tegmen tympani (TT) (Fig. 19A). The facial sulcus (FS) extends caudally from the secondary facial foramen as a broad trough (Fig. 19A). There is no visible distinction between the facial sulcus and the fossa for the stapedial muscle (SMF), but this is likely because of poor scan quality (Fig. 19A). This area is overhung by the crista parotica (Cr pa), to which a portion of the tympanohyal (Th) is fused (Fig. 19A). Together, the tympanohyal and petrosal form the stylomastoid foramen, and the petrosal carries a stylomastoid notch (SmN) (Fig. 19A). The medial caudal tympanic process (CTP1) lies adjacent to the stylomastoid notch (Fig. 19A). It is a narrow flange of bone that flares laterally with a sulcus extending from the facial sulcus/stapedial muscle fossa running medially along its dorsal border. The rostral and caudal tympanic processes sit very close together, so much so that only a small notch separates the two. The area lateral to the aperture of the cochlear fossula and medial to the facial sulcus/stapedial muscle fossa is a raised ridge that is likely the lateral portion of the caudal tympanic process (CTP2) (Fig. 19A). The mastoid region (MR) could not be fully reconstructed, but it is clearly wedge-shaped (Figs. 19 and 20).

The tegmen tympani is moderately inflated and relatively flat when viewed rostrally (Fig. 20B). There is a large ventrolateral tuberosity (VLT) that forms a shelf over the indistinct epitympanic recess (ER), which lacks a fossa incudis (Figs. 19 and 20B). The ventrolateral tuberosity is narrower than in *Poebrotherium*, which is best observed in rostral view (Fig. 20B). The anterior process of the tegmen tympani (AP) extends rostrally to the epitympanic wing and becomes confluent with the lateral process of the wing (Figs. 19 and 20B). It is flush with the tympanic surface and carries a dorsolateral ridge, but it is large enough to contribute to the border of the petrosal. The hiatus Fallopii (HF) sits on the rostral face of the petrosal, just caudal to the lateral process of the epitympanic wing and dorsomedial to the anterior process of the tegmen tympani (Figs. 19 and 20B). There is no tegmen tympani fossa or tegmen tympani foramen.

The endocranial pars canalicularis is dominated by a large and spherical subarcuate fossa (SaF) (Fig. 20A). It occupies almost the entire space enclosed by the semicircular canals and, while there is some breakage in the region, there is clearly a small fossula that extends through the arch of the posterior semicircular canal (Fig. 7E). Whether a petromastoid canal is present could not be determined because the caudal portion of the subarcuate fossa is not completely preserved. There is a slight depression just rostral to the subarcuate fossa on the left petrosal (Fig. 20A), but not on the right one. A long crista petrosa (Cr pet) borders the endocranial pars canalicularis: it extends caudally over the subarcuate fossa and rostrally to a large anteromedial tuberosity (AmT) (Figs. 19B and 20). There is no indication of a superior petrosal sinus sulcus, and the area in which the sigmoid sinus sulcus would lie is broken. The suprameatal plate (SmP) is just caudal to the anteromedial tuberosity (Fig. 20A). A flat and broad endocranial ridge (EcR) separates the subarcuate fossa from the internal acoustic meatus (IAM) and slightly overhangs the foramen acusticus superius (FAS) on both petrosals (Fig. 20A). The border of the internal acoustic meatus is mostly well defined, but the caudomedial portion is flush with the surrounding bone, giving the meatus a somewhat triangular shape. The foramen acusticus superius is slightly smaller than the foramen acusticus inferius (FAI) and the two lie in the same mediolateral plane separated by a narrow crista transversa (CrT) (Fig. 20A). Because of the overhanging endocranial ridge, the aperture of the facial canal and the superior vestibular area are obscured from view. In the foramen acusticus inferius, the tractus spiralis foraminosus (TSF) is visible, but the foramen singulare is recessed caudally out of view (Fig. 20A). The inferior vestibular area (IVA) is a depression rostrolateral to the foramen singulare (Fig. 20A). The area rostral to the internal acoustic meatus, including the prefacial commissure (PfC), is expanded into a flat plate only disrupted by a shallow fossa (“Fossa” in Fig. 20A) between the anteromedial tuberosity and the lateral process of the epitympanic wing. This fossa was likely associated with the trigeminal ganglion in life and should not be confused with the prefacial commissure fossa, which is absent. The area medial to the internal acoustic meatus is similarly expanded because of the large posteromedial (and ventromedial) flange. The cochlear aqueduct (CA) exits at the caudal end of the ventromedial flange (Fig. 20A). A large wing-like caudal endocranial process (CEP) lies entirely posterior to the cochlear aqueduct and contributes to the border of the petrosal in endocranial view (Fig. 20A). The vestibular aqueduct (VA) lies lateral to the caudal endocranial process (Fig. 20A).

**Fig. 21:**
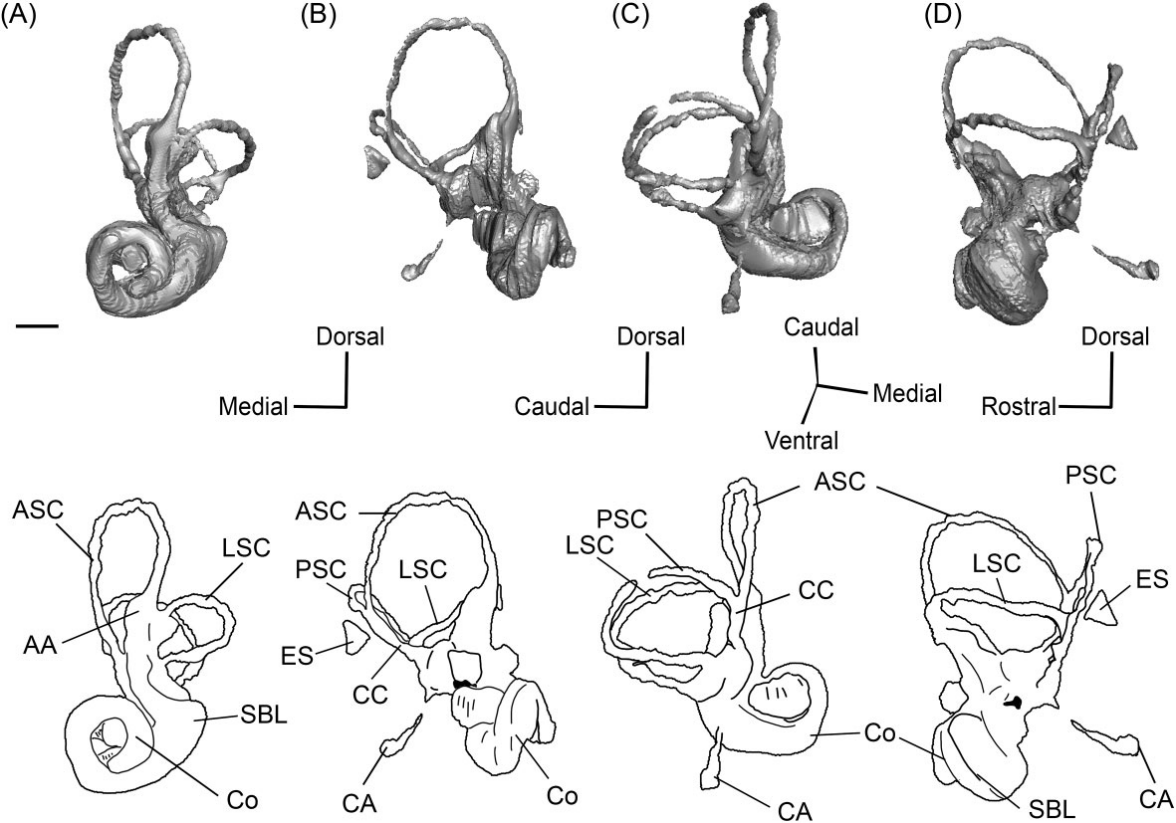
The left bony labyrinth of the camelid *Paratylopus primaevus* (AMNH 9806) in rostral **(A)**, endocranial **(B)**, ventromedial **(C)**, and tympanic **(D)** view. **Abbreviations: AA**, anterior ampulla; **ASC**, anterior semicircular canal; **CA**, cochlear aqueduct; **CC**, common crus; **Co**, cochlea; **ES**, endolymphatic sac; **LSC**, lateral semicircular canal; **PSC**, posterior semicircular canal; **SBL**, secondary bony lamina. Scale bar is 2 mm.

*Bony labyrinth.* The cochlea makes more than one full turn and is more than 16.25 mm long, but the apical coils could not be reconstructed so the length and total number of turns cannot be determined (Fig. 21) (Supplementary Material 4). There is a gap between at least the first half of the basal turn and the following turns, and the distinct groove for the secondary bony lamina (SBL) is preserved on the first half of the basal turn (Fig. 21A–D). The primary bony lamina could not be identified. The cochlear aqueduct (CA) is long, extending beyond the posterior semicircular canal (Fig. 21B–D). The recessus sphaericus and recessus ellipticus (utriculus and sacculus, respectively) could not be reconstructed with high fidelity, nor could the ampullae of the semicircular canals, although the anterior ampulla (AA) is certainly present (Fig. 21A). The vestibular aqueduct closely follows the path of the common crus (CC) of the semicircular canals before traveling medial to the posterior semicircular canal (PSC) to exit the petrosal via the endolymphatic sac (ES) (Fig. 21B–D). The anterior semicircular canal (ASC) has the largest arc radius, and the lateral semicircular canal (LSC) has the smallest, but their proportions greatly differ: the anterior semicircular canal is taller than wide while the lateral semicircular canal is much wider than tall (Supplementary Material 4). Indeed, the lateral semicircular canal is the widest and shortest of the three canals (Supplementary Material 4). All three canals are slightly sigmoidal, but the curvature of the anterior semicircular canal is much more pronounced. Given the preservation of the specimen, the shapes of the posterior and lateral semicircular canals may not be entirely accurate. The common crus is relatively short, being slightly less than half as tall as the anterior semicircular canal (Supplementary Material 4). The angles between the semicircular canals range from 88° to 100° (Supplementary Material 4). The angle between the lateral semicircular canal and the cochlea is slightly over 42°, but this could only be measured in the left bony labyrinth and may be slightly inaccurate (Supplementary Material 3; Supplementary Material 4).

**Fig. 22:**
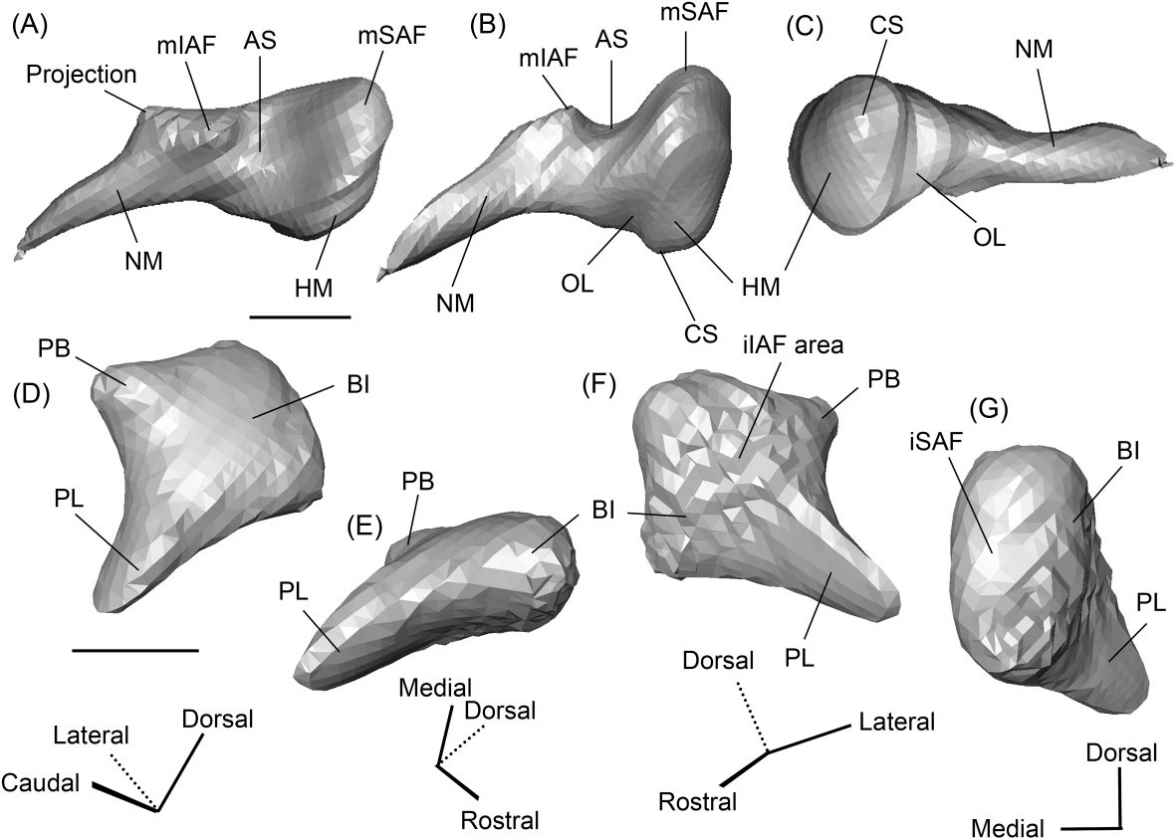
The left malleus (**A–C**) and incus (**D–G**) of *Paratylopus primaevus* (AMNH FM 9806) in caudomedial (**A, D**), ventral (**B, E**), rostrolateral (**C, F**), and rostral (**G**) view. Orientations are based on *Vicugna vicugna*. Axes of orientation are shared by each view. Scale bars are shared by each bone. **Abbreviations: AS**, mallear articular surface for the incus; **BI**, body of the incus; **CS**, capitular spine; **HM**, head of the malleus; **iIAF**, incudal inferior articular facet; **iSAF**, incudal superior articular facet; **mIAF**, mallear inferior articular facet; **mSAF**, mallear superior articular facet; **NM**, neck of the malleus; **OL**, osseous lamina; **PB**, processus brevis; **PL**, processus longum. Scale bars are 1 mm.

*Ear ossicles*. The left malleus and incus of AMNH FM 9806 were partially preserved, although not in high quality. The malleus appears to be in the approximately correct biological position, but the bone is incomplete. There is a clear articular surface for the incus (AS) on the head of the malleus (HM), the latter of which is ventrolaterally expanded (Fig. 22A, B). The articular surface is bordered by superior and inferior articular facets (SAF; IAF), although the superior articular facet is not very distinct from the rest of the head (Fig. 22A, B). The inferior facet is much smaller than the superior facet but still contributes to the saddle-shaped appearance of the surface. A small capitular spine (CS) may be present, but because of the low scan resolution, it cannot be identified with confidence, and the osseous lamina (OL) is a very shallow indentation (Fig. 22B, C). There is a small projection (“Projection”) adjacent to the inferior articular facet (Fig. 22A), which is likely either an unidentified structure or an artifact of preservation. A long neck (NM) extends from the head of the malleus (Fig. 22A–C). It is broken proximal to the lateral process and the manubrium.

The body of the incus (BI) is square-shaped (Fig. 22D–F) with a relatively narrow rostral face (Fig. 22G). The dorsal side of the body is gently convex. Because of the low resolution and scan quality, the superior and inferior articular facets are not apparent, but the rostral face is slightly indented where the superior articular facet (iSAF) is likely to be (Fig. 22G). The area where the inferior articular facet would be does not preserve the morphology of the bone (Fig. 22F). The angle between the processus brevis (PB) and the processus longum (PL) is more than 90° (Fig. 22D, F). The processus brevis is extremely short, and while the full length may not be captured in the scan, based on higher-resolution scans of *Poebrotherium*, the difference in length is likely real if slightly overexaggerated.

#### Family Camelidae

##### Stevenscamelus franki (TMM VP 40504–149)

###### Petrosal

**Fig. 23:**
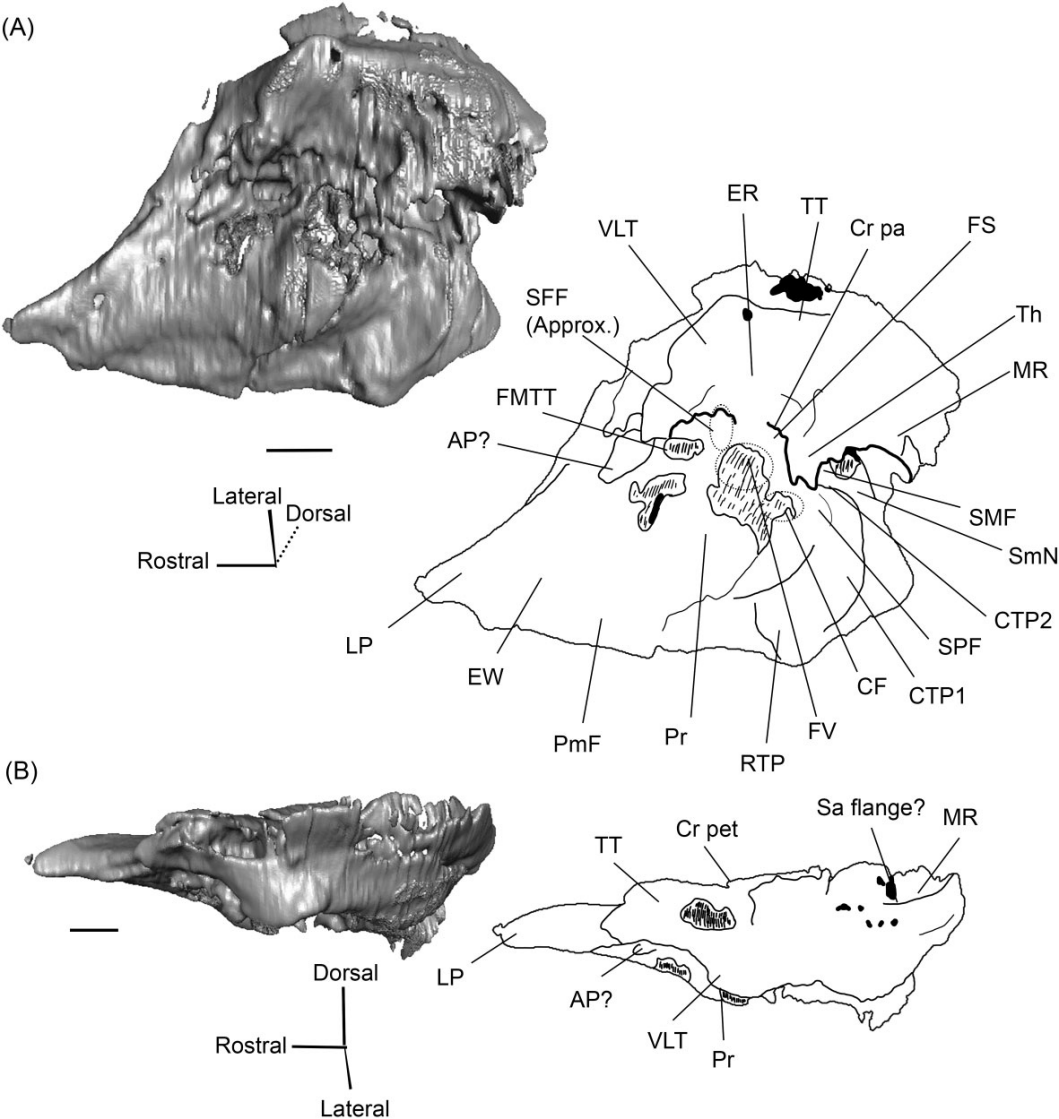
The right (mirrored) petrosal of the camelid *Stevenscamelus franki* (TMM VP 40504–149) in tympanic **(A)** and dorsolateral **(B)** view. **Abbreviations: AP**, anterior process of the tegmen tympani; **Approx.**, approximate; **CF**, cochlear fossula; **Cr pa**, crista parotica; **Cr pet**, crista petrosa; **CTP1**, medial caudal tympanic process; **CTP2**, lateral caudal tympanic process; **ER**, epitympanic recess; **EW**, epitympanic wing; **FMTT**, fossa for the muscularis tensor tympani; **FS**, facial sulcus; **FV**, fenestra vestibuli; **LP**, lateral process of the epitympanic wing; **MR**, mastoid region; **PmF**, posteromedial flange; **Pr**, promontorium; **RTP**, rostral tympanic process; **SFF**, secondary facial foramen; **SMF**, stapedial muscle fossa; **SmN**, stylomastoid notch; **SPF**, saccus posticus fossa; **Th**, tympanohyal; **TT**, tegmen tympani; **VLT**, ventrolateral tuberosity. Scale bars are 2 mm.

**Fig. 24:**
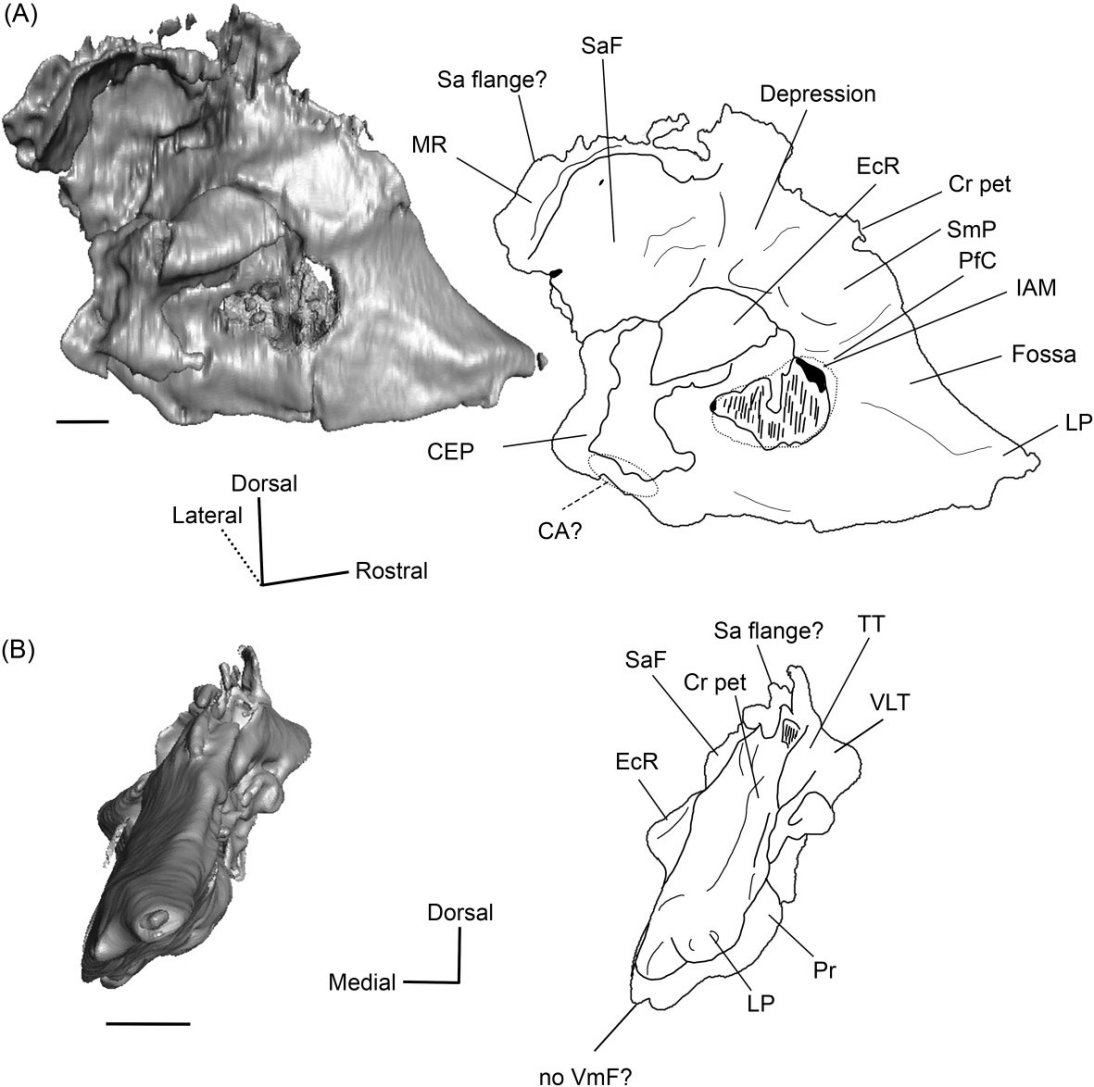
The right (mirrored) petrosal of the camelid *Stevenscamelus franki* (TMM VP 40504–149) in endocranial **(A)** and rostral **(B)** view. **Abbreviations: CA**, cochlear aqueduct; **CEP**, caudal endocranial process; **Cr pet**, crista petrosa; **EcR**, endocranial ridge; **IAM**, internal acoustic meatus; **LP**, lateral process of the epitympanic wing; **MR**, mastoid region; **PfC**, prefacial commissure; **Pr**, promontorium; **Sa flange**, subarcuate flange; **SaF**, subarcuate fossa; **SmP**, suprameatal plate; **TT**, tegmen tympani; **VLT**, ventrolateral tuberosity; **VmF**, ventromedial flange. Scale bars are 2 mm.

The petrosal of *Stevenscamelus franki* is badly broken in some regions, but the overall morphology is still discernible. A large epitympanic wing (EW) extends far rostral to the promontorium (Pr) and terminates in a lateral process (LP) (Figs. 23 and 24). Because of potential taphonomic deformation, we cannot confirm the presence of the medial process of the epitympanic wing. The epitympanic wing smoothly grades into what appears to be the posteromedial flange (PmF) (Fig. 23A). This flange extends below the promontorium, but not for any great depth, ending at approximately the same level as the lateral process of the epitympanic wing. It is possible that the more ventral extension of the flange was not preserved, but the morphology is present bilaterally and there is no indication of additional breakage in the µCT scan slices. The rostral tympanic process (RTP) projects slightly below the posteromedial flange with no indication that an additional flange is associated with it (Fig. 23A). If this is the full posteromedial flange and there is no postmortem damage, then *S. franki* lacks a ventromedial flange.

The promontorium is not fully preserved, but it was clearly hemi-ellipsoid and convex (Figs. 23 and 24B). No transpromontorial sulci could be identified, nor could the cochlear fossula (CF), fenestra vestibuli (FV), and vestibular fossula be fully reconstructed, but the secondary facial foramen (SFF) appears to have opened lateral to the rostral border of the fenestra vestibuli, and there is a clear saccus posticus fossa (SPF) caudal to the cochlear fossula (Fig. 23A).

The fossa for the muscularis tensor tympani (FMTT) is a shallow oval depression that does not look to have excavated the tegmen tympani (Fig. 23A). In the right petrosal, the thin bone forming the fossa is broken, exposing the passage for the greater petrosal nerve, but we could not identify its exit via the hiatus Fallopii (Fig. 23A). Caudal to the fossa and the secondary facial foramen, the facial sulcus (FS) extends posteriorly to the stylomastoid notch (SmN) and is roofed by the crista parotica (Cr pa) (Fig. 23A). Close to the stylomastoid notch, the fossa for the stapedial muscle (SMF) joins the facial sulcus to form a broader indentation (Fig. 23A). Both structures are partially roofed by the tympanohyal (Th) (Fig. 23A). A medially directed groove extends from the facial sulcus and stylomastoid notch, wrapping posteriorly around the medial caudal tympanic process (CTP1), the latter of which forms a small wing (Fig. 23A). The lateral caudal tympanic process (CTP2) extends rostrally from the medial portion to laterally border the cochlear fossula (Fig. 23A). The caudal and rostral tympanic processes are nearly in line with each other and are not separated by a notch. The mastoid region (MR) was broken and could not be fully reconstructed (Figs. 23 and 24).

Like most early camelids, the tegmen tympani (TT) is moderately inflated with a ventrolateral tuberosity (VLT) that roofs an indistinct epitympanic recess (ER) (Figs. 23 and 24). The ventrolateral tuberosity of *S. franki* projects slightly lateral to the rest of the tegmen tympani, but not to the same extent as in some specimens of *Poebrotherium* (e.g., FMNH UM 493; FMNH PM 14560) and *Pa. primaevus*, and it is greatly reduced in size (Figs. 13C, D, 18D and 20B). There is some indication of the typical early camelid anterior process (AP), but the area rostral to the fossa for the muscularis tensor tympani is mostly smooth, so it is not clear whether an anterior process was truly present (Fig. 23A). The hiatus Fallopii could not be located.

The dorsolateral border of the petrosal is not intact, but what is likely the remnant of a crista petrosa (Cr pet) and potentially a subarcuate flange (Sa flange) is discernable (Figs. 23B and 24). The subarcuate fossa (SaF) is only partially preserved; from what remains, it clearly had a large and circular aperture (Fig. 24A). Based on the caudal extent of the subarcuate fossa seen in the right petrosal (Fig. 24A), it may not have been as deep as that of other early camelids, but the evidence for this is not strong. Even with poor preservation, the typical camelid depression lateral to the subarcuate fossa is clearly present (“Depression” in Fig. 24A). The subarcuate fossa and internal acoustic meatus (IAM) are separated by an endocranial ridge (EcR) that rostrally terminates in a large suprameatal plate (SmP) (Fig. 24A). The anteromedial tuberosity appears to be absent. There is a wide fossa (“Fossa” in Fig. 24A) on the pars cochlearis rostral to the internal acoustic meatus and the prefacial commissure (PfC) that extends to almost the termination of the lateral process of the epitympanic wing. This fossa was possibly associated with the trigeminal ganglion in life. The internal acoustic meatus is not fully intact, but from what is preserved, its rostral and medial borders appear to have been flush with the surrounding bone. The exits of the cochlear and vestibular aqueducts were not preserved, but a caudal endocranial process (CEP) is bilaterally present (Fig. 24A). It is a long wing that is separated from the rest of the endocranial face by a fissure; this fissure likely contained the exit of the cochlear aqueduct (CA) (Fig. 24A).

**Fig. 25:**
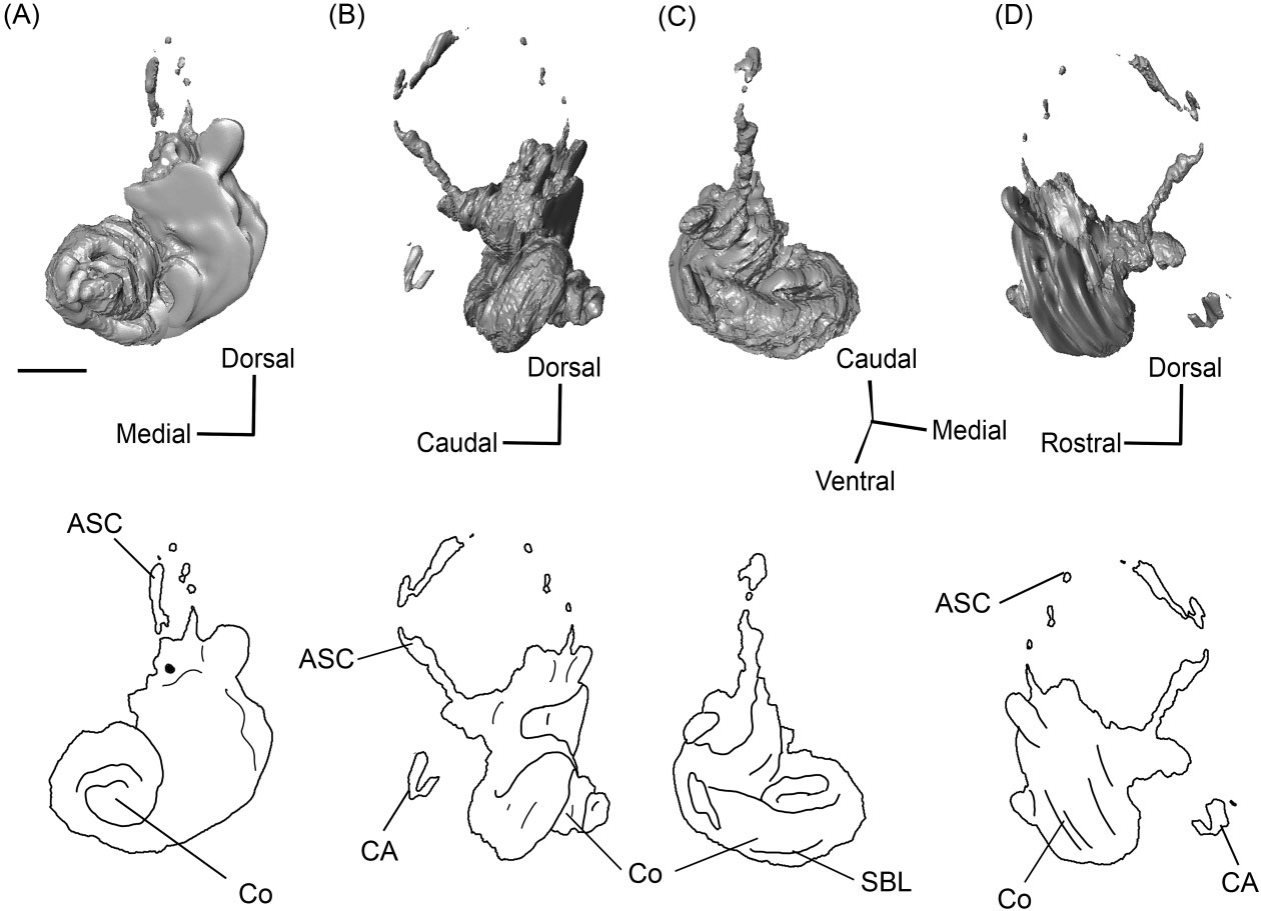
The left bony labyrinth of the camelid *Stevenscamelus franki* (TMM VP 40504–149) in rostral **(A)**, endocranial **(B)**, ventromedial **(C)**, and tympanic **(D)** view. **Abbreviations: ASC**, anterior semicircular canal; **CA**, cochlear aqueduct; **Co**, cochlea; **SBL**, secondary bony lamina. Scale bar is 2 mm.

*Bony labyrinth.* Although the bony labyrinth is poorly preserved, portions of the structure were reconstructed (Fig. 25). The length of the cochlea cannot be accurately measured, but it is more than 18 mm long, it appears to have at least 2.5 turns, and it has an approximate cochlear ratio of 0.68 (see Supplementary Material 3 for notes; Supplementary Material 4). The basal turn of the cochlea (Co) has a small gap between it and the subsequent turn, and a groove created by the secondary bony lamina (SBL) is preserved on at least the first quarter turn of the left cochlear endocast, but little else can be said about the cochlear morphology (Fig. 25). A portion of the cochlear aqueduct (CA) was located and, based on what could be reconstructed, it was a thin slit, which appears to be typical of camelids (Fig. 25B–D). Of the vestibular system, only the right anterior semicircular canal (ASC) could be partially reconstructed, and not with enough accuracy to determine an arc radius (Fig. 25). The canal appears to be relatively small compared to the cochlea, supporting the inference that the subarcuate fossa of *S. franki* may have been reduced. The lateral portion of the arc was not well preserved, so it is difficult to determine whether the canal was sigmoidal in shape.

**Fig. 26:**
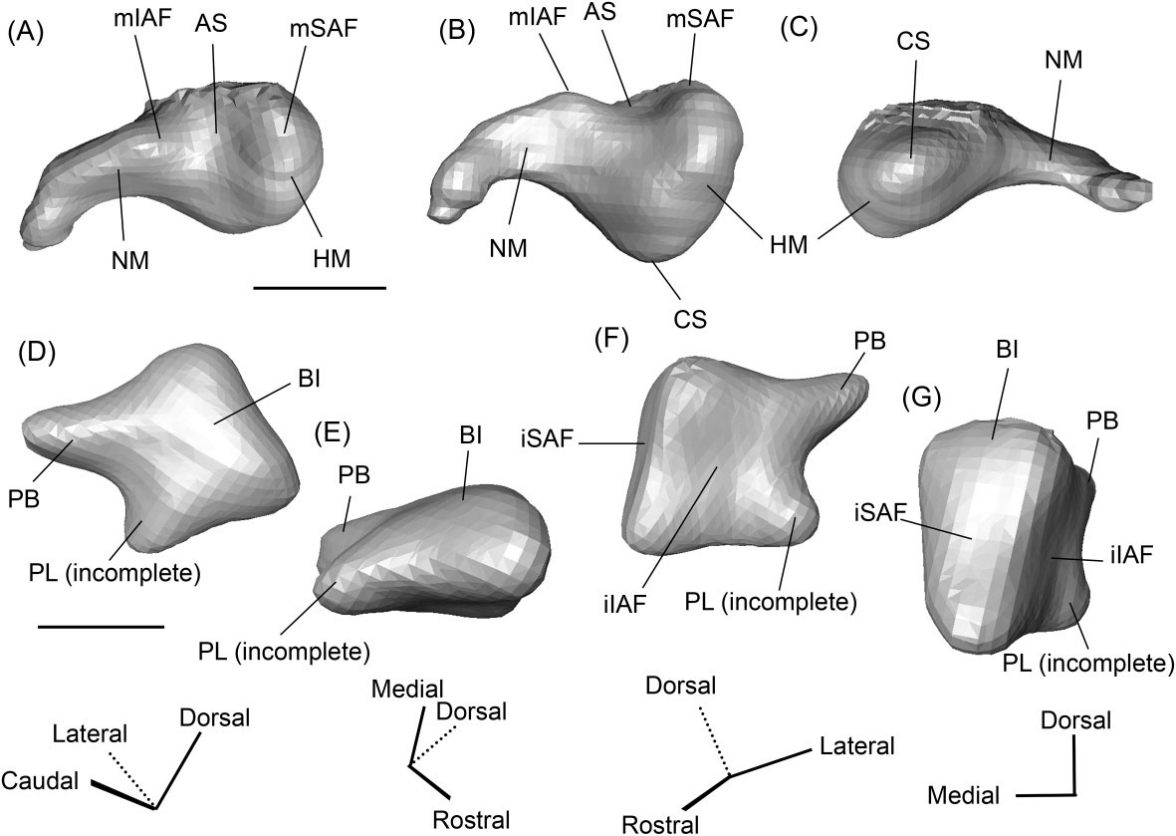
The right (mirrored) malleus (**A–C**) and incus (**D–G**) of *Stevenscamelus franki* (TMM VP-40504–149) in caudomedial (**A, D**), ventral (**B, E**), rostrolateral (**C, F**), and rostral (**G**) view. Orientations are based on *Vicugna vicugna*. Axes of orientation are shared by each view. Scale bars are shared by each bone. **Abbreviations: AS**, mallear articular surface for the incus; **BI**, body of the incus; **CS**, capitular spine; **HM**, head of the malleus; **iIAF**, incudal inferior articular facet; **iSAF**, incudal superior articular facet; **mIAF**, mallear inferior articular facet; **mSAF**, mallear superior articular facet; **NM**, neck of the malleus; **PB**, processus brevis; **PL**, processus longum. Scale bars are 1 mm.

*Ear ossicles.* The right malleus and incus are present in TMM VP 40504–149, but they are not in biological position, nor are they articulated. The head of the malleus (HM) is large and extends laterally into a rounded capitular spine (CS) (Fig. 26A–C). The articular surface for the incus (AS) is relatively shallow and broad (Fig. 26A, B). The superior articular facet (SAF) is much larger than the inferior articular facet (IAF) (Fig. 26A, B), forming an asymmetrical saddle shape. A distinct osseous lamina was not observed, and the muscular process could not be located, but this may be a result of the low scan resolution. The neck of the malleus (NM) extends laterally from the head before curving rostrally (Fig. 26A–C). The manubrium is missing.

The body of the incus (BI) is square in profile (Fig. 26D–F), and the rostral face is triangular with a narrower ventral end than dorsal end (Fig. 26G). The medial face is slightly convex while the lateral face is slightly concave. There is a shallow superior articular facet (iSAF) on the rostral face and inferior articular facet (iIAF) on the lateral face (Fig. 26F, G). While there is no prominent ridge separating the facets, they are offset from each other at about a 90° angle, making the rostral and lateral faces distinct. The processus brevis (PB) and the processus longum (PL) sit at a right angle to each other (Fig. 26D–F). The processus brevis appears to be longer than the processus longum, but we strongly suspect that the processus longum is broken. The alternatives are that 1) the processus brevis is truly longer, something that has been observed in some artiodactyls but has not been observed in camelids or 2) the longer process is the processus longum, meaning that the articular surface for the malleus is on the medial rather than lateral side of the incus. Given that both of these scenarios are unlikely, we posit that the model is simply incomplete, although we do note the possibility that *S. franki* has a morphology convergent with that of non-camelid artiodactyls.

#### Family Camelidae

##### Vicugna vicugna (UCMZ (M) 1986.307; UCMZ (M) 1986.308; UCMZ (M) 1986.309; UCMZ (M) 1986.310)

###### Petrosal

**Fig. 27:**
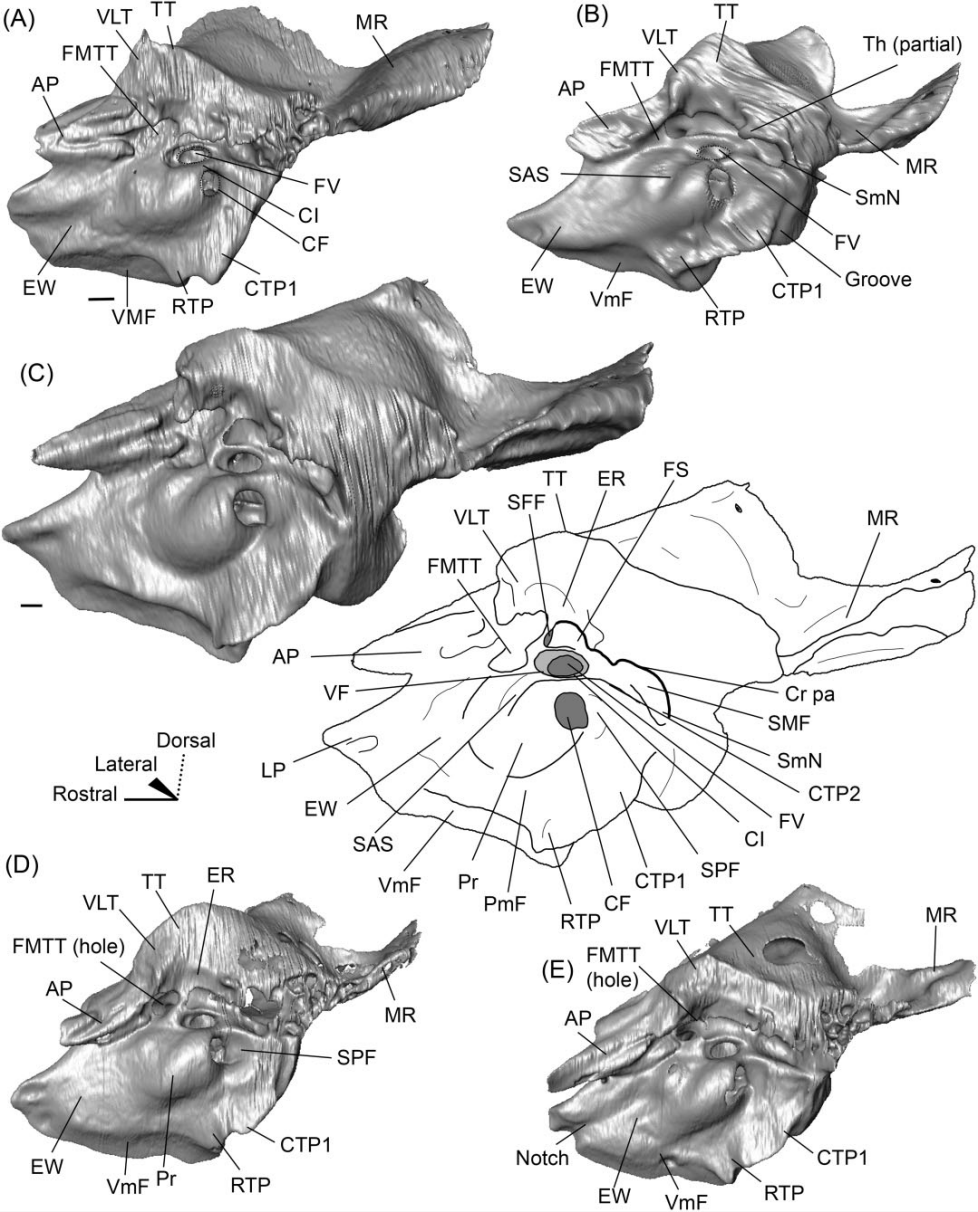
Left petrosals of *Vicugna vicugna* in tympanic view. **A**, UCMZ (M) 1986.307, adult; **B**, UNSM ZM-16921, adult; **C**, UCMZ (M) 1986.309, adult; **D**, UCMZ (M) 1986.308, juvenile; **E**, UCMZ (M) 1986.310, juvenile. Scale bar is shared by A, B, D, and E. Not all structures are labeled on all specimens. **Abbreviations: AP**, anterior process of the tegmen tympani; **CI**, crista interfenestralis; **CF**, cochlear fossula; **Cr pa**, crista parotica; **CTP1**, medial caudal tympanic process; **CTP2**, lateral caudal tympanic process; **ER**, epitympanic recess; **EW**, epitympanic wing; **FMTT**, fossa for the muscularis tensor tympani; **FS**, facial sulcus; **FV**, fenestra vestibuli; **LP**, lateral process of the epitympanic wing; **MR**, mastoid region; **PmF**, posteromedial flange; **Pr**, promontorium; **RTP**, rostral tympanic process; **SAS**, stapedial artery sulcus; **SFF**, secondary facial foramen; **SMF**, stapedial muscle fossa; **SmN**, stylomastoid notch; **SPF**, saccus posticus fossa; **Th**, tympanohyal; **TT**, tegmen tympani; **VLT**, ventrolateral tuberosity; **VF**, vestibular fossula; **VmF**, ventromedial flange. Scale bars are 2 mm.

**Fig. 28:**
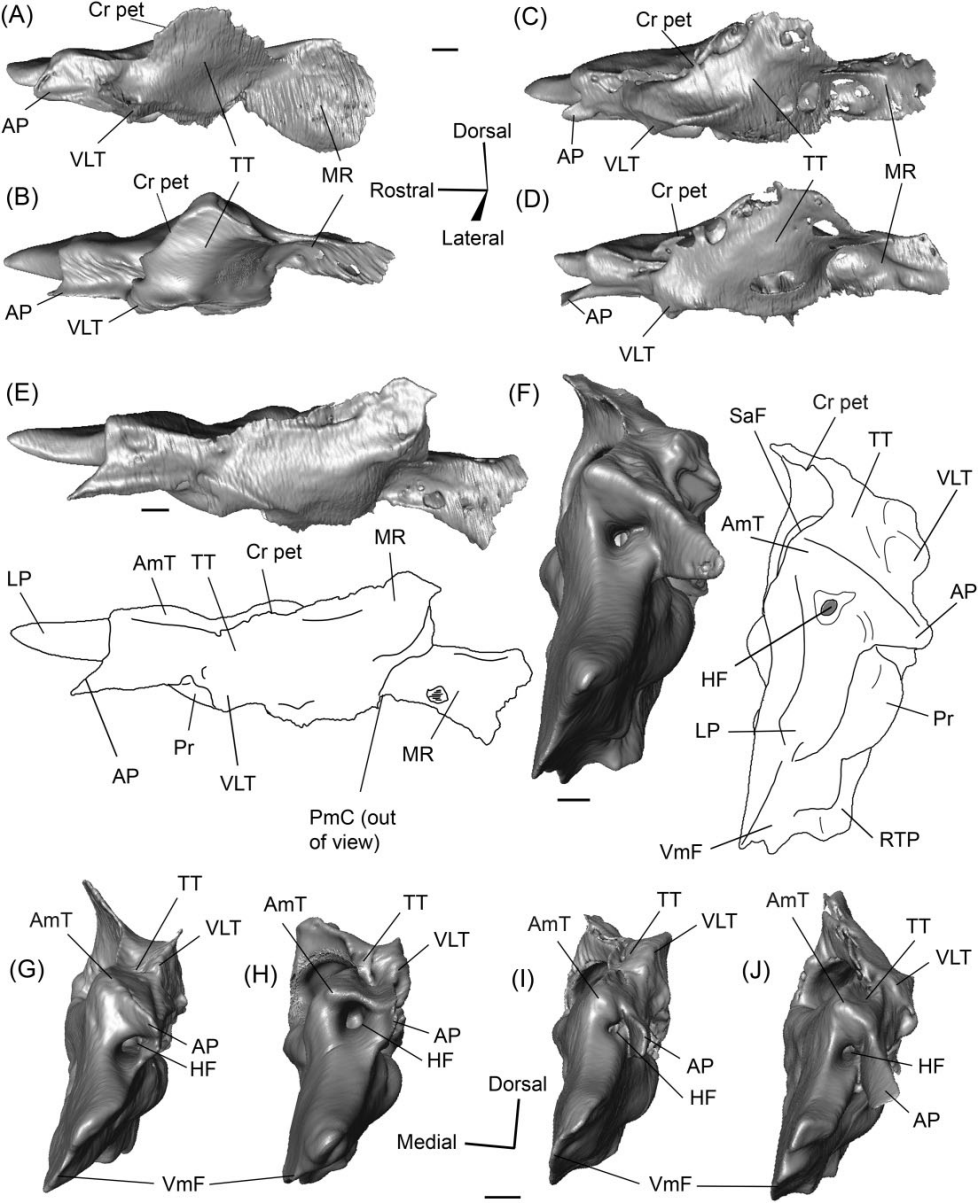
Left petrosals of *Vicugna vicugna* in dorsal **(A–E)** and rostral **(F–K)** view. **A, G**, UCMZ (M) 1986.307, adult; **B, H**, UNSM ZM-16921, adult; **C, I**, UCMZ (M) 1986.308, juvenile; **D, J**, UCMZ (M) 1986.310, juvenile; **E, F**, UCMZ (M) 1986.309, adult. Scale bars are shared between A–D, E-F, and G-J. Axes of orientation are shared by A–E and F–J. Not all structures are labeled on all specimens**. Abbreviations: AmT**, anteromedial tuberosity; **AP**, anterior process of the tegmen tympani; **Cr pet**, crista petrosa; **HF**, hiatus Fallopii; **LP**, lateral process of the epitympanic wing; **MR**, mastoid region; **PmC**, petromastoid canal; **Pr**, promontorium; **RTP**, rostral tympanic process; **SaF**, subarcuate fossa; **TT**, tegmen tympani; **VLT**, ventrolateral tuberosity; **VmF**, ventromedial flange. Scale bars are 2 mm.

**Fig. 29:**
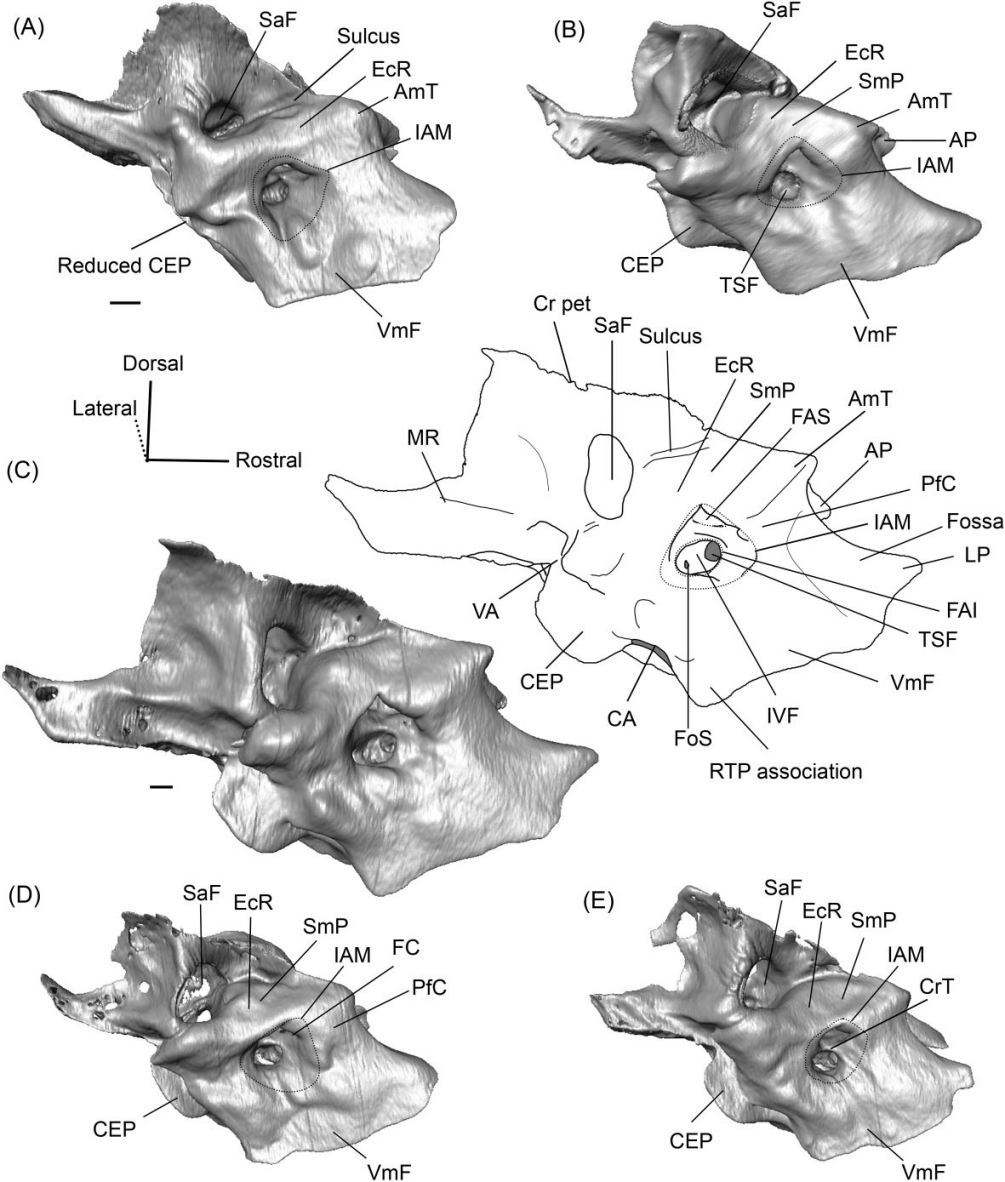
Left petrosals of *Vicugna vicugna* in endocranial view. **A**, UCMZ (M) 1986.307, adult; **B**, UNSM ZM-16921, adult; **C**, UCMZ (M) 1986.309, adult; **D**, UCMZ (M) 1986.308, juvenile; **E**, UCMZ (M) 1986.310, juvenile. Scale bar is shared by A, B, D, and E. Not all structures are labeled on all specimens. **Abbreviations**: **AmT**, anteromedial tuberosity; **AP**, anterior process of the tegmen tympani; **CA**, cochlear aqueduct; **CEP**, caudal endocranial process; **Cr pet**, crista petrosa; **CrT**, crista transversa; **EcR**, endocranial ridge; **FAI**, foramen acusticum inferius; **FAS**, foramen acusticum superius; **FC**, facial canal; **FoS**, foramen singulare; **IAM**, internal acoustic meatus; **IVA**, inferior vestibular area; **LP**, lateral process of the epitympanic wing; **PfC**, prefacial commissure; **RTP**, rostral tympanic process; **SaF**, subarcuate fossa; **SmP**, suprameatal plate; **TSF**, tractus spiralis foraminosus; **VA**, vestibular aqueduct; **VmF**, ventromedial flange. Scale bars are 2 mm.

The petrosal of *V. vicugna* sits nearly vertical in the skull. The anterior portion of the petrosal is triangular because of a large, wedge-shaped epitympanic wing (EW) (Fig. 27). The medial process of the epitympanic wing is absent in both juveniles and adults, although there is sometimes a notch in the region (“Notch” in Fig. 27E). The lateral process of the epitympanic wing (LP) extends rostral to the rest of the petrosal as a dorsoventrally compressed flange that borders the carotid foramen caudally and laterally (the carotid foramen and piriform fenestra are separate in *V. vicugna*) (Figs. 27C, 28E and 29C). There is some variation in the size and shape of the epitympanic wing, but this is not tied to ontogeny; the two juvenile specimens are in the mid-range for epitympanic wing size (Fig. 14D,E). The lateral process of the epitympanic wing smoothly intersects with the posteromedial flange (PmF), the latter of which forms a keel ventral to the promontorium (Pr) and caudal to the epitympanic wing (Fig. 14C). There is a rostral tympanic process (RTP) at the posterior extent of the flange (Figs. 14 and 15F). This process may take the form of a distinct spike (e.g., UCMZ (M) 1986.307) (Fig. 14A) or a fan continuous with the medial caudal tympanic process (CTP1) (e.g., UCMZ (M) 1986.309) (Fig. 14C), which is not unusual when both processes are present (MacPhee 1981). In these instances, the rostral tympanic process can be identified based on location: it is on the pars cochlearis and terminates at the region of the cochlear fossula (CF) (Fig. 28B, C, E). The morphology of the rostral tympanic process is also not linked to ontogeny.

A thin ventromedial flange (VmF) extends from the posteromedial flange (Figs. 27, 28F–J and 29). It can be distinguished from the posteromedial flange based on a change in orientation and its continuation beyond the rostral tympanic process. In UCMZ (M) 1986.309, the tympanic portion of the ventromedial flange is concave, a morphology similar to that of *Eotylopus* that could be interpreted as a basicapsular groove (Figs. 15F and 27C). The same pronounced concavity is not present in the other *V. vicugna* specimens—the ventromedial flange ranges in shape from slightly concave to entirely flat, and the basicapsular groove is absent (Figs. 27A, B, D, E and 28G–J).

The promontorium is situated far behind the epitympanic wing (Figs. 27C, D and 28F). It is convex where it covers the basal turn of the cochlea, but there is little rostral expansion and the area covering the subsequent turns of the cochlea is nearly flat in some specimens (e.g., UCMZ (M) 1986.308) (Fig. 27D). A faint stapedial artery sulcus (SAS) sometimes extends from the vestibular fossula (VF) (Fig. 27B, D). There is no transpromontorial sulcus. The secondary facial foramen (SFF), which sits lateral to the fenestra vestibuli, is large and directed caudally (Fig. 27C). The fenestra vestibuli (FV) is ovoid with a rostrocaudal long axis and opens ventrally (Fig. 27A). The fenestra vestibuli ratio ranges from 1.11 to 2.28, but the median is 1.73 (Supplementary Material 4); the individual with a 1.11 ratio (UCMZ (M) 1986.308) has a left fenestra vestibuli with a particularly large width. The fenestra is surrounded by a distinct vestibular fossula (VF) which, while also ovoid, has more rostral expansion and therefore a relatively longer rostrocaudal axis than the fenestra vestibuli (Fig. 27C). Unlike in *Poebrotherium*, there is no great caudal extension of the fossula. The vestibular fossula ratio ranges from 1.91 to 2.56 (Supplementary Material 4). In general, specimens with a larger fenestra vestibuli ratio also have a larger vestibular fossula ratio. The fenestra vestibuli area is between 0.63 mm^2^ to 2.20 mm^2^, but the small values from UCMZ (M) 1986.309 are unusual and the median area is 1.66 mm^2^ (Supplementary Material 4). As in *Poebrotherium*, the size of the fenestra vestibuli does not appear to be linked to ontogeny.

There is a flat crista interfenestralis (CI) separating the vestibular fossula and the aperture of the cochlear fossula (CF) (Fig. 27A, C). The width of the crista interfenestralis is approximately the same as the width of the vestibular fossula (Supplementary Material 4). Like the secondary facial foramen, it is directed caudally. In UCMZ (M) 1986.308, a wide saccus posticus fossa (SPF) extends caudally from the cochlear fossula (Fig. 27D). This fossa is not always so distinct in other individuals, but it is consistently present as an indentation directly caudal to the aperture of the cochlear fossula (Fig. 27C).

The fossa for the muscularis tensor tympani (FMTT) is triangular and deeply recessed in the medial wall of the tegmen tympani (Fig. 27). The caudal portion of the fossa is barely visible in tympanic view. Although the amount of rostral extension varies among the specimens, the rostral portion of the fossa is located medial to the tegmen tympani and is always visible in tympanic view. The bone in this region is thin in both juveniles and adults, and in both juveniles, there is a hole in the fossa that exposes the facial nerve canal (Fig. 27D, E). The facial sulcus (FS) extends caudally from the secondary facial foramen, just behind the fossa for the muscularis tensor tympani (Fig. 27C). It is a wide channel overhung by the crista parotica (Cr pa) (Fig. 27C). The stapedial muscle fossa (SMF) is wide and deep, forming a pit just rostral to the stylomastoid notch (SmN) (Fig. 27B, C). The tympanohyal (Th) is frequently retained, creating the ventral border of the stylomastoid foramen (Fig. 27B). The medial portion of the caudal tympanic process (CTP1) is present, but it often fuses with the rostral tympanic process to form a fan-like structure (Fig. 27). The lateral portion of the caudal tympanic process (CTP2) is rostrally confluent with the crista interfenestralis and forms a ridge between the aperture of the cochlear fossula and the stapedial muscle fossa, extending caudally to intersect with the flared medial caudal tympanic process (Fig. 27C). On some specimens (e.g., UCMZ (M) 1986.308; UNSM ZM-16921), there is a groove (“Groove” in Fig. 27B) dorsocaudal to the medial caudal tympanic process that looks to have carried a soft tissue structure. This is presumably the groove that O’Leary (2010) considered to be an extension of the facial sulcus, but only in one specimen, the potential hybrid (UNSM ZM-16921), does the groove appear to directly continue from the stylomastoid notch. The mastoid region (MR) is narrow but long, projecting caudally as a thin wedge (Figs. 27, 28A–E and 29C). It is more tightly sutured to the surrounding bones in the adults than in the juveniles, but the relative size of the mastoid region does not appear to be associated with age.

The tegmen tympani (TT) is moderately inflated with a transverse width approximately half that of the promontorium, but there is notable variation in size and shape between specimens (Figs. 27 and 28). The tympanic portion of the bone typically forms a flat wall that is at a right angle to the dorsolateral face of the petrosal (Figs. 27 and 28F–J). However, in UCMZ (M) 1986.308, this area is expanded and protrudes laterally, forming a prominent ridge that obscures the dorsolateral face in tympanic view (Fig. 27D). In this specimen, the tegmen tympani is as wide as the promontorium, making it hyperinflated (Geisler and Luo 1998). A similar but less pronounced morphology is present in UNSM ZM-16921 (Fig. 27B). In all specimens, there is a small ventrolateral tuberosity (VLT) that forms a squared-off knob just rostral to the epitympanic recess (Figs. 27 and 28). The epitympanic recess (ER) is long and shallow without a fossa incudis (Fig. 27C, D). In front of the ventrolateral tuberosity, there is a large spike-shaped anterior process of the tegmen tympani (AP) that begins at the fossa for the muscularis tensor tympani and extends rostral to the promontorium (Figs. 27–29). As with the area around the ventrolateral tuberosity, the anterior process exhibits variation, with some processes terminating far caudal to the lateral process of the epitympanic wing (e.g., UCMZ (M) 1986. 309) (Figs. 27C and 28E, F) and others extending to the tip of the lateral process (e.g., UCMZ (M) 1986.310) (Figs. 27D and 28C–I). Also, in the potential hybrid UNSM ZM-16921, the lateral border of the anterior process is defined by a large ridge (Fig. 28B). In the other specimens, no such ridge is present, and the dorsolateral side of the anterior process is visible in tympanic view. The dorsolateral surface of the anterior process is flat and broad. A subcircular hiatus Fallopii (HF) opens on the rostral face of the petrosal, medial to the anterior process (Fig. 28F–J).

Endocranially, a long crista petrosa (Cr pet) dorsally borders the pars canalicularis (Figs. 28A–F and 29C). There is no other crest in the region and no indication of the superior petrosal sinus and sigmoid sinus sulci. The subarcuate fossa (SaF) is large, spherical, and directed rostrally (Figs. 7F and 29). The bone forming the lateral wall of the subarcuate fossa is very thin. It is backed by the auditory bulla and, in the juveniles, may still be in the process of ossification based on its low density, although this could also be a result of scanning parameters. This phenomenon was not observed in the other taxa in this study. The overall subarcuate fossa morphology is shared by juveniles and adults, and the relative size of the subarcuate fossa does not appear to vary with ontogeny (Fig. 29). It is bounded by all three semicircular canals and caudally extends slightly beyond the arch of the posterior semicircular canal (Fig. 7F). There is a petromastoid canal (PmC) that travels caudally from the fossa to the mastoid region, transmitting the subarcuate artery in life (Fig. 28E). One adult (UCMZ (M) 1986.307) has a greatly reduced subarcuate fossa that forms a distorted cone rather than a sphere (Figs. 7G and 29A). To our knowledge, this morphology has not previously been reported in laminins. Aside from UCMZ (M) 1986.307, the subarcuate fossa has a ventrolateral fossula that that extends through the arch of the lateral semicircular canal (Fig. 7F). This differs from the condition in early camelids where the fossula extends through the arch of the posterior semicircular canal (Fig. 7C–E). While UCMZ (M) 1986.307 lacks association with the semicircular canal, the fossa does have a laterally expanded portion (Fig. 7G).

The area of the petrosal rostrolateral to the subarcuate fossa lacks the depression commonly found in early camelids. Rather, there is a rostrally trending sulcus (“Sulcus” in Fig. 29A–C) that runs along the dorsolateral border of a prominent endocranial ridge (EcR) (Fig. 29). This sulcus is in the same location as the one observed in the *Poebrotherium* sp. specimen FMNH PM 14560, but unlike in FMNH PM 14560, it does not connect to the superior petrosal sinus sulcus. It is possible that the superior petrosal sinus of *V. vicugna* is not carried on the petrosal, but rather travels adjacent to it, and the sulcus in question is the sulcus for the subarcuate vein, but this cannot be confirmed without further investigation. The endocranial ridge is often flattened, but it is rounded in UCMZ (M) 1986.307 and, contrasting with the other specimens, UCMZ (M) 1986.307 lacks a suprameatal plate (SmP) (Fig. 29A). The endocranial ridge intersects with the anteromedial tuberosity (AmT), the latter of which is large and rounded, sometimes terminating in a blunt point (Fig. 29). The anterior process of the tegmen tympani is often visible adjacent to the anteromedial tuberosity (Fig. 29B, C), and the hiatus Fallopii is surrounded by the two structures (Fig. 28F–J). The differences present in UCMZ (M) 1986.307 appear to be individual variation.

The internal acoustic meatus (IAM) is somewhat variable in shape, but it is always roofed caudally by the endocranial ridge and dorsally by the anteromedial tuberosity (Fig. 29). The rostral (and sometimes ventral) border is flush with the surrounding bone. This is once again most likely individual rather than ontogenetic variation: one juvenile (UCMZ (M) 1986.308) most closely resembles two of the adults (Fig. 29B–D), while the other juvenile (UCMZ (M) 1986.310) has a morphology distinct from the other specimens, as does the remaining adult (Fig. 29A–E). In all specimens, the foramen acusticum superius (FAS) and foramen acusticum inferius (FAI) are more-or-less dorsoventrally in line, separated by the crista transversa (CrT) (Fig. 29C–E). The facial canal (FC) extends from the internal rostral border of the foramen acusticum superius (Fig. 29D), and the superior vestibular area is a large depression at the caudal portion of the foramen, but it cannot be seen in endocranial view. A portion of the tractus spiralis foraminosus (TSF) is visible within the foramen acusticum inferius (Fig. 29C). The foramen singulare (FoS) sits at the caudal end of the foramen acusticum inferius in a slight depression, adjacent to the inferior vestibular area (IVA) (Fig. 29C). The internal acoustic meatus occupies a relatively small portion of the endocranial pars cochlearis because the area rostral to the meatus and the ventromedial flange is greatly expanded. The ventromedial flange deepens caudally in the area of the rostral tympanic process (“RTP association” in Fig. 29C), giving the ventral border of the petrosal a triangular appearance (Fig. 16). The area of the prefacial commissure (PfC) dorsolateral to the internal acoustic meatus lacks a prefacial commissure fossa (Fig. 29C).

The cochlear aqueduct (CA) exits caudomedial to the internal acoustic meatus (Fig. 29C). The exit, which is associated with the perilymphatic sac, is often internally connected to the exit for the vestibular aqueduct (VA), the latter of which is ventrocaudal to the subarcuate fossa at the posterior end of the endocranial ridge (Fig. 29C). The exit for the vestibular aqueduct contained the endolymphatic sac in life. It has two openings, one connected directly to the vestibular aqueduct, and the other connected to a canal that extends between the areas of the endolymphatic sac and the perilymphatic sac, either joining the two structures internally or ending in a blind-ended tube. A caudal endocranial process (CEP) is consistently present just posterior to the exit of the cochlear aqueduct and extends to the level of the vestibular aqueduct (Fig. 29). In most of the specimens, the caudal endocranial process forms a large wing, but in UCMZ (M) 1986.307 it is reduced to a small flange that only borders the exit of the vestibular aqueduct (Fig. 29A).

One adult—UNSM ZM-16921—has notable bilateral asymmetry on the endocranial face of its petrosals. The left petrosal has the standard morphology for *V. vicugna*, with a wide endocranial ridge that terminates in a flat suprameatal plate and large anteromedial tuberosity that, along with the anterior process of the tegmen tympani, borders the hiatus Fallopii (Figs. 28H and 29B). Together, the endocranial ridge and the anteromedial tuberosity roof the foramen acusticum superius of the internal acoustic meatus. The foramen acusticum superius is internally connected to the hiatus Fallopii because the greater petrosal nerve is transmitted through this passage. The right petrosal lacks an enclosed foramen acusticum superius, leaving the passage for the greater petrosal nerve exposed (Supplementary Material 3, Fig. S4). This means that the endocranial aspect of the foramen acusticum superius and the hiatus Fallopii are notches rather than foramina, and there is no suprameatal plate or anteromedial tuberosity.

**Fig. 30:**
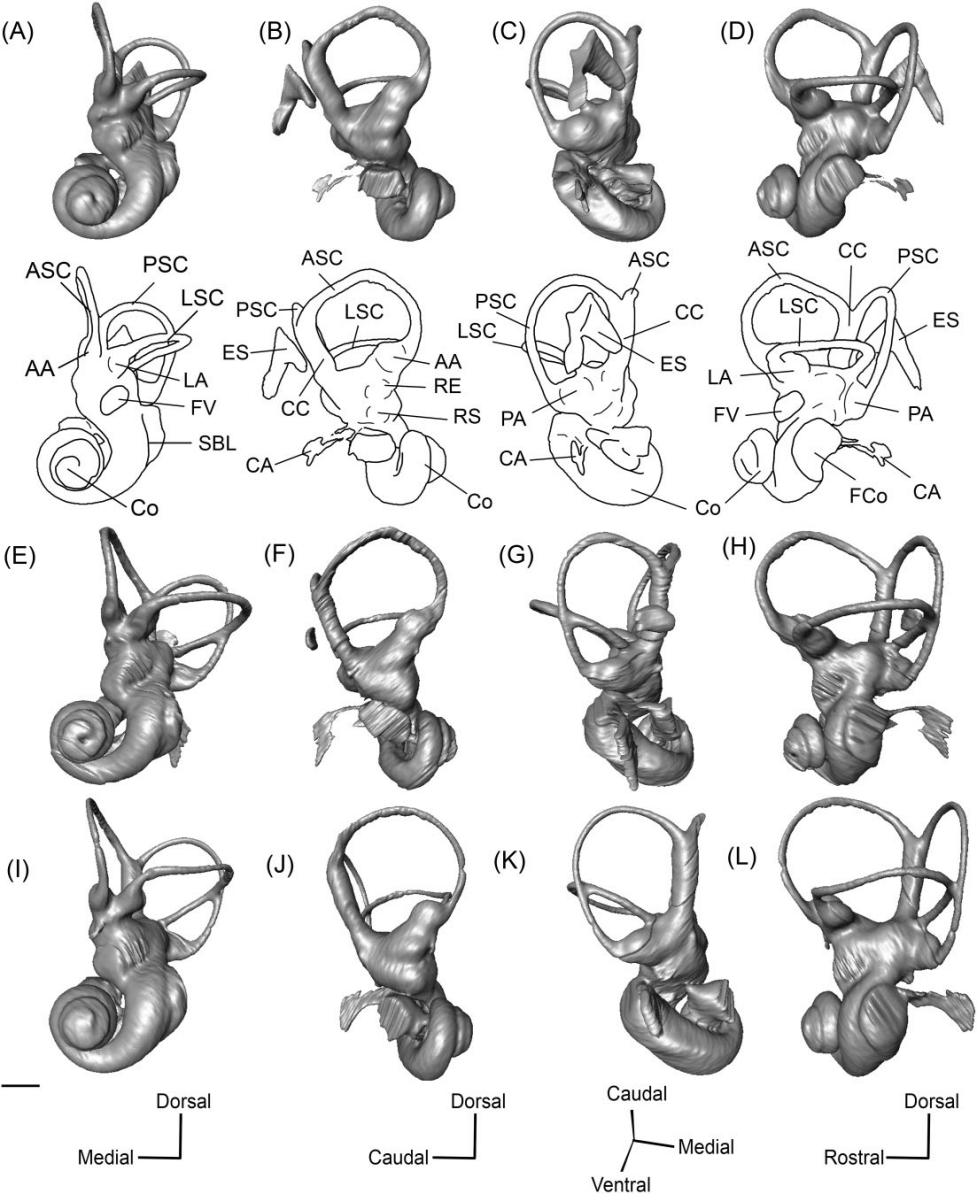
The left bony labyrinths of *Vicugna vicugna* in rostral (**A**), endocranial (**B**), ventromedial (**C**), and tympanic (**D**) view. **A–D**, UCMZ (M) 1986.307, adult; **E–H**, UCMZ (M) 1986.309, adult; and **I-L**, UCMZ (M) 1986.310, juvenile. **Abbreviations: AA**, anterior ampulla; **ASC**, anterior semicircular canal; **CA**, cochlear aqueduct; **CC**, common crus; **Co**, cochlea; **ES**, endolymphatic sac; **FCo**, fenestra cochleae; **FV**, fenestra vestibuli; **LA**, lateral ampulla; **LSC**, lateral semicircular canal; **PA**, posterior ampulla; **PSC**, posterior semicircular canal; **RE**, recessus ellipticus; **RS**, recessus sphaericus; **SBL**, secondary bony lamina. Scale bar is 2 mm.

*Bony labyrinth.* The *V. vicugna* cochlea (Co) has 2.50 to 3.25 turns and is between 23 mm to 30 mm long (Fig. 30) (Supplementary Material 4). The juveniles are not any shorter than the adults. The cochlear aspect ratio (0.48–0.56) is also not impacted by late-stage ontogeny (Supplementary Material 4). There is a wide gap between the basal turn and the subsequent turns, but the gap narrows halfway around the basal turn, and the coils become appressed to each other before the end of the turn. The secondary bony lamina (SBL) left a wide groove on the outer wall of the cochlear endocast that extends from the fenestra cochleae (FCo) to at least halfway up the basal coil (Fig. 30A, D), and the primary bony lamina left a deep groove along most of the length of the inner wall.

The cochlear aqueduct (CA) is a long, narrow channel (i.e., slit) that terminates in the perilymphatic sac caudal to the posterior semicircular canal (Fig. 30B–D). The exit of the cochlear aqueduct on the petrosal can be seen in Fig. 29. The full path of the vestibular aqueduct was not reconstructed, but the structure originates just anterior to the common crus (CC) (Fig. 30B–D). It extends only a short distance before opening into the endolymphatic sac (ES), the latter of which begins far below the apex of the common crus (Fig. 30B–D). Rostrally, the recessus ellipticus (RE) forms a large bulge that is approximately the same size as the anterior ampulla (Fig. 30B). The recessus sphaericus (RS) is more subdued, but it still protrudes enough to be distinct from the surrounding area (Fig. 30B). The size of the recesses does not vary with ontogeny (Fig. 30B, F, J).

All ampullae are well defined. The anterior (AP) and posterior ampullae (PA) are approximately equal in size, while the lateral ampulla (LA) is slightly smaller (Fig. 30). This corresponds to the sizes of the semicircular canals, in which the lateral semicircular canal (LSC) is noticeably smaller than the other two (Fig. 30) (Supplementary Material 4). The anterior semicircular canal (ASC) is consistently a little larger than the posterior semicircular canal (PSC), and it extends slightly more dorsally than the latter (Fig. 30). The length of the common crus is, on average, slightly more than 60% the height of the anterior semicircular canal (Supplementary Material 4). The specimen with the reduced subarcuate fossa (UCMZ (M) 1986.307) also has the smallest semicircular canals and common crus (Supplementary Material 4). As in the other described taxa, the anterior semicircular canal has a sigmoidal shape while the posterior and lateral canals remain predominantly in a single plane. Angles between the semicircular canals range from 79° to 103°, although not within the same specimen (Supplementary Material 4). There is no pattern as to which canals have the largest or smallest angles, and the size of the angles is not connected to the age of the individual. The cochlea and lateral semicircular canal meet at a 33° to 58° angle, without a difference between the juveniles and adults (Supplementary Material 4).

**Fig. 31:**
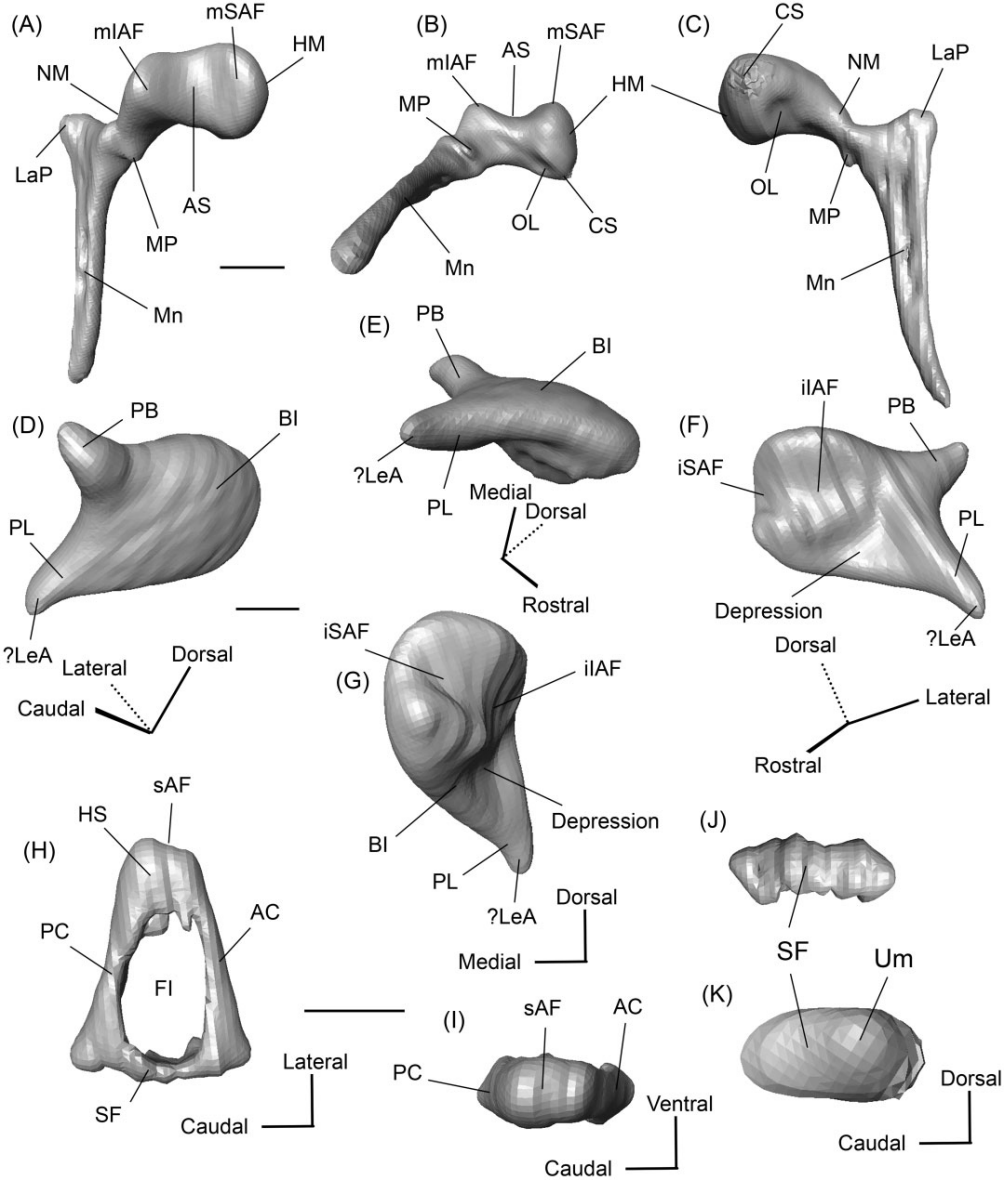
The left malleus of *Vicugna vicugna* (UCMZ (M) 1986.309) (**A–C**), the left incus of *V. vicugna* (UCMZ (M) 1986.308) (**D–G**), and the left stapes of *V. vicugna* (UCMZ (M) 1986.308) (**H–J**) and *V. vicugna* (UNSM ZM-16921) (**K**) in caudomedial (**A, D**), ventral (**B, E**), rostrolateral (**C, F**), rostral (**G**), dorsal (**H**), lateral (**I**), and medial (**J-K**) view. Axes of orientation are shared by each column. Scale bars are shared by each bone. **Abbreviations: AC**, anterior crus; **AS**, mallear articular surface for the incus; **BI**, body of the incus; **CS**, capitular spine; **FI**, foramen intercrurale; **HM**, head of the malleus; **HS**, head of the stapes; **iIAF**, incudal inferior articular facet; **iSAF**, incudal superior articular facet; **LaP**, lateral process of the malleus; **LeA**, lenticular apophysis; **mIAF**, mallear inferior articular facet; **Mn**, manubrium; **MP**, muscular process; **mSAF**, mallear superior articular facet; **NM**, neck of the malleus; **OL**, osseous lamina; **PB**, processus brevis; **PC**, posterior crus; **PL**, processus longum; **sAF**, stapedial articular facet; **SF**, stapedial footplate; **Um**, umbo. Scale bars are 1 mm.

*Ear ossicles.* One juvenile and two adult *V. vicugna* specimens have ear ossicles (Supplementary Material 5), including one specimen with the ossicles close to biological position (Supplementary Material 3, Fig. S5), allowing for a description of all three bones. There are no notable differences in the morphology of the bones between juveniles and adults aside from the amount of ossification. The head of the malleus (HM) is dominated by the articular surface for the incus (AS), which forms a wide and shallow trough on the caudal face of the head (Fig. 31A–C). The superior and inferior articular facets (SAF; IAF) are broad protuberances, both of which appear to extend the entire length of the mallear head and create a characteristic saddle shape. The inferior articular facet is shorter than the superior articular facet, but the two facets are approximately the same height. The head does not greatly project rostrally, but there is a capitular spine (CS) (Fig. 31B, C). The osseous lamina (OS) is a shallow depression ventrolateral to the capitular spine (Fig. 31B, C). A small muscular process (MP), which sits lateral to the osseous lamina (OS), is consistently present in the specimens. The neck of the malleus (NM) is short and thick (Fig. 31A–C). There is a distinct lateral process (LaP) at the junction between the neck and the manubrium (Mn), the latter two of which lie in the same plane (Fig. 31A–C). The manubrium forms an obtuse angle with the neck and is directed ventromedially. It is triangular in shape and has a uniform thickness for its whole length, and the distal tip is slightly curved. The center of the manubrium is not yet ossified in the juvenile (UCMZ (M) 1986.308), a condition that appears to continue to a lesser degree in the adults.

The body of the incus (BI) in *V. vicugna* is longer than it is wide, resulting in a rectangular appearance (Fig. 31D–G). The dorsal side is broad and convex, whereas the ventral side is quite narrow. While the superior articular facet for the malleus (iSAF) is on the rostral side of the head, it slopes caudolaterally, resulting in the rostral face being wedge-shaped (Fig. 31F, G). There is no clear ridge separating the superior articular facet from the inferior articular facet (iIAF), the latter of which occupies the lateral face of the incudal body (Fig. 31F, G). Both the superior and inferior facets are shallow and broad, which matches the morphology of the mallear head (Fig. 31A, B). There is a distinct depression (“Depression” in Fig. 31F, G) on the lateral face, ventral to the inferior articular facet. This contributes to the incus appearing very narrow, almost flattened, in some views. The processus brevis (PB) and the processus longum (PL) are at an obtuse angle (Fig. 31D–F). Although the relative lengths of the two processes vary, even bilaterally within the same specimen (e.g., UCMZ (M) 1986.308), the processus longum is consistently longer than the processus brevis. The processus longum narrows distally and there may be some indication of the lenticular apophysis (LeA), but the structure is not reconstructed in high resolution (Fig. 31D–G).

The stapes is an elongate trapezoid with an ovate foramen intercrurale (FI) extending from the stapedial footplate (SF) to the head of the stapes (HS) (Fig. 31H). There is no evidence of a processus muscularis stapedius. Although the anterior and posterior crura (AC; PC) are the same length and width, the head of the stapes is not uniform in thickness in UCMZ (M) 1986.308 (Fig. 31H). It is caudally expanded bilaterally, resulting in a sloped articular facet (sAF) (Fig. 31H, I). This tilt is also present in UNSM ZM-16921, but to a lesser degree, indicating that it may be subject to intraspecific or ontogenetic variation. The articular facet is quite small compared to the stapedial footplate. The footplate itself is ovate with a narrow rim and slightly tapered caudal end (Fig. 31J, K). A large umbo (Um) sits rostrally (Fig. 31K). The area of the left UNSM ZM-16921 stapedial footplate could be measured with accuracy, allowing for a comparison between the size of the stapedial footplate and the fenestra vestibuli. The stapedial footplate is 1.35 mm^2^ while the fenestra vestibuli is 1.87 mm^2^ (Supplementary Material 4), indicating that the stapedial footplate is smaller than the associated fenestra vestibuli.

## Discussion

The data presented herein allow for comparisons between oromerycids, early diverging camelids, and the extant vicuña, along with an evaluation of late-stage ontogenetic changes in the otic region of tylopods.

### Ontogenetic changes

Based on the sample of juveniles (N = 5) and adults (N = 6) referred to *Poebrotherium* and *Vicugna vicugna* described in this paper (Table 1), the petrosal, bony labyrinth, and ossicular chain of camelids are morphologically static after M2 begins to erupt. In *V. vicugna*, the manubrium of the malleus appears to be less ossified in the juvenile, but this does not change the shape of the bone. Also, in *V. vicugna*, the mastoid region of the petrosal becomes more tightly sutured to the surrounding bones with age, but the same is not the case in *Poebrotherium*, possibly because the mastoid region is less extensive. These findings are in line with those of Mennecart and Costeur (2016b) and Costeur et al. (2017) for ruminants. Indeed, in ruminants, the bony labyrinth has finished ossifying prior to birth, as in humans. Only the cochlear and vestibular aqueducts (and the associated perilymphatic and endolymphatic sacs) continue to lengthen after birth as bone accretion occurs in the petrosal (Costeur et al. 2017). The length of the vestibular aqueduct could not be measured for most specimens, but the cochlear aqueduct does not appear to differ in length between the juveniles and adults of either *Poebrotherium* or *Vicugna* (Supplementary Material 4), suggesting that bone accretion in the area has ceased by the time of M2 eruption. Basicranial sutures were not fused in the juvenile *V. vicugna*, but it is not unusual for artiodactyls to fuse sutures later in life, if at all (Bärmann and Sánchez-Villagra 2012), so this is not necessarily an indication of continued growth in the region. As noted above, the mastoid region does eventually become more tightly fused to the surrounding bones in *Vicugna*. Given the lack of age-based changes in *Poebrotherium* and *Vicugna*, it is reasonable to consider the morphology observed in the juvenile *Eotylopus* cf. *E. reedi* to reflect that of the adult.

### Morphological variation among taxa

#### Protylopus

##### Petrosal

Of the taxa included in this study, the petrosal of *Protylopus* is by far the most morphologically distinct: it is rostrocaudally compressed, has a medial process of the epitympanic wing, lacks a ventromedial flange, carries a medially located basicapsular groove, lacks a caudal endocranial process, and seemingly lacks a rostral tympanic process. Given the morphology of early artiodactyls, the only potentially derived features shared between *Protylopus* and the other taxa in this study are the shape of the anterior process of the tegmen tympani, the presence of a suprameatal plate, and a groove extending rostrally from the fossa for the muscularis tensor tympani (Orliac and O’Leary 2014; Robson and Theodor 2025). Based on the petrosal alone, it is unlikely that a close relationship between *Protylopus* and *Eotylopus* would be inferred.

*Protylopus* retains some features shared with early artiodactyls, including a (reduced) tegmen tympani canal, a medial process of the epitympanic wing, a broad fossa for the muscularis tensor tympani, a small anterior process closely associated with the ventrolateral tuberosity, and a spherical subarcuate fossa that is not expanded through any of the semicircular canals (Orliac and O’Leary 2014; Robson and Theodor 2025). It also has some derived aspects such as the loss of a tegmen tympani fossa and a convex promontorium, but these features occur many times in artiodactyls (O’Leary 2010) and are unlikely to indicate a close relationship to any particular clade. Indeed, the petrosal of *Protylopus* does not closely resemble that of any other described artiodactyl. The rostrocaudal compression and large posteromedial flange are reminiscent of ?*Helohyus* and *Gobiohyus* (Orliac and O’Leary 2014), but the rest of the morphology is fairly dissimilar, particularly on the endocranial face.

Given the hypothesis that *Bunomeryx* might be an early tylopod (Norris 1999), it would be reasonable to expect the petrosal of *Protylopus* to resemble that of *Bunomeryx*, but this is also not the case. The petrosal of *Bunomeryx* is more rostrocaudally elongate, has a smaller lateral process of the epitympanic wing, a much smaller crista petrosa and posteromedial flange, retains a tegmen tympani fossa, lacks the pronounced dorsolateral expansion of the tegmen tympani, and has an incipient ventromedial flange (Norris 1999; Robson and Theodor 2025). The similarities between *Protylopus* and *Bunomeryx*, such as the spherical subarcuate fossa lacking a ventral fossula, are likely plesiomorphic (Orliac and O’Leary 2014; Robson and Theodor 2025).

The petrosal of *Protylopus* also does not resemble that of protoceratids, another endemic North American family of purported tylopods (Joeckel and Stavas 1996; Norris 2000; Robson et al. 2021, 2022). The early-diverging protoceratids *Leptoreodon* and *Leptotragulus* have a more elongate petrosal, a reduced and conical subarcuate fossa (even more so than in SDSNH 40812), and lack a medial process of the epitympanic wing (Norris 2000; Robson et al. 2022). The only notable similarity is the large posteromedial flange (incorrectly identified as a rostral tympanic process by Norris 2000 and Robson et al 2022), but a similarly enlarged flange is present in other artiodactyls that are unlikely to be closely related to oromerycids (Orliac et al. 2017). The later branching protoceratid *Protoceras* has an even more dissimilar morphology, including the complete loss of the subarcuate fossa and the seemingly secondary acquisition of a large tegmen tympani fossa (Robson et al. 2021). While the petrosal of SDSNH 40812 was not as well preserved as that of SDSNH 60369, aside from differences in the subarcuate fossa, there are enough similarities to conclude that the morphology of SDSNH 60369 is representative of the genus and not the result of an aberrant individual. Overall, the petrosal morphology of *Protylopus* raises more questions than it answers in terms of evolutionary relationships.

#### Bony labyrinth

The bony labyrinth of *Protylopus* is similar to that of *Eotylopus* and the camelids. The cochlea makes between 2 to 3 turns, the angle between the cochlea and the lateral semicircular canal is more than 20°, the aspect ratio is greater than 0.55, and there is no secondary common crus, all of which are derived conditions in artiodactyls (Orliac et al. 2012; Ekdale 2013; Robson and Theodor 2025). Furthermore, like the other taxa in this study, the anterior semicircular canal is the largest of the three canals and the cochlear aqueduct is a thin slit rather than a round tube. The only notable difference is that the cochlear aqueduct of *Protylopus* is short, which is potentially plesiomorphic (Orliac et al. 2012; Robson and Theodor 2025) and supports the relatively basal position of *Protylopus*. The similarities between the bony labyrinth of *Protylopus* and the other taxa in this study suggest that the standard tylopod bony labyrinth morphology evolved very early in the suborder. Of note, the bony labyrinth of the protoceratid *Leptoreodon* is also quite similar, including a high aspect ratio and the lack of a secondary common crus (Robson et al. 2022). Whether this is a result of convergence or shared ancestry remains open to debate.

Despite the disparity between the petrosal of *Protylopus* and *Eotylopus*, it is highly unlikely that one of the two taxa is not an oromerycid: both taxa have the bifurcate protocone and medial notch on m3 that are characteristic of the family (Wortman 1898; Matthew 1910; Prothero 1998a). It is far more likely that *Protylopus* is simply an earlier diverging oromerycid. This is supported by the auditory bulla of *Protylopus*, which is reported to be small and hollow (Scott 1899), unlike the cancellous bulla of camelids and *Eotylopus* (Wortman 1898), and the relatively robust postcranial bones of *Protylopus* that are reduced in *Eotylopus* (Matthew 1910). Given this, the petrosal appears to be yet another basal feature of *Protylopus*, illustrating the potential for morphological variation within a family.

#### Eotylopus

##### Petrosal and bony labyrinth

The otic region of *Eotylopus* exhibits what is almost a textbook example of a transitional morphology. The petrosal has several derived features shared with camelids, including a rostral tympanic process, a distinct ventromedial flange that is laterally confluent with the posteromedial flange, a smaller fossa for the muscularis tensor tympani, a depression lateral to the subarcuate fossa, and the lack of a medial process of the epitympanic wing (Supplementary Material 3, Fig. S6). Yet, there are other ways in which the *Eotylopus* petrosal is plesiomorphic, such as the lack of a caudal endocranial process, a medial caudal tympanic process without a flange, a squared-off ventrolateral tuberosity, and the small size of the ventromedial flange (Supplementary Material 3, Fig. S6). *Eotylopus* also does not have the expanded epitympanic wing typical of camelids, nor a vestibular fossula distinct from the fenestra vestibuli and a ventral fossula in the subarcuate fossa (Fig. 7B–F; Supplementary Material 3, Fig. S6). However, the subarcuate fossa is relatively large and extends above the arch of the anterior semicircular canal, indicating some expansion of the structure. The hiatus Fallopii of *Eotylopus* is on the rostral surface, although it is placed more laterally than in *Protylopus*. The hiatus Fallopii of *Paratylopus* and *Vicugna* is also located rostrally, but the hiatus Fallopii of *Poebrotherium* is located laterally, so the polarity of this feature in camelids is unclear (see Fig. 1 for hypothesized relationships).

The bony labyrinth of *Eotylopus* is indistinguishable from that of early camelids and, while the specimen studied did not retain its auditory bullae, it has been reported that, like camelids, the bulla of *Eotylopus* contains cancellous bone (Matthew 1910). All in all, the otic region morphology of *Eotylopus* is exactly what would be expected of a taxon closely related to camelids. It is far more similar to camelids than is the otic region of protoceratids, *Protylopus*, and homacodontids (Joeckel and Stavas 1996; Norris 1999, 2000; Orliac and O’Leary 2014; Robson et al. 2021, 2022; Robson and Theodor 2025), supporting the hypothesis that oromerycids are potentially the sister family to camelids.

#### *Early camelids* (Poebrotherium *spp.*, Paratylopus primaevus, Stevenscamelus franki)

The petrosal, bony labyrinth, and ear ossicle morphology of early camelids is broadly conserved among the taxa in this study. The morphology of the *Poebrotherium* specimens described here concurs with the descriptions provided by Whitmore (1953) and O’Leary (2010), aside from one important exception: O’Leary (2010) did not consider *Poebrotherium* to have a rostral tympanic process. A rostral tympanic process is consistently present in the specimens examined for this study and is one of the significant features linking *Eotylopus* to the camelids. Also of note, the internal acoustic meatus of early camelids appears to be very small, something that was observed in *Bunomeryx* and suggested to be a tylopod synapomorphy (Norris 1999) but is not present in *Protylopus* and *Eotylopus.* However, comparative measurements are necessary to establish the nature of this apparent difference.

#### Poebrotherium *spp*.

The petrosal morphology of *Po. eximium* and *Po. wilsoni* is seemingly indistinguishable, aside from possibly a smaller fenestra vestibuli area in *Po. eximium*, supporting the referral of both species to the same genus. The *Po. wilsoni* cochlea has more turns and a potentially lower aspect ratio than the *Po. eximium* cochlea but given the limited number of specimens, we do not consider these differences to definitively indicate interspecific variation. That being said, based on the fenestra vestibuli and bony labyrinth alone, the unusual *Poebrotherium* specimen FMNH PM 14560 would be placed with *Po. eximium* despite it being geologically younger than the youngest known *Po. eximium* specimens (Prothero and Emry 2004). However, given the morphological differences between the petrosal of FMNH PM 14560 and specimens of *Po. eximium* and *Po. wilsoni*, we feel that our choice not to refer it to a species is justified.

#### Paratylopus primaevus

The petrosal of *Pa. primaevus* is distinct from both species of *Poebrotherium*, most notably in the morphology of the anterior process of the tegmen tympani. The anterior process of *Pa. primaevus* is much larger than that of *Poebrotherium*, occupying a greater portion of the tympanic surface and contributing to the dorsolateral border of the petrosal. The hiatus Fallopii is also positioned quite differently, likely because of the enlarged anterior process; in *Poebrotherium*, the hiatus Fallopii is set far back on the tympanic face, a morphology that is unusual in artiodactyls (O’Leary 2010). In *Pa. primaevus*, the hiatus Fallopii occupies the more typical rostral position, similar to what is found in the oromerycids and vicuña. These differences support the taxonomic separation of *Paratylopus* and *Poebrotherium* and support our referral of FMNH PM 14560 to the latter. Indeed, given that the external morphology is quite similar, differences in the petrosal are perhaps some of the most obvious morphological distinctions between the two genera. An additional distinction may be the relationship between the rostral and caudal tympanic processes, which are located much closer together in *Pa. primaevus*. However, based on the sample of *V. vicugna*, the relationship between the processes can be highly variable within a species, so it is quite possible that the morphology of *Pa. primaevus* represents individual rather than intergeneric variation.

#### Stevenscamelus franki

Although the petrosal of *S. franki* is incomplete, there are differences that distinguish it from other early camelids. The most obvious distinction is the seeming lack of a ventromedial flange; the ventral border of the petrosal extends little below the promontorium, and certainly not below the rostral tympanic process. While there is also little extension beyond the process in *Pa. primaevus*, the depth of the posteromedial flange is much greater, and there is a change in orientation, suggesting that some remnant of a ventromedial flange is present, and the full flange may simply not have been preserved. There is no such indication of this in *S. franki*. The ventrolateral tuberosity of *S. franki* is also much smaller than that of the other early camelids, tending towards a morphology reminiscent of the vicuña. Furthermore, there is little-to-no indication of an anteromedial tuberosity, a feature that is often present in both the early camelids and *V. vicugna*. It is likely that the anterior process and the subarcuate fossa are also smaller in *S. franki*, although the poor preservation of the petrosal makes some of the morphology difficult to interpret. Likewise, while few details of the bony labyrinth are preserved, it appears that the anterior semicircular canal is small relative to the cochlea. The only differences in the ossicular chain of *S. franki* that might be of note are found in the malleus. The articular surface of the *S. franki* malleus is shallower than that of *Poebrotherium* and *Pa. primaevus*, and the neck of the *S. franki* malleus appears to be curved, whereas that of the *Poebrotherium* specimens is straight. The neck of the *Pa. primaevus* malleus also looks to be straight, but it could be broken proximal to where the curve would begin. Differences in the incus, such as the shape of the rostral face and relative length of the processus brevis, are most likely individual variation—a range of shapes were observed in *Poebrotherium*, including some similar to *Pa. primaevus* and *S. franki*. Only the stapes of *Poebrotherium* was preserved, so no comparisons can be made. The otic region morphology of other floridatragulines is not known, so it is presently not possible to determine whether the differences seen in *S. franki* support its referral to the subfamily, but there are enough morphological differences to support the removal of *S. franki* from *Poebrotherium* (Prothero et al. 2023).

#### Vicugna vicugna *and other extant camelids*

##### Petrosal

The *V. vicugna* petrosal has many similarities to that of *Camelus dromedarius* (described by O’Leary (2010)) and the early camelids, some of which, such as the expanded epitympanic wing, are likely synapomorphies of the family. Still, there are notable differences between *V. vicugna, C. dromedarius*, and early diverging members of the family. The fossa for the muscularis tensor tympani is more pronounced in *V. vicugna* and *C. dromedarius* (O’Leary 2010) and, unlike in early members of the family, it excavates the tegmen tympani. The *C. dromedarius* specimen examined by O’Leary (2010) has a section at the caudal part of the fossa where the bone is absent, exposing the facial canal. O’Leary (2010) referred to this as a “pit.” The bone in this area is also very thin in the vicuña, particularly in the juveniles.

Both the early and the living camelids have a vestibular fossula distinct from the fenestra vestibuli, but the morphology of the fossula differs. The morphology of the fossula and fenestra could not be reconstructed for *Pa. primaevus* and *S. franki*, but in *Poebrotherium*, the rostral rim of the vestibular fossula is almost flush with the border of the fenestra vestibuli while the caudal rim is greatly expanded, extending far posterior to the fenestra. The vestibular fossula of *V. vicugna* and *C. dromedarius* is more regularly oval, lacking the extreme caudal extension (O’Leary 2010). Indeed, the fenestra vestibuli often sits close to the posterior rim of the vestibular fossula, resulting in the fossula having more rostral than caudal expansion.

The anterior process of *V. vicugna* is much more pronounced than that of early camelids—it contributes to the border of the petrosal and is partially detached from the tympanic face. The anterior process of *Pa. primaevus* also contributes to the border of the petrosal, but it remains flush with the rest of the bone, and the anterior process of *Poebrotherium* is much smaller. The anterior process of *C. dromedarius* appears to be most similar to that of *Poebrotherium*, although slightly larger (O’Leary 2010).

Conversely, the ventrolateral tuberosity of both *V. vicugna* and *C. dromedarius* is much smaller than that of the early camelids (potentially barring *S. franki*). In *Poebrotherium* and *Pa. primaevus*, the ventrolateral tuberosity forms a large overhang above the epitympanic recess, whereas the area above the recess in *V. vicugna* is a flat wall. The mastoid region of *V. vicugna* and *C. dromedarius* is larger and extends farther caudally than in the early taxa; all of the taxa have a wedge-shaped mastoid region, but the “wedge” of *V. vicugna* is elongated whereas that of *Poebrotherium* is stubby. *C. dromedarius* may have an intermediate morphology (O’Leary 2010; Allouch et al. 2023).

Endocranially, the most notable difference is the shape of the subarcuate fossa. *Vicugna* and all early taxa have a large and spherical subarcuate fossa with a ventral fossula, but the location of the ventral fossula differs. In *Poebrotherium* and *Pa. primaevus*, it is an extension through the posterior semicircular canal, whereas in *V. vicugna*, it extends through the lateral semicircular canal. Whether this represents two independent derivations of a ventral fossula cannot be determined with the present sample. *Camelus* has either a shallow and conical subarcuate fossa or a subarcuate depression, depending on the individual (O’Leary 2010; Allouch et al. 2023; Robson and Theodor 2025).

*Poebrotherium* appears to have two endocranial ridges dorsal to the subarcuate fossa, one identified as the crista petrosa and the other identified as the subarcuate flange. *Vicugna* only has one ridge, which is interpreted to be the crista petrosa, the same as in *Protylopus* and *Eotylopus*. It is possible that the second ridge in *Poebrotherium* is *de novo* to the genus—the region could not be reconstructed with high enough fidelity in the other early camelids to definitively determine its presence (but see *S. franki* description)—but this may also be a morphology common in early camelids that has later been lost.

#### Bony labyrinth

The bony labyrinth of *V. vicugna* differs from that of early camelids in several notable ways. First, there is a much larger gap between the basal and second turn of the cochlea in *V. vicugna*. A gap is present in the earlier taxa but not to the same degree. Second, the vestibular aqueduct appears to be shorter in *V. vicugna*, exiting the petrosal close to the base of the common crus. Costeur et al. (2017) has suggested that the morphology of the vestibular aqueduct may have phylogenetic significance, but because the path of the vestibular aqueduct could not be completely reconstructed for most specimens, little more can be said on that front. Along with this, the vicuña bony labyrinth has between 2.50 and 3.25 turns, whereas the *Poebrotherium* bony labyrinth has between 3.00 and 3.75 turns, meaning that the *Poebrotherium* bony labyrinth is more coiled than that of *V. vicugna*. Whether the other early camelids had a similar number of turns cannot be determined at this time. Likely because of the additional turns, *Poebrotherium* also has a higher aspect ratio, ranging from 0.59 to 0.74 compared to the 0.48 to 0.57 of *V. vicugna*.

##### Ossicular chain

The morphology of the extant camelid ossicular chain was described by Doran (1878), primarily based on *C. dromedarius*, with additional description of *C. bactrianus* provided by Bai et al. (2009). Although the ossicles of the early camelids are not well preserved, their overall morphology is comparable to that of *V. vicugna, Camelus*, and *Lama glama* (=*Lama peruana* in Doran (1878)), and they lend some insight into the evolution of the camelid ossicular chain. The head of the malleus of *Poebrotherium, Pa. primaevus*, and *S. franki* is greatly expanded rostroventrally, in part because of a prominent capitular spine. *Camelus dromedarius* and *C. bactrianus* have a similar expansion (Doran 1878; Bai et al. 2009: fig. 5C), but Doran (1878) described *Lama glama* and *Vicugna pacos* as lacking this expansion. We concur with this assessment—*V. vicugna* also has a relatively reduced mallear head. In this way, the early camelids resemble *Camelus. Poebrotherium* has a small muscular process, as does *V. vicugna, Camelus*, and *V. pacos* (Doran 1878; Bai et al. 2009). However, *L. glama* apparently lacks a defined process (Doran 1878), indicating that it may have been secondary lost in the genus. An anterior process is known to be present in fetal *Lama* and *V. vicugna* (Maier and Ruf 2016), but we could not locate this process in the juvenile or adult vicuñas, nor any of the extinct taxa. In both *Poebrotherium* and the extant taxa, the manubrium is long and thin with apparently little change in thickness along its length and only slight curvature distally (Doran 1878; Bai et al. 2009), which suggests that this is likely the ancestral condition in camelids.

Despite being fairly similar in overall shape, there are some differences between the malleus of the extinct and extant camelids. The articular surface for the incus in *Poebrotherium* and *Pa. primaevus* is narrower and deeper than in the living taxa. The articular surface of *S. franki* also appears to be relatively narrow, but it is more similar to the extant taxa in depth. While this could be an artifact of the scan, it is worth noting that all *Poebrotherium* mallei had a deep articular surface regardless of scan quality. In all three extinct genera (*Poebrotherium, Paratylopus, Stevenscamelus*), the inferior articular facet is much smaller than the superior articular facet, giving the base of the mallear head a tapered appearance. While the inferior articular facet in the extant genera is smaller than the superior articular facet, the difference is not as great. In *V. vicugna*, the inferior facet is slightly shorter than the superior facet, but they are of equal height. In *C. dromedarius*, the two facets are the same length, but the inferior facet is narrower (Doran 1878). The extinct genera also have a longer mallear neck than the extant genera. A long mallear neck is also present in cainotheriids and ruminants, whereas suids and hippopotamids have a short neck more similar to that of the extant camelids (Doran 1878; Assemat et al. 2020).

Doran (1878, p.424) noted that the camelid incus is “very singular and distinctive in form” because of its narrow and elongate body and wide articular facets that terminate well above the root of the processus longus. *Camelus bactrianus* (Bai et al. 2009: fig. 5B–E) and our specimens of *V. vicugna* match this description, supporting Doran’s (1878) inference that this is likely the morphology of all living camelids. However, this is not the morphology of the early camelids. The body of the early camelid incus is wider and square rather than rectangular. The narrowness of the *V. vicugna* incus is partially caused by a deep depression ventral to the inferior articular surface. The same depression is present in *Poebrotherium*, but it is not as deep, so the depth of the depression may be taxonomically important. Because the body of the extant camelid incus is more elongate than that of the early camelids, the inferior articular facet of the early taxa ends closer to the processus longus. Additionally, in living camelids, there is no clear division between the rostral and lateral faces of the incus: the superior articular facet grades into the inferior articular facet, leading to the convexity of the incudal head observed by Doran (1878). The early camelids, *Poebrotherium* in particular, have very distinct superior and inferior articular facets. They are offset from each other and separated by a bar of bone, resulting in the rostral part of the incus being much more robust compared to that of *V. vicugna*. These differences make the incus of the early camelids more similar to *Sus scrofa* and some ruminants (Doran 1878). However, it is worth noting that the processus longus of the early camelids is consistently longer than the processus brevis. This is different from both suiforms and ruminants, which have a much longer processus brevis (Doran 1878).

The stapes of *V. vicugna* is largely similar to that of other living camelids, particularly the laminins (Doran 1878). Like *L. glama*, but unlike species of *Camelus*, the crura are symmetrical (Doran 1878; Bai et al. 2009). The only potential difference between *V. vicugna* and *L. glama* is that the articular facet of *V. vicugna* slopes rostrally. This slope is not illustrated for *L. glama* by Doran (1878), and it is not described as being present in any of the other extant camelids. UNSM ZM-16921 does not exhibit as strong of a slope as UCMZ (M) 1986.308 but unfortunately, because these two specimens are a juvenile and a potentially hybridized adult, it is not possible to determine whether this sloping morphology is individual variation, an effect of ontogeny, or diagnostic of *V. vicugna*.

Compared to the extant taxa, the *Poebrotherium* stapes is stout because of the shorter crura and larger stapedial head. The foramen intercrurale is also wider at the base than at the top, whereas the foramen intercrurale of the extant taxa forms a near-symmetrical oval. There is a clear processus muscularis stapedius for the stapedial muscle on the posterior crus in *Poebrotherium*. No such process is present in *V. vicugna* nor is one illustrated or described by Doran (1878), but a processus muscularis stapedius is clearly present in *C. bactrianus* (Bai et al. 2009), suggesting that this process is variably absent in the extant taxa. The *Poebrotherium* stapes somewhat resembles that of a hippopotamus or a suiform (Doran 1878; Orliac and Billet 2016).

### Variation within taxa

The petrosals of *Protylopus, Poebrotherium*, and *V. vicugna* exhibit clear intraspecific and bilateral variation. The most prominent examples of intraspecific (or intrageneric) variation are found in FMNH PM 14560, an aberrant individual of *Poebrotherium*, and UCMZ (M) 1986.307, one of the adult *Vicugna* specimens. The most notable example of bilateral variation is found in UNSM ZM-16921, another adult vicuña. This specimen may have been hybridized with *Lama guanicoe*, and the effects of hybridization cannot be ruled out, but bilateral variation is also present in FMNH PM 14560, so the phenomenon is not unique to UNSM ZM-16921.

Some aspects of intraspecific variation can be explained by associations between the petrosal and soft tissue structures. For example, the ventromedial flange roofs the passage for the inferior petrosal sinus, and bones (or their cartilaginous precursors) typically form around blood vessels (Novacek 1993), so the unusual ventromedial flange morphology of FMNH PM 14560 may have been the result of a change to the path of the inferior petrosal sinus. Similarly, the foramen acusticum superius and the hiatus Fallopii are intimately associated with the greater petrosal nerve, and the lack of an enclosed hiatus Fallopii on the left petrosal of UNSM ZM-16921 is almost certainly the result of the petrosal forming around the nerve.

Conversely, the subarcuate fossa and the endocranial ridge are examples of petrosal elements that may be associated with, but not influenced by, soft tissues. The subarcuate fossa often develops prior to being fully filled by the paraflocculus (McClure and Daron 1971; Sánchez-Villagra 2002), and the endocranial ridge is not known to be linked with a specific soft tissue structure (although an association with the cerebellum is almost certain). Therefore, the somewhat deformed subarcuate fossa aperture and endocranial ridge of FMNH PM 14560 may have formed independent of the soft tissues they interact with. A more pronounced example of subarcuate fossa reduction is present in *Protylopus*: one specimen (SDSNH 60369) has the very large and spherical subarcuate fossa found in *Eotylopus* and most camelids. However, the subarcuate fossa of SDSNH 40812 is much smaller, losing its caudal expansion and resembling a deep cone with a rounded base. An even more extreme example of subarcuate fossa reduction is found in UCMZ (M) 1986.307, a *V. vicugna* specimen. To our knowledge, the size of the paraflocculus has not been reported in South American camelids, but a paraflocculus is certainly present in *Camelus* (Ali et al. 2022), and the subarcuate fossa of *Camelus* is much smaller than that of *V. vicugna* (O’Leary 2010; Robson and Theodor 2025). This, in combination with what is known about subarcuate fossa development, renders it unlikely that a reduced paraflocculus resulted in the subarcuate fossa morphology of FMNH PM 14560, SDSNH 40812, and UCMZ (M) 1986.307. Rather, the petrosal morphology was likely established early in development and the paraflocculus expanded to fill the available space. It is possible, especially in the vicuña, that the paraflocculus is smaller than the subarcuate fossa volume. This is important because the relative size of the paraflocculus has been used to infer increased eye movement control in rodents (Bertrand et al. 2016), although the same association may not be present in all mammalian orders (Ferreira-Cardoso et al. 2017). The potential lack of association between paraflocculus size and subarcuate fossa volume in camelids means that similar inferences cannot be made for the family (and possibly, other artiodactyl families).

Some other unusual aspects of morphology may not have any association with soft tissues. The ventrolateral tuberosity overhangs the epitympanic recess and helps to roof the middle ear cavity—it is primarily in contact with other bones, not soft tissues. Therefore, the enlarged ventrolateral tuberosity of FMNH PM 14560 is unlikely to have been the direct result of soft tissues. Similarly, the medial caudal tympanic process usually contacts the paracondylar process, another bony protrusion, and does not directly interact with a soft tissue (MacPhee 1981).The medial caudal tympanic process of FMNH PM 14560 flares out as a small wing, which is a condition also found in three of the *V. vicugna* specimens and in *Pa. primaevus*, so while FMNH PM 14560 is the only specimen of *Poebrotherium* in the sample to have this morphology, it is not unusual for the family as a whole. The same is true of the rostral tympanic process of FMNH PM 14560, which is unusual for *Poebrotherium* in having a close association with the endocranial ventromedial flange but is the normal condition in the vicuña. Furthermore, both FMNH PM 14560 and UCMZ (M) 1986.307 have an equally reduced caudal endocranial process, indicating that some types of morphological disparity are likely ubiquitous in Camelidae.

The right petrosal of FMNH PM 14560 has an enlarged anterior process of the tegmen tympani, which is one of the more pronounced examples of bilateral variation in *Poebrotherium*. In cetaceans, the anterior process articulates with the ectotympanic (Luo and Gingerich 1999), and the same is true of *Poebrotherium*: the relative enlargement of the right anterior process simply serves to maintain contact with the auditory bulla. The relative expansion of the rostral tegmen tympani is less explicable because the medial portion does not come into contact with the bulla, but this is again a morphology also found in one specimen of *Vicugna* (UNSM ZM-16921), indicating consistent types of variation in camelids.

While UCMZ (M) 1986.307 appears to be normal in all other aspects, UNSM ZM-16921 may be a hybrid of *V. vicugna* and *L. guanicoe*, and FMNH PM 14560 could not be referred to a species because it has a combination of dental features characteristic of both *Po. eximium* and *Po. wilsoni*, along with an odd lower incisor morphology not previously known in early camelids. This means that two of the three specimens with the most extreme examples of intraspecific and bilateral variation are unusual in ways independent of the otic region. However, intraspecific variation is not limited to these individuals. For example, UCMZ (M) 1986.308, one of the juvenile vicuñas, has a hyperinflated tegmen tympani, and the shape of the rostral and caudal tympanic processes of *V. vicugna* appear to occur on a spectrum across individuals. From the sample included in this study, it is clear that there are some aspects of petrosal morphology that are far more variable than has been previously reported. The same is not true for the bony labyrinth, which is fairly conserved within species—given the functional constraints of the acoustic and vestibular apparatus, this is not surprising. The only notable example of bony labyrinth variation is the inconsistency between the angles of the semicircular canals within and between individuals, something that seemingly occurs in all taxa.

Given that many of the ear ossicles were poorly preserved, limited observations can be made about their intraspecific and intrageneric variability. However, the incus of FMNH PM 14560 does have a much squarer rostral face than that of other *Poebrotherium* specimens. Given the unusual morphology of the FMNH PM 14560 petrosal, it is perhaps unsurprising that this specimen also stands out in other aspects of the otic region. As discussed previously, there is also a slight difference between specimens in the articular surface of the vicuña stapes, but this difference cannot be attributed to intraspecific variation when ontogeny may be at play.

### Individual variation and morphological characters

The otic region is often thought to be a relatively slow evolving region of the skull (Goswami et al. 2023) that may preserve features useful for resolving deep evolutionary relationships (Cifelli 1982; Novacek 1986; Wible and Novacek 1988; Wible et al. 1995). However, this does not mean that the region is static, as evidenced by the morphological variation present in the petrosals of camelids. Characters such as the shape of the caudal tympanic process, the subarcuate fossa, and various aspects of the tegmen tympani are now incorporated in phylogenetic analyses (Spaulding et al. 2009; Orliac 2013; Mennecart and Costeur 2016a; Orliac et al. 2023) and, because otic region data are relatively scarce and both time-consuming and expensive to collect, a single representative of each taxon is often used when coding these characters. This is not a problem if morphology is conserved within a taxon, but it may become an issue if several highly variable characters are included but not properly documented. Including polymorphic characters is important (Watanabe 2016), and only sampling the most frequently occurring state can lead to well-resolved but highly inaccurate trees (Garbin et al. 2018), meaning that the use of single specimens to represent species and genera may inadvertently affect the results of phylogenetic analyses. Based on *Protylopus, Poebrotherium*, and *V. vicugna*, the petrosal of tylopods is more morphologically variable than previously thought: large amounts of intraspecific (and bilateral) variation were present in a sample of only two *Protylopus* specimens, six *Poebrotherium* specimens, and five *V. vicugna* specimens. This concurs with observations of variation in other artiodactyl clades (Orliac et al. 2017). Therefore, we suggest that, whenever possible, it is best to sample multiple individuals and, ideally, examine both the left and right petrosal to avoid these confounds.

## Conclusions

Based on data from the otic region, *Eotylopus* is almost certainly a close relative of camelids. The evidence is much less strong for *Protylopus*, but given that both taxa are clearly oromerycids, it is likely that Oromerycidae are indeed the sister family to Camelidae. There is little evidence to support *Bunomeryx* as an early branching tylopod—neither *Protylopus* nor *Eotylopus* have a petrosal morphology similar to *Bunomeryx*, and neither do camelids or protoceratids. Unfortunately, the unusual petrosal morphology of *Protylopus* only serves to make the origins of Tylopoda more ambiguous. This may be because there is sparse sampling of oromerycids, homacodontids, and oreodonts, but it may also be that *Protylopus* has a unique morphology not shared by closely related taxa. Very few oromerycids and homacodontids have intact cranial material, so it is simply not possible to sample a large number at present. However, many oreodont species are represented by intact crania, and a sampling of oreodonts, particularly early members, may lend clarity to the situation.

The results of this study have both positive and negative implications for the use of otic region data for phylogenetic inference. On the one hand, it appears that the otic region of late-stage juveniles is morphologically stable in tylopods, suggesting that juveniles can be evaluated without concern for ontogenetic confounds. However, the taxa examined here do not represent the largest tylopods, and it is possible that bone accretion is more of a confounding factor for genera such as *Camelus*. On the other hand, the petrosal appears to have a large amount of intraspecific variation, meaning that the use of single specimens to represent species and genera may affect taxonomic interpretations and the results of phylogenetic analyses. Some amount of intraspecific variation was known to be present in homacodontids (Robson and Theodor 2025) and the anoplotheriid *Diplobune minor* (Orliac et al. 2017), but the findings here indicate that this variation is widespread, and perhaps more extreme than previously thought, although some features are certainly more variable than others. Given this, a more thorough study of petrosal variation across artiodactyls may be beneficial. Of course, whenever possible, more than one representative should be included in phylogenetic analyses, but this is often not feasible. Even outside of phylogenetic analyses, using single specimens to make evolutionary inferences is often unavoidable—three of the six genera included in the present study are represented here by single individuals—so it is important to understand which features are more likely to be conserved within a taxon.

In the case of oromerycids and camelids, many of the features that are likely to be phylogenetically important, such as the presence of a rostral tympanic process and a ventromedial flange, and the overall size and shape of the ventrolateral tuberosity and anterior process, appear to be consistent within species (although the anterior process of the right FMNH PM 14560 petrosal is enlarged for the genus). However, some other features, such as the caudal endocranial process and the subarcuate fossa, may be phylogenetically informative but variable within species. Knowing this, it is possible to evaluate extinct camelids represented by single individuals and determine which features are most likely to be indicative of an evolutionary relationship and which might be individual variation. With a large enough dataset of both extinct and extant taxa, similar evaluations should be possible for other artiodactyl families, including those that are entirely extinct.

## Supplementary Material

obaf043_Supplemental_Files

## Data Availability

The µCT data and digital models that support the findings of this study are reposited in MorphoSource and are available upon request at https://www.morphosource.org/projects/000647880/temporary_link/RP3RHVcXv8jmGNY27ZEzCW5z?locale=en (temporary link valid until 02 December, 2025). These data are managed by the associated museums and may only be accessed with their approval. A full list of media DOIs can be found in Supplementary Material 6.
