# Supplementary material for: The Internal Otic Region of Oromerycids (Artiodactyla, Oromerycidae), Early Camelids (Artiodactyla, Camelidae), and the Vicuña (Artiodactyla, Camelidae), Including Notes on Intraspecific and Subadult Ontogenetic Variation
Source: Integr Org Biol. 2025 Nov 17;7(1):obaf043. doi: 10.1093/iob/obaf043 (PMC12713648; doi:10.1093/iob/obaf043)
Supplement: obaf043_Supplemental_Files [file obaf043_supplemental_files.zip › SM 1 - Scan parameters.docx]

| Specimen | Voltage (kV) | Amperage (µA) | Voxel size (µm) | Filter (mm) | Slices in stack |
| --- | --- | --- | --- | --- | --- |
| AMNH FM 9806 | 220 | 200 | 10.806 | Cu 0.5 | 1626 |
| AMNH FM 42298 | 160 | 61 | 35.15 | Al 1.0 | 3490 |
| AMNH FM 47077 | 120 | 66 | 35.5 | Al 1.0 | 4137 |
| AMNH FM 47394 | 130 | 61 | 31.951 | Al 1.0 | 1902 |
| AMNH FM 147015 | 130 | 61 | 35.5 | Al 1.0 | 3271 |
| FMNH PM 14560 | 180 | 230 | 40.085 | Cu 0.2 | 1814 |
| FMNH UC 465 | 180 | 230 | 39.666 | Cu 0.2 | 1949 |
| FMNH UC 493 | 180 | 230 | 37.672 | Cu 0.2 | 1959 |
| SDSNH 40812 | 130 | 61 | 32.66 | - | 1891 |
| SDSNH 60369 | 130 | 61 | 24.85 | Al 1.0 | 3281 |
| TMM VP 40504-149 | 120 | 180 | 72.9 | Al (size unknown) | 1921 |
| UCMZ (M) 1986.307 | 68 | 147 | 60.7 | - | 2496 |
| UCMZ (M) 1986.308 | 68 | 147 | 60.7 | - | 2496 |
| UCMZ (M) 1986.309 | 68 | 147 | 60.7 | - | 2496 |
| UCMZ (M) 1986.310 | 68 | 147 | 60.7 | - | 2496 |
| UNSM ZM-16921 | 225 | 205 | 115 | - | 1636 |

**Table S1.** µCT scanning parameters for data collected in this study.
