# Supplementary material for: The Internal Otic Region of Oromerycids (Artiodactyla, Oromerycidae), Early Camelids (Artiodactyla, Camelidae), and the Vicuña (Artiodactyla, Camelidae), Including Notes on Intraspecific and Subadult Ontogenetic Variation
Source: Integr Org Biol. 2025 Nov 17;7(1):obaf043. doi: 10.1093/iob/obaf043 (PMC12713648; doi:10.1093/iob/obaf043)
Supplement: obaf043_Supplemental_Files [file obaf043_supplemental_files.zip › SM 3 - Additional images and measurement details.docx]

**ADDITIONAL MEASUREMENT DETAILS**

Measurements with potential inaccuracy are discussed below and indicated in Supplementary Material 4 with an “Est.”.

***Eotylopus* sp. cf. *E. reedi*, AMNH FM 47394**

The height of the right lateral semicircular canal is based on the reconstructed apex. This might not be the true apex of the curve since the full canal could not be reconstructed. Similarly, the plane fit to the right lateral semicircular canal is only based on the reconstructed portion and the lateral ampulla. Angles associated with the canal should be considered estimates because of this. The right cochlea is also not highly resolved. The fenestra vestibuli area was measured based on the right petrosal. There was no clear vestibular fossula, so measurements were taken from the internal and external borders of the fenestra vestibuli. The crista interfenestralis width is an estimate because the border of the cochlear fossula was not fully distinct. Although the exterior of the left petrosal was damaged, enough was intact to take an estimated measurement of the fenestra vestibuli length and the promontorium length.

***Paratylopus primaevus*, AMNH FM 9806**

The right posterior and lateral semicircular canals are incomplete, and the widths may be slightly over- or underestimated as a result. The basal turn of the right cochlea could not be fully reconstructed, so we chose not to measure the angle between it and the lateral semicircular canal. The width of the left crista interfenestralis is an estimate because the boundary of the vestibular fossula could not be completely identified.

***Poebrotherium eximium*, AMNH FM 42298**

The left LSC was indistinct in parts, but we were able to follow the canal and reconstruct the complete arch. Even so, we note that the height and width of the left cochlea may be inaccurate. The apex of both cochleae is indistinct, so the length is an estimate.

***Poebrotherium eximium*, AMNH FM 47077**

Both cochlear lengths are estimated as the apex of the spiral could not be fully reconstructed. The right cochlea is slightly better resolved and likely more accurate to the true length. Measurements of the right fenestra vestibuli, vestibular fossula, and crista interfenestralis are estimates because there was no clear distinction between the fenestra vestibuli and the vestibular fossula.

***Poebrotherium* sp., AMNH FM 147015**

The width of the left fenestra vestibuli, vestibular fossula, and crista interfenestralis are estimates because the medial border of the fenestra is not reconstructed with high fidelity. The same goes for the right fenestra vestibuli and vestibular fossula and the fenestra vestibuli area of both petrosals.

***Poebrotherium* sp., FMNH PM 14560**

The border of the right fenestra vestibuli is jagged so the area is an estimate. The stapedial footplate area of the left stapes is also an estimate because the footplate is incomplete.

***Poebrotherium wilsoni*, FMNH UC 465**

The otic region is broken bilaterally so lengths and angles of the semicircular canals could not be determined, except for the width of the left lateral semicircular canal, which is an estimate. However, the lateral semicircular canals are well-enough preserved that an angle between the canal and the cochlea could be measured. The stapedial footplate area of the left stapes is estimated because the footplate is incomplete.

***Poebrotherium wilsoni*, FMNH UC 493**

The medial border of the left fenestra vestibuli could not be reconstructed, so the area of the fenestra vestibuli is an estimate based on an area taken by bridging the gap between the rostral and caudal sides. The right fenestra vestibuli is better, but there is still some uncertainty around the medial border and the area should also be considered an estimate.

***Protylopus* sp., SDSNH 40812**

The planes fit to the right cochlea and posterior and lateral semicircular canals are based on partially reconstructed structures. The widths of the left anterior and lateral semicircular canals are estimated based on broken section; the caudal portion of the anterior canal and the rostral portion of the lateral canal are incomplete. The length of the common crus of both bony labyrinths is estimated because the base of the left crus and the apex of the right crus were not fully reconstructed.

***Protylopus* sp. cf. *P. stocki*, SDSNH 60369**

The cochleae are poorly preserved and lengths are estimates. The left lateral semicircular canal is incomplete, but we were able to fit a plane based on the majority of the arch. This plane may be inaccurate because one end of the arch was not preserved, so the angle between the LSC and the cochlea may be incorrect. The length of the left common crus is potentially overestimated because the apex could not be confidently identified. The fenestra vestibuli length and width of both petrosals are based on the internal aperture of the fenestra vestibuli, and the length and area of the left fenestra vestibuli are estimates because the internal extent of the caudal border is not clear. The crista interfenestralis width and the promontorium length of the left petrosal are estimates because the petrosal is broken in this region.

***Stevenscamelus frank*, TMM VP 40504-149**

Both bony labyrinths are highly incomplete. A cochlear ratio was calculated, but the height of the cochleae might be underestimated because the apex is not distinct. The length of the promontorium was measured for both petrosals, but because the cochlear fossula is incomplete, it is possible that these measurements are a slight overestimate. The tip of the right lateral process of the epitympanic wing is also broken, so the length of the right epitympanic wing is also an estimate.

***Vicugna vicugna*, UNSM ZM-16921**

Because of the lower resolution of the scan, the left fenestra vestibuli could not be easily distinguished from the vestibular fossula. The lengths of the fenestra vestibuli and the vestibular fossula, along with the crista interfenestralis, should be taken as estimates. The right fenestra vestibuli area is likely to be underestimated.


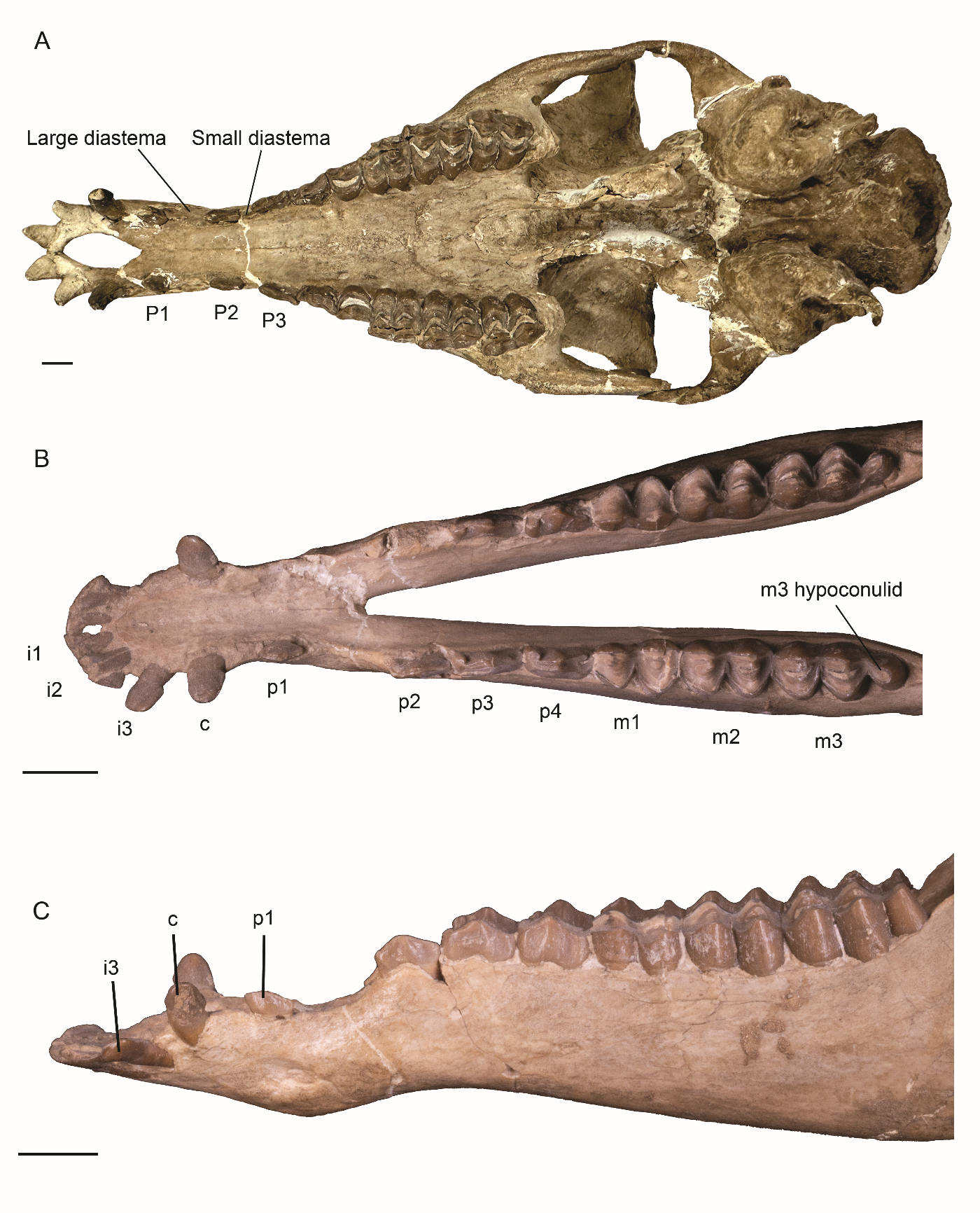


**Figure S1.** *Poebrotherium* sp. (FMNH PM 14560) **A**, cranium in occlusal view; **B**, lower jaw in occlusal view; **C**, lower jaw in left lateral view. Photos curtesy of W. Simpson. Scale bars are 1 cm.

**
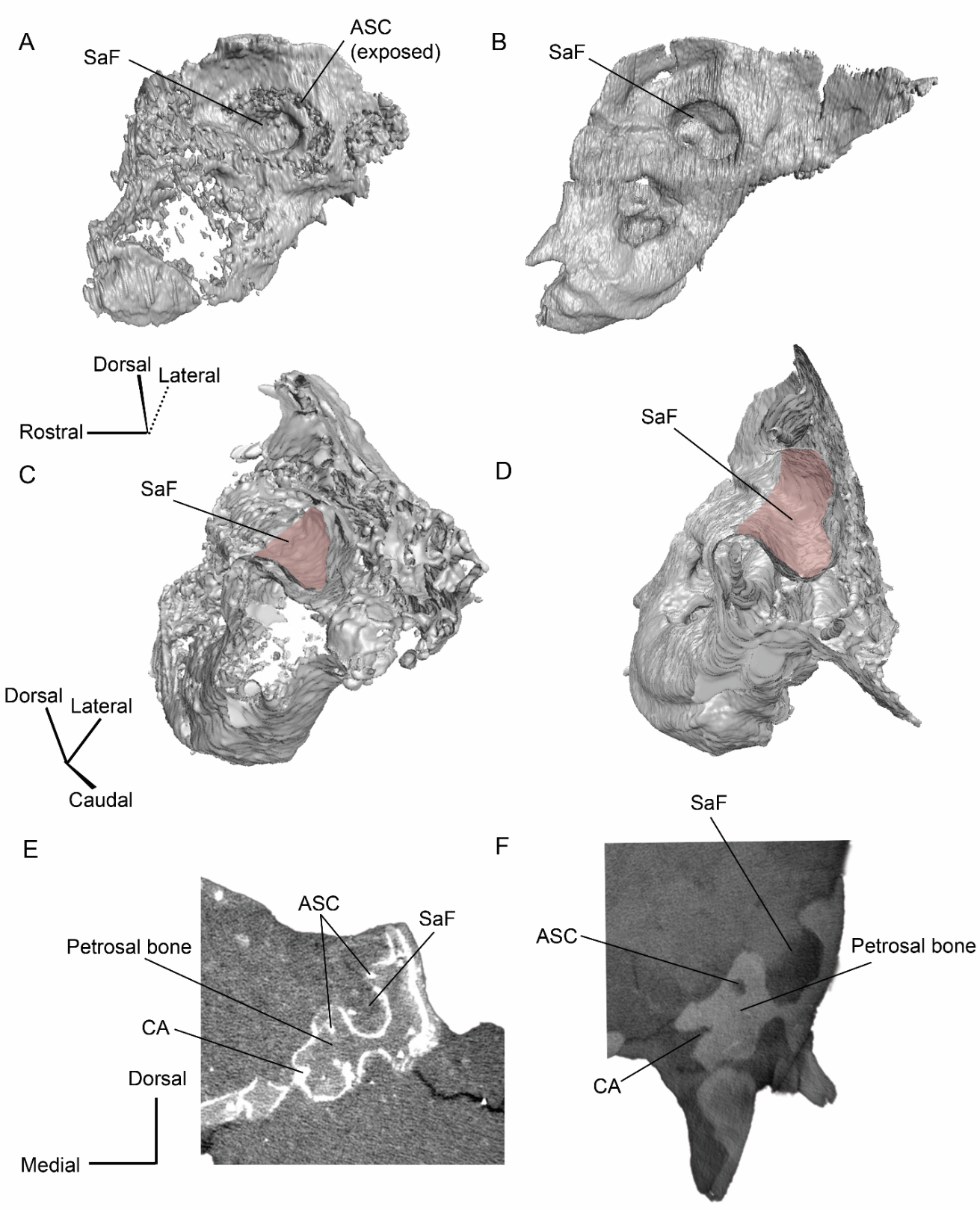
**

**Figure S2.** The right petrosals of *Protylopus* sp. (SDSNH 40812) (**A, C, E**) and *Protylopus* cf. *P*. *stocki* (SDSNH 60369) (**B, D, F**) in **A-B**, endocranial view; **C-D**, caudal view with posterior portion of bone removed to expose subarcuate fossa (colored in red); **E,** transverse CT slice 1920; **F**, transverse CT slice 1042. **Abbreviations: ASC**, anterior semicircular canal; **CA**, cochlear aqueduct; **SaF**, subarcuate fossa. Images not to scale.


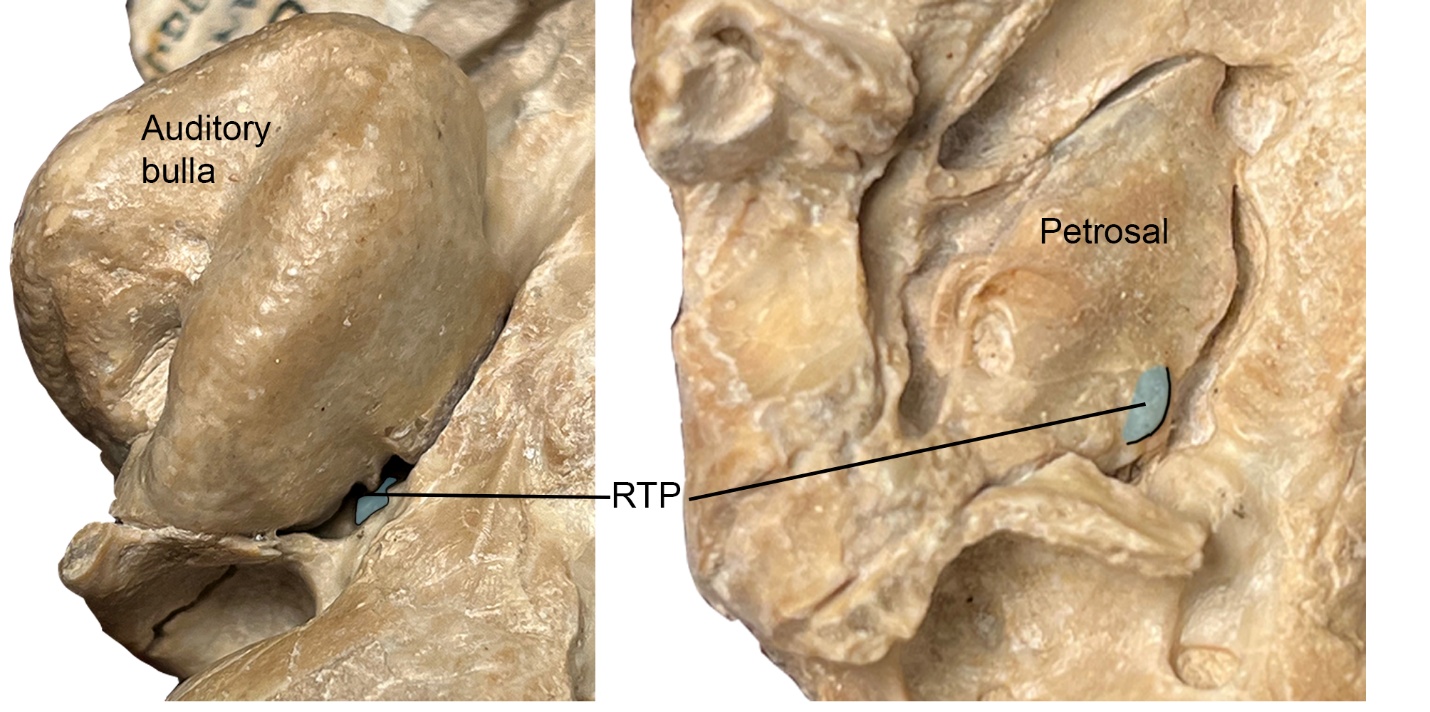


**Figure S3.** The right otic region of *Poebrotherium* sp. (AMNH FM 149343) with the auditory bulla articulated (left) and removed (right) showing the association between the audtiory bulla and the rostral tympanic process of the petrosal. Images not to scale. **Abbreviations: RTP**, rostral tympanic process.


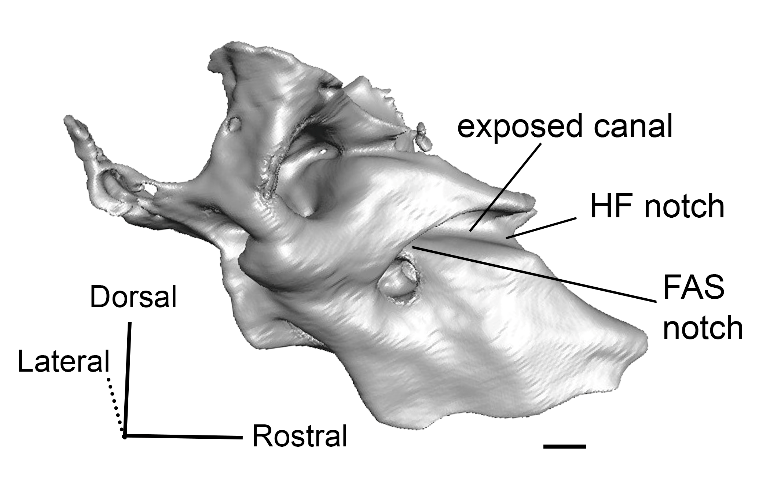


**Figure S4.** The right (mirrored) petrosal of UNSM ZM-16921, showing the unusual lack of an enclosed foramen acusticum superius. **Abbreviations: FAS**, foramen acusticum superius; **HF**, hiatus Fallopii. Scale bar 2 mm.


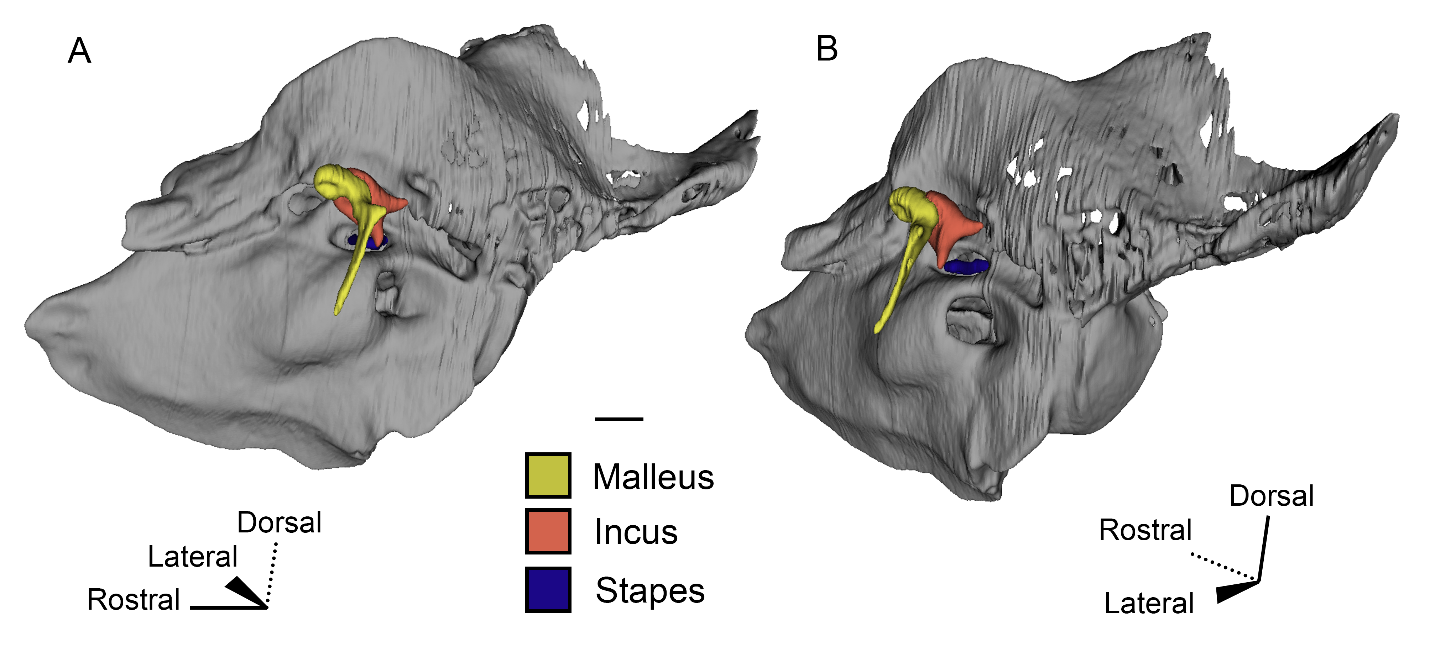


**Figure S5**. The left petrosal and ossicular chain of *Vicugna vicugna* (UCMZ (M) 1986.308) showing the relationships between the bones in **A**, tympanic and **B**, caudolateral view.


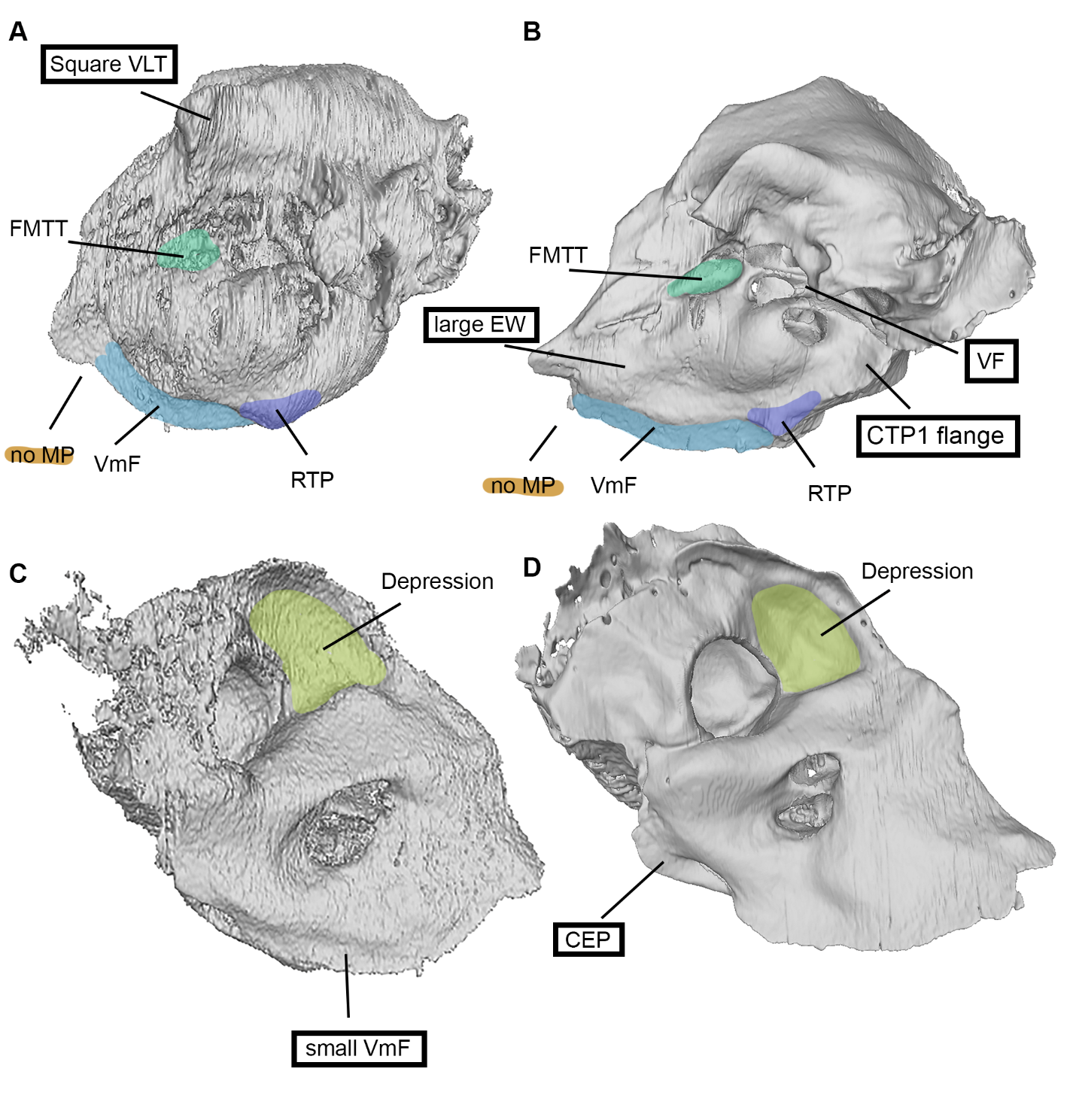


**Figure S6**. The right (mirrored) petrosal of the oromerycid *Eotylopus* cf. *E. reedi* (AMNH FM 47394) **(A, C)** and the left petrosal of the camelid *Poebrotherium wilsoni* (FMNH UC 493) **(C, D)** in tympanic **(A, B)** and endocranial **(C, D)** view. Colored features are similarities, boxed features are differences. **Abbreviations: CEP**, caudal endocranial process; **CTP1**, medial caudal tympanic process; **EW**, epitympanic wing; **FMTT**, fossa for the muscularis tensor tympani; **MP**, medial process of the epitympanic wing; **VF**, vestibular fossula; **VLT**, ventrolateral tuberosity; **VmF**, ventromedial flange. Images not to scale.
