# Supplementary material for: The Internal Otic Region of Oromerycids (Artiodactyla, Oromerycidae), Early Camelids (Artiodactyla, Camelidae), and the Vicuña (Artiodactyla, Camelidae), Including Notes on Intraspecific and Subadult Ontogenetic Variation
Source: Integr Org Biol. 2025 Nov 17;7(1):obaf043. doi: 10.1093/iob/obaf043 (PMC12713648; doi:10.1093/iob/obaf043)
Supplement: obaf043_Supplemental_Files [file obaf043_supplemental_files.zip › SM 5 - List of specimen ear ossicles.docx]

**Table S2.** Specimens with ear ossicles. **L**, left; **R**, right.

| Taxon | Specimen | Malleus | Incus | Stapes |
| --- | --- | --- | --- | --- |
| *Paratylopus primaevus* | AMNH FM 9806 | L | L | - |
| *Poebrotherium eximium* | AMNH FM 47077 | R | L/R | - |
| *Poebrotherium eximium* | AMNH FM 42298 | R | L/R | - |
| *Poebrotherium* sp. | AMNH FM 147015 | L | L | - |
| *Poebrotherium* sp. | FMNH PM 14560 | R | L/R | L |
| *Poebrotherium wilsoni* | FMNH UM 493 | - | - | R |
| *Poebrotherium wilsoni* | FMNH UM 465 | L | L | L |
| *Stevenscamelus franki* | TMM VP 40504-149 | R | R | - |
| *Vicugna vicugna* | UCMZ (M) 1986.308 | L/R | L/R | L/R |
| *Vicugna vicugna* | UCMZ (M) 1986.309 | L | - | - |
| *Vicugna vicugna* | UNSM ZM-16921 | L/R | L/R | L/R |
